# A Novel YY1-miR-1 Regulatory Circuit in Skeletal Myogenesis Revealed by Genome-Wide Prediction of YY1-miRNA Network

**DOI:** 10.1371/journal.pone.0027596

**Published:** 2012-02-01

**Authors:** Leina Lu, Liang Zhou, Eric Z. Chen, Kun Sun, Peiyong Jiang, Lijun Wang, Xiaoxi Su, Hao Sun, Huating Wang

**Affiliations:** 1 Department of Chemical Pathology, Li Ka Shing Institute of Health Sciences, The Chinese University of Hong Kong, Hong Kong, China; 2 Department of Obstetrics and Gynaecology, Li Ka Shing Institute of Health Sciences, The Chinese University of Hong Kong, Hong Kong, China; University of Massachusetts Medical, United States of America

## Abstract

microRNAs (miRNAs) are non-coding RNAs that regulate gene expression post-transcriptionally, and mounting evidence supports the prevalence and functional significance of their interplay with transcription factors (TFs). Here we describe the identification of a regulatory circuit between muscle miRNAs (miR-1, miR-133 and miR-206) and Yin Yang 1 (YY1), an epigenetic repressor of skeletal myogenesis in mouse. Genome-wide identification of potential down-stream targets of YY1 by combining computational prediction with expression profiling data reveals a large number of putative miRNA targets of YY1 during skeletal myoblasts differentiation into myotubes with muscle miRs ranking on top of the list. The subsequent experimental results demonstrate that YY1 indeed represses muscle miRs expression in myoblasts and the repression is mediated through multiple enhancers and recruitment of Polycomb complex to several YY1 binding sites. YY1 regulating miR-1 is functionally important for both C2C12 myogenic differentiation and injury-induced muscle regeneration. Furthermore, we demonstrate that miR-1 in turn targets YY1, thus forming a negative feedback loop. Together, these results identify a novel regulatory circuit required for skeletal myogenesis and reinforce the idea that regulatory circuitries involving miRNAs and TFs are prevalent mechanisms.

## Introduction

microRNAs (miRNAs) are non-coding single-stranded RNAs of 21 to 25 nucleotides and constitute a novel class of gene regulators that are found in many eukaryotic organisms. miRNAs negatively regulate their targets at the post-transcriptional level through binding to their 3′ UTR regions leading to either mRNA cleavage or translational repression depending on the degree of complementarities between the miRNA and the target [1]. In recent years, growing body of evidence supports the involvement of miRNAs as widespread regulators in a variety of biological processes as well as their roles in diverse pathologies. Of particular interest, mounting reports have demonstrated the regulatory roles of miRNAs in muscle development and muscle related diseases [2], [3], [4], [5], [6].

Skeletal muscle growth and regeneration are attributed to satellite cells-muscle stems cells, which are characterized by the expression of paired-box transcription factor 7 (Pax7) and when activated, become immature muscle cells or myoblasts which will proliferate and differentiate. The formation of mature muscle proceeds with the exit of myoblasts from the cell cycle, the expression of muscle-specific genes and the suppression of genes that are specific to other cell lineages and tissues [7]. A major portion of our understanding of myogenic differentiation is focused at the level of transcription, orchestrated by a complex network of transcription factors (TFs) including MyoD, Myogenic Factor 5 (Myf5), Myogenin, Myogenic Regulatory Factor 4 (MRF4), and myocyte enhancer factor 2 (Mef2). These factors activate muscle specific genes to coordinate myoblasts to terminally withdraw from the cell cycle and subsequently fuse into multinucleated myotubes [8]. Pax7, on the other hand, is required for satellite cell proliferation and sustained expression of Pax7 prevents myogenic induction and muscle terminal differentiation [9].

Studies from recent years have incorporated miRNAs into the complex network of regulation. A subset of these miRNAs, miR-1, miR-133, and miR-206, are muscle specific and have been the focus of intensive investigation [5]. For instance, MyoD and Mef2 regulate the expression of miR-1 that suppresses Histone Deacetylase 4 (HDAC4), resulting in augmented Mef2 activity [10], [11]. MyoD also induces miR-206 which targets DNA polymerase alpha to facilitate cell cycle exit and follistatin to presumably enhance myogenesis [12], [13], [14]. In contrast, synthesis of miR-133 inhibits myogenesis through the down-regulation of serum response factor (SRF) which maintains myoblasts in a proliferative state [15]. Thus miR-1, miR-133 and miR-206 play central regulatory roles in muscle biology and are called muscle miRs. In addition to the above muscle miRs, emerging evidences also support the involvement of non-muscle miRs in myogenesis. For example, Juan et al characterized a double-negative feedback loop comprising of miR-214 and a histone methyltransferase Ezh2 recently [16]. These studies clearly demonstrate the crucial roles of TF-miRNA circuitries in regulating gene expression. However, in myogenesis, the total number of miRNA genes reported to form the regulatory circuitries only accounts for ∼2% of all mouse miRNAs found in miRBase [17]. And the regulatory mechanisms of the majority miRNAs in myogenesis are still largely unknown.

In an effort to elucidate the transcriptional and post-transcriptional regulation during skeletal myogenesis, we identified an NF-KappaB (NF-κB)-Yin Yang1 (YY1)-miR-29 signaling axis. YY1, a ubiquitously expressed transcription factor, upon activation by NF-κB signaling, functions to repress muscle differentiation by epigenetically silencing the transcription of miR-29 expression through recruiting histone methyltransferase Ezh2 containing Polycomb complex as well as histone deacetylase 1(HDAC1) [18], [19]. At the onset of myogenesis, YY1 is down-regulated by decreased NF-κB signaling, which results in the replacement of YY1/Ezh2/HDAC1 repressive complex by an activator complex containing MyoD, serum response factor (SRF) along with associated acetyltransferases CBP and p300/CBP-associated factor (PCAF), leading to the activation of miR-29 [18], [20]. Our findings together with others' [20] highlight the essential roles of YY1 in the epigenetic regulation of myogenesis. Considering the prevalence of YY1 binding sites in the genome (∼70% of vertebrate genes and ∼24% of viral genes contained YY1 binding elements [21]), we speculate that many other miRNAs could come under regulation by YY1, forming numerous functional regulatory circuitries.

In this study we perform genome-wide identification of YY1 targeted miRNAs by combining computational prediction and expression profiling data. We further demonstrate that muscle miRs are regulated by YY1 and elucidate its underlying mechanism as well as functional significance both *in vitro* using C2C12 mouse muscle cells and *in vivo* using cardiotoxin induced muscle regeneration model.

## Results

### Computational prediction reveals the prevalence of YY1 binding sites in miRNA promoter regions

**Figure 1:**
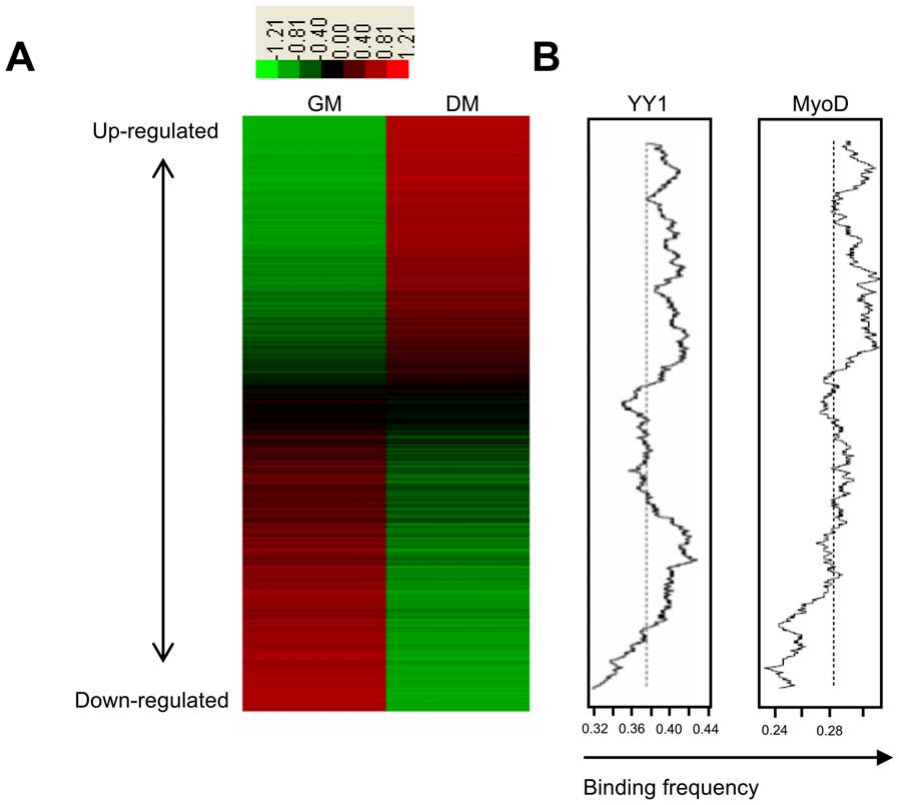
YY1 binding sites are enriched in both up- and down-regulated miRNAs. (A). miRNAs in DM cells are rank-ordered by the degree of up-regulation (red) and down-regulation (green) relative to GM cells. (B). The two plots (left and right) show moving average of the frequency of probes for genes that have YY1 or MyoD -binding sites in a 300-probe sliding window. The dashed line indicates the expected average (background level or the ratio of the number of probes for YY1 or MyoD targets over the total number of interrogated gene probes).

In order to identify novel YY1-miRNA regulatory circuits and gain insights into the magnitude of the number of YY1 regulated miRNAs, we developed a bioinformatics pipeline, mainly based on *ab initio* sequence analysis. The major limitation in studying transcriptional regulation of miRNA genes has been sparse annotation of miRNA gene transcriptional start sites (TSSs) and promoter regions. Marson *et al.* took advantage of genome-wide mapping of trimethylation of histone H3 lysine 4 mark, which associates with TSSs of most genes, to systematically identify the promoter regions for over 80% of miRNAs in both mouse and human [22]. The annotation of 185 murine primary microRNA (pri-miRNA) transcripts specifying 336 mature miRNA was retrieved from this study. We further reasoned that histone mark based annotation is only a coarse estimation of promoter location, we thus decided to refine it using a promoter prediction program EP3 [23]. This is a tool for identification of the core promoter of a eukaryotic gene by using GC content and large-scale structural features of DNA. When evaluated on 12 eukaryotic genomes, EP3 demonstrated high performance. Subsequently, we applied EP3 on the above 185 mouse pri-miRNA transcripts to obtain the refined promoters for these miRNAs; the refined promoter regions were then extended by 15 kbp on both sides and subjected to YY1 binding sites search. Four YY1 positional weight matrices were retrieved from Transfac database [24] and used to scan for YY1 sites with STORM program [25] (Suppl. Table S1). Furthermore, we reasoned that functional YY1 regulation on miRNA genes should lead to silencing of their expression in myoblasts and up-regulation of miRNA expression levels upon myoblasts differentiation as YY1 is known as a repressor of muscle genes and YY1 itself is gradually down-regulated in myogenic differentiation [19]. We thus performed a microarray study to identify differentially expressed miRNAs during myogenic differentiation of mouse C2C12 myoblast cells. Total RNAs were isolated from undifferentiated C2C12 myoblasts in growth medium (GM) or myotubes formed in differentiation medium (DM) for 1 or 3 days and subjected to miRNA array analyses using microRNA CHIPv4 [26]. Although miRNA profiling during C2C12 differentiation has been conducted by Wong C.F. et al [27], our array platform contains a larger number of mouse miRNAs (373 vs 208). As a result, we found that 77 and 68 miRNAs in DM were up- and down-regulated more than two folds as compared with GM (Suppl. Table S2 and S3). This is significantly more than what was found by Wong C.F. (5 up-regulated and 1 down-regulated). By running the 77 up-regulated miRNA genes through YY1 binding site search pipeline described earlier, we were able to identify 30 miRNAs with at least one YY1 binding site on their promoters (Table 1). Interestingly, YY1 bindings sites were also frequently found on the promoters of miRNAs with high expression levels in myoblasts but down-regulated during differentiation (Suppl. Table S4), suggesting that YY1 can also function as a potential activator for these miRNAs to maintain their expression in myoblasts. To visualize the level of YY1 binding site enrichment, we ranked miRNAs according to their expression levels during differentiation so that the up-regulated and down-regulated miRNAs were grouped to the top and bottom of the panel, respectively and the unchanged ones located in the middle portion (Fig. 1A). The YY1 binding frequency in a sliding window from the up- to down-regulated genes was then calculated using a method described by Fei T [28]. It was found that YY1 binding sites were over-represented in both up- and down-regulated miRNAs but not in those without significant changes (Fig. 1B, left), supporting the notion that YY1 can potentially target these miRNAs, which leads to transcriptional activation or inhibition. When the same method was applied to MyoD, it was found that MyoD binding sites were over-represented only in up-regulated miRNAs (Fig. 1B, right), consistent with its accepted function as an activator in myogenesis. Interestingly, using miRanda, a miRNA target prediction program, we found that some YY1 regulated miRNAs could potentially target YY1, thus forming negative feedback loops (data not shown). This observation is in line with the belief that TF-miRNA feedback loops commonly exist and provide more coordinate and adaptable control for TF-miRNA circuitry to regulate gene expression [29], [30].

**Table 1:** Up-regulated miRNAs with predicted YY1 binding sites on promoter.

miRNA	Fold Chang (DM/GM)	P value	Predicted No. of YY1 binding sites
mmu-miR-133b	73.53	<10−4	1
mmu-miR-133a-1	68.49	<10−4	1
mmu-miR-206	40.16	<10−4	1
mmu-miR-1-2	11.78	4×10−4	1
mmu-miR-196a-2	9.25	6×10−4	5
mmu-miR-192	9.44	7×10−4	6
mmu-miR-322	7.26	<10−4	20
mmu-miR-24-2	5.59	<10−4	21
mmu-miR-409	7.03	<10−4	4
mmu-miR-221	7.05	<10−4	33
mmu-miR-30e	4.31	0.01	4
mmu-miR-320	3.65	0.01	7
mmu-miR-9-3	3.52	0.01	21
mmu-miR-351	3.29	0.02	14
mmu-miR-199a-1	3.11	0.02	8
mmu-miR-380	3.04	0.02	4
mmu-miR-425	3.07	0.02	7
mmu-miR-130b	3.04	0.02	3
mmu-miR-145	3.03	0.03	12
mmu-miR-214	2.88	0.03	28
mmu-miR-138-2	2.86	0.03	1
mmu-miR-30a	2.66	0.03	3
mmu-miR-126	2.75	0.04	1
mmu-miR-200b	2.73	0.04	1
mmu-miR-15a	2.69	0.04	4
mmu-miR-153	2.71	0.04	1
mmu-miR-381	2.73	0.04	4
mmu-miR-130a	2.71	0.04	23
mmu-miR-154	2.69	0.04	4
mmu-miR-451	2.34	0.05	1

**Genome-wide computational prediction reveals the prevalence of YY1 binding sites on promoters of miRNAs.** C2C12 myoblasts were grown in growth medium (GM) or differentiated in differentiation medium (DM) for 3 days. Total RNAs were extracted and subjected to expression profiling using a microarray platform. 77 miRNAs were found to be up-regulated in DM compared to GM with a fold change (DM/GM) more than 2. These miRNAs were run through the YY1 binding site search pipeline. A total of 30 miRNAs were found to contain at least one YY1 binding site on their promoters.

### YY1 negatively regulates muscle miR expression during myogenesis

**Figure 2:**
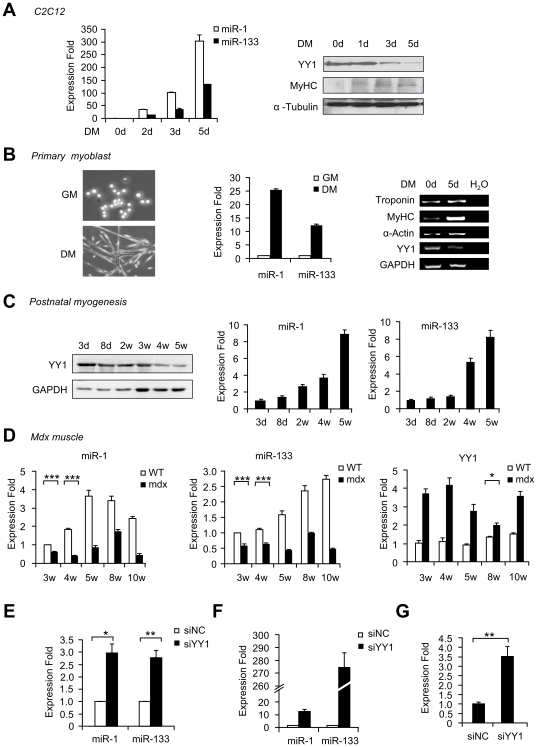
YY1 negatively regulates miR-1/133 expression both *in vitro* and *in vivo*. (A) C2C12 myoblasts were grown in growth medium (GM) or differentiation medium (DM) for 2, 3 or 5 days. Total RNAs or proteins were extracted and used for real-time RT-PCR assay (left) or Western blot analysis (right panel), respectively. (B) Primary myoblasts were isolated from limb muscles of 1 week old C57/BL6 mice and maintained in GM or induced to differentiate in DM. Cell morphology was visualized under light microscopy (left). Real-time PCR was performed to measure the expression levels of miR-1 and miR-133 normalized to U6 (middle). Semi-quantitative RT-PCR analysis was performed to measure the expression levels of Troponin, MyHC, α-Actin and YY1. Water (H_2_O) was used as negative control and GAPDH was used as a normalization. (C) Total proteins were isolated from lower limb muscles at post-natal day (P) 3 and 8 or tibialis anterior (TA) muscles from 2, 4, or 5 week old C57/BL6 background mice and Western blotting was used to probe for YY1 protein expression with GAPDH as a loading control (left). Total RNAs were isolated and qRT-PCR was subsequently performed to measure the expression of miR-1 and miR-133, normalized to U6 (middle and right). Expression folds are shown with respect to 3 day old mice where miR-1 and miR-133 levels were set to a value of 1. (D) TA muscles were isolated from 3 w, 4 w, 5 w, 8 w and 10 w old C57BL/6 wild type mice or *mdx* mice. RNAs were extracted and used for qRT-PCR assay of miR-1 (left), miR-133 (middle) or YY1 (right). Expression folds are shown with respect to wild type where miR-1, miR-133 or YY1 levels were set to a value of 1. (E) C2C12 myoblasts or (F) primary myoblasts were transfected with either negative control (siNC) or siRNA oligos against YY1 (siYY1). Cells were then cultured for 48 hours, at which time miR-1 and miR-133 expressions were measured by qRT-PCR and normalized to U6. Expression folds are shown with respect to siNC where miR-1 and miR-133 levels were set to a value of 1. (G) Expression of the primary transcripts of miR-1-2/miR-133a-1 was detected by qRT-PCR in C2C12 transfected with siYY1 or siNC oligos, and normalized to GAPDH. All quantitative data are represented as mean ± S.D. The p value was determined by Student's T-test: *p<0.05, **p<0.01, ***p<0.001.

Among all the putative YY1 target miRNAs, miR-1 and miR-133 families are particularly attractive considering the pivotal roles of these muscle miRs in regulating myogenesis [5]. We thus examined the potential regulation of muscle miRs by YY1 in C2C12. Total RNAs were collected from cells differentiated (DM) for 0, 1, 3 or 5 days and used for measuring miR-1 and miR-133 expression levels. Consistent with others' findings [15], both miR-1 and miR-133 were found to be robustly up-regulated during C2C12 differentiation whereas YY1 expression was gradually down-regulated (Fig. 2A). To strengthen the above findings, primary myoblasts were isolated from hind limb muscles of neonatal mice and grown in GM or differentiated in DM. As shown in Fig. 2B, when induced to differentiation by serum withdrawal, a sharp increase of both miR-1 and miR-133 expression was detected compared to GM cells. This up-regulation was concomitant with the down-regulation of YY1 as well as the induction of myogenic markers, Troponin, Myosin Heavy Chain IIb (MyHC) and alpha Actin (α-Actin). To substantiate the above results from cell culture studies, we extended the investigation to *in vivo* muscles. The first model employed was postnatal muscle development. In agreement with our previous findings [19], YY1 expression level decreased gradually as mice grow from 3 d to 5 w old (Fig. 2C, left). As expected, both miR-1 and miR-133 levels were up-regulated (Fig. 2C, middle and right), showing an inverse relationship with YY1 expression. We then examined this regulation in *mdx* muscles. As the commonly used mouse model to study Duchenne muscular dystrophy, this mouse carries the mutant dystrophin protein which leads to fiber necrosis, immune cell infiltration and defected regeneration. Muscles from wild type (WT) or *mdx* mice aging from 3 to 10 weeks were collected and examined for miR-1 and 133 expressions by qRT-PCR. A decrease of both miR-1 and 133 was detected in *mdx* muscles compared to age-matched WT mice (Fig. 2D, left and middle). However, YY1 expression level was found to be up-regulated at all time points examined (Fig. 2D, right), indicating that the down-regulation of miR-1 and miR-133 could be caused by elevated YY1 levels. Collectively, our data from the above studies suggest that YY1 negatively regulates miR-1 and miR-133 expression both in physiological and pathological muscle conditions.

To further test the above notion, C2C12 myoblasts were transfected with siRNA oligos knocking down YY1 (siYY1) or negative control (siNC) oligos (Suppl. Fig. S1). By qRT-PCR assay, both miR-1 and miR-133 levels increased over two folds upon YY1 depletion (Fig. 2E), suggesting that there is a negative regulation of miR-1 and miR-133 by YY1. When performed in primary myoblasts, knocking down of YY1 led to an even more significant increase of miR-1 and miR-133 (13.5 and 273 fold, respectively) (Fig. 2F). To confirm that this regulation occurs directly on transcriptional level, the level of primary transcripts was assessed. The miR-1-2/miR-133a-1 cluster is transcribed from a bicistronic miRNA precursor on the antisense strand of the Mindbomb (Mib) gene on mouse chromosome 18 and the primary transcript has been cloned [31]. Similar to mature miR-1 and miR-133, the level of pri-miR-1/133 transcripts was found to be induced upon siYY1 knockdown (Fig. 2G). The above findings thus confirm the presence of a transcriptional repression of miR-1 and miR-133 by YY1.

Since YY1 is a transcriptional target of NF-κB [19], we reasoned that miR-1 and miR-133 should also come under negative control of NF-κB. Consistent with this thinking, treatment of C2C12 myoblasts with TNFα as an activator of NF-κB reduced miR-1 and miR-133 expression (Suppl. Fig. S2A). Conversely, myoblasts expressing the IκBα-SR inhibitor of NF-κB led to higher levels of miR-1/133 over that of control cells (Suppl. Fig. S2B). These findings suggest that miR-1 and miR-133 are subjected to regulation by NF-κB-YY1 signaling.

### YY1 regulates muscle miRs through multiple enhancers

**Figure 3:**
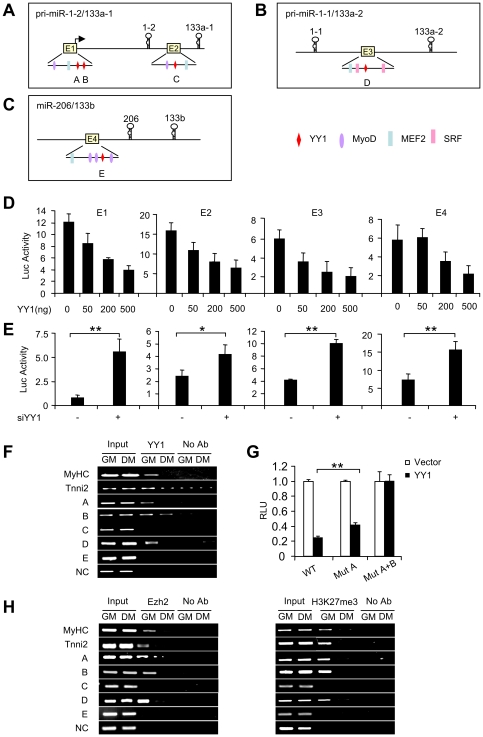
YY1 repression of miR-1/133 is mediated through multiple enhancers. (A) Two conserved enhancers (E1 and E2) were identified in the promoter region and intragenic region of miR-1-2/miR-133a-1 cluster, respectively. Three putative YY1 binding sites, A, B and C, were identified. (B) One conserved enhancer (E3) was identified in between miR-1-1 and miR-133a-2 with a putative YY1 binding site, D, identified. (C) One conserved enhancer (E4) was identified upstream of miR-206 and miR-133b cluster with a putative YY1 binding site, E, identified. Binding sites for MyoD, MEF2 and SRF were also shown. (D) C2C12 cells were transfected with 250 ng of E1, E2, E3 or E4 reporter plasmid along with Renilla and control vector (YY1 0 ng) or 50 ng, 200 ng, 500 ng YY1 expressing plasmid. Cells were then cultured for 48 h at which time luciferase activities were determined and normalized to Renilla protein. The data represent the average of three independent experiments ± S.D. (E) C2C12 cells were transfected with 0.25 µg of E1, E2, E3 or E4 reporter plasmid along with Renilla luciferase vector and siYY1 or siNC oligos. Luciferase activity was determined as in (D). (F) Chromatins were harvested from C2C12 myoblasts growing in growth medium (GM) or myotubes maintained in differentiation medium (DM) and subjected to ChIP-PCR analysis. Primers were designed to amplify regions encompassing putative YY1 binding sites A, B, C, D, or E. MyHC and Tnni2 are known YY1 targets and used as positive controls. A genomic region that contains no YY1 binding sites was included as a negative control (NC). (G) Site A (Mut A) or both A and B (Mut A+B) were mutated in E1 luciferase reporter plasmid and luciferase reporter assay was performed to measure the response of mutants to YY1 over-expression as in (D). Relative luciferase unit (RLU) is shown with respect to Vector transfection where luciferase activities were set to a value of 1. (H) ChIP-PCR for Ezh2 or H3K27me3 was performed as in (F). The p value was determined by Student's T-test: *p<0.05, **p<0.01, ***p<0.001.

In order to further investigate the transcriptional regulatory mechanisms of muscle miRs by YY1, we scrutinized their genomic sequences for potential regulatory regions. Muscles miRs constitute two distinct families, the miR-1 family (miR-1-1, miR-1-2, and miR-206) and the miR-133 family (miR-133a-1, miR-133a-2, and miR-133b). As mentioned earlier, miR-1-2/miR-133a-1 cluster is transcribed from a bicistronic miRNA precursor on the antisense strand of Mib gene on mouse chromosome 18. Two enhancers were identified based on genomic sequence conservation (Fig. 3A, E1 and E2). E1 is located approximately 2 kb upstream of miR-1-2 while E2 is located in an intron separating miR-1-2 and miR-133a-1 coding regions. It was demonstrated that E2 is essential to direct miR-1-2/miR-133a-1 expression in the somite myotomes and through the skeletal musculature in embryogenesis [31]. A MEF2 site and a MyoD-binding E-Box are required for the skeletal muscle expression of this enhancer. miR-1-1/miR-133a-2 cluster is transcribed from non-coding regions on mouse chromosome 2. A 4.3 kb muscle-specific enhancer encompassing MEF2, MyoD and SRF binding sites, was previously identified between miR-1-1 and miR-133a-2 coding regions (Fig. 3B) [10], [31]. When applied to miR-206/miR-133b cluster of muscle miRs, a previously unknown enhancer was also identified at 770 bp upstream of miR-206/miR-133b cluster (Fig. 3C). And MyoD and myogenin occupancy have been detected in this region by ChIP analyses [10].

Next, by using a scan for putative YY1 binding site, CCATNTTN, using position weight matrices from TRANSFAC database, we were able to identify a total of five conserved sites with A and B located in E1 region, C in E2, D in E3 and E in E4 region (Fig. 3A–C), suggesting that these enhancer regions may mediate YY1 repression on muscle miRs. To test this notion, we cloned each enhancer region into the pGL3 luciferase reporter vector. The resultant E1, E2, E3 or E4 luciferase reporter plasmids were transfected into C2C12 cells along with an YY1 expression plasmid (50, 200 or 500 ng). Results showed a dose-dependent inhibition of each reporter activity by YY1 over-expression (Fig. 3D). Conversely, co-transfection of these reporters with siYY1 oligos led to an increase of each reporter activity (Fig. 3E). Consistent with previous reports, E1 reporter activity was enhanced by MyoD and MEF2 over-expression (Suppl. Fig. S3). These data suggest that YY1 functions to repress E1, E2, E3 and E4 activities.

To test whether YY1 repression is mediated through the above identified putative sites A to E, ChIP assay was performed against YY1 using chromatins isolated from proliferating C2C12 myoblasts in GM or myotubes formed in DM. Primers were designed to amplify regions encompassing sites A, B, C, D or E, respectively. MyHC or Tnni2 promoter were known to be bound by YY1 in myoblasts and used as positive controls. And a region with no YY1 site was used as negative control (NC). Results in Fig. 3F demonstrated that a positive binding was detected on MyHC and Tnni2 promoters in myoblasts and disappeared in myotubes as previously shown [19]. Similarly, in myoblasts an association with YY1 was found on sites A, B, D and the signal was lost or diminished in myotubes, suggesting that these three sites are competent for YY1 binding in myoblasts and YY1 is displaced upon myogenic differentiation. On the other hand, no binding was detected on sites C and E despite the fact that E2 and E4 are responsive to YY1 modulation. It is likely that other YY1 binding sites exist in these two regions and they are not predicted by the computational algorithm under stringent criteria. Alternatively, YY1 may be recruited to these regions indirectly through a co-factor, bypassing the need for a YY1 binding site. To substantiate the above ChIP data, we mutated site A in E1 Luciferase reporter plasmid (Mut A). As expected, this mutation led to a slight rescue of the repressed reporter activity by YY1 over-expression (Fig. 3G). Further mutation of site B (Mut A+B), however, led to full rescue of the inhibition by YY1, suggesting that sites A and B function synergistically in mediating YY1 suppression. Taken together, the above results suggest that sites A, B and D represent the binding elements that mediate YY1 effect on E1and E3 regions. However, since no positive binding was found on sites C and E, the true binding sites await to be identified.

Since YY1 transcriptionally represses miR-29b/c through recruitment of the Polycomb complex and the resultant trimethylation of H3K27 [18], we asked whether similar regulation occurred on muscle miRs loci. Indeed, in addition to YY1, we also detected binding for Ezh2 and its trimethylated activity on H3K27 on sites A, B and D but not on C and E (Fig. 3H). And like YY1 binding, the association was lost in myotubes. Collectively, these data suggest that YY1 regulates muscle miRs through recruitment of the Polycomb complex to multiple enhancer regions similar to its regulatory mode on miR-29b/c promoter.

### YY1 regulates the pro-myogenic function of miR-1 during C2C12 differentiation

**Figure 4:**
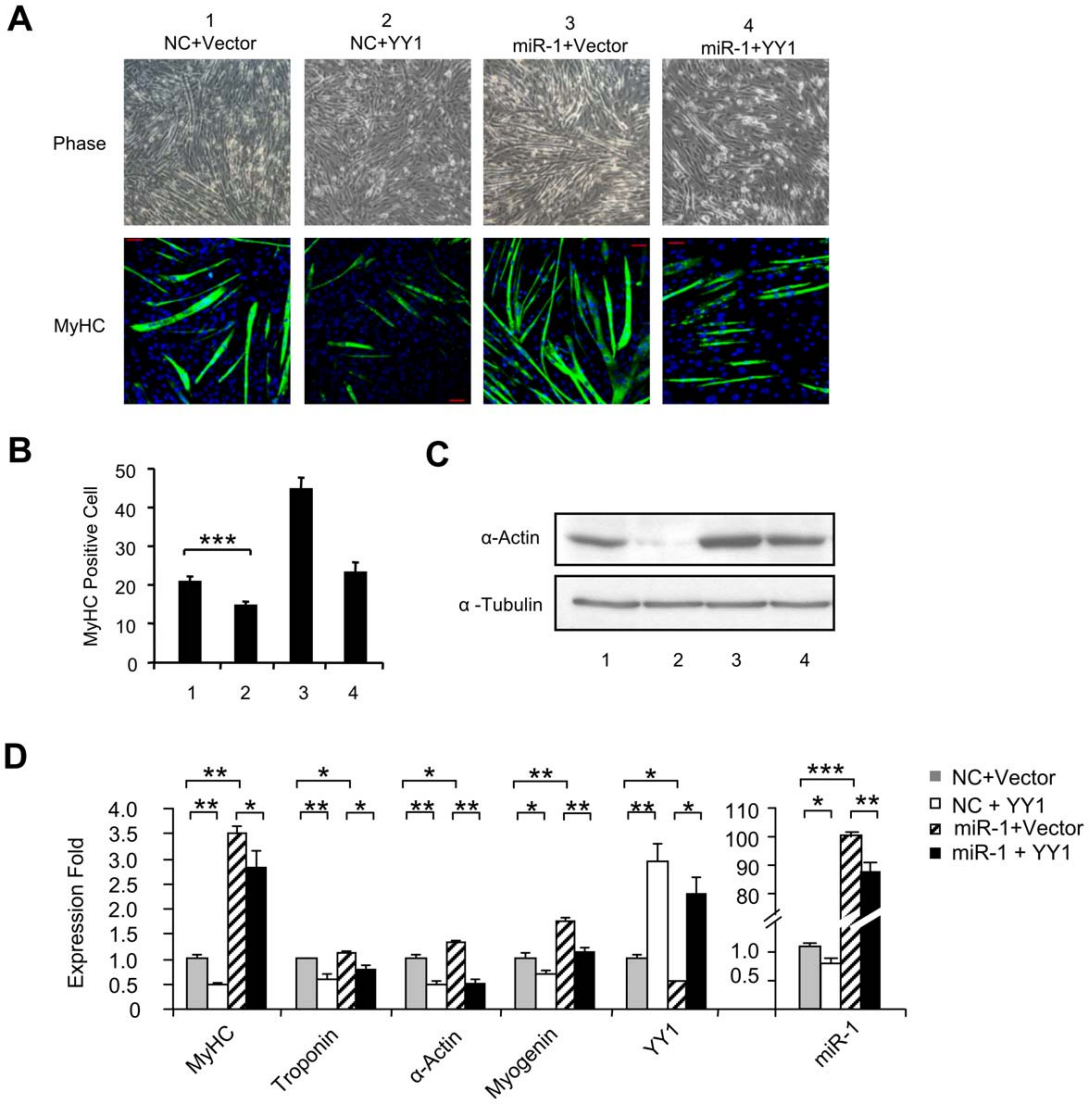
YY1 represses miR-1 functionally during C2C12 myogenesis. (A) C2C12 cells were transfected with the indicated combination of NC or miR-1 oligos and Vector or YY1 expression plasmids. Cells were then differentiated (DM) for 2 days, at which time cells were immunostained for MyHC. Cell morphology was visualized by phase-contrast microscopy. (B) MyHC positive cells were quantified by counting positively stained cells from 10 randomly chosen fields and are represented as mean ± S.D. (C) Total proteins were isolated from the above transfected cells and Western blotting was performed to probe for α-Actin. α-Tubulin was used as a loading control. (D) Total RNAs were extracted from the above transfected cells and used for qRT-PCR analysis of myogenic markers, MyHC, Troponin, α-Actin, and Myogenin normalized with GAPDH. YY1 and miR-1 levels were also measured to show the transfection efficiency. Quantitative values are represented as mean ± S.D. The p value was determined by Student's T-test: *p<0.05, **p<0.01, ***p<0,001.

Next, to address the functional significance of miR-1 regulation by YY1, we assessed the effect of YY1 expression on the pro-myogenic activity of miR-1 during C2C12 differentiation. C2C12 myoblasts were transfected with miR-1 oligos only or co-transfected with miR-1 oligos and an YY1 expression plasmid. Cells were then induced to differentiate for 2 days. The degree of differentiation was assessed by examining the expression of MyHC by immunofluroscence staining (Fig. 4A and 4B) or the expression of α-Actin proteins by Western blotting (Fig. 4C). In agreement with the known pro-myogenic function of miR-1, over-expression of miR-1 (Lane 3: miR-1+Vector) accelerated the myogenic program as shown by the significantly higher number of MyHC-positive cells (Fig. 4A and 4B) and increased expression of α-Actin (Fig. 4C) than that of control cells (Lane 1: NC+Vector). On the contrary, YY1 over-expression (Lane 2: NC+YY1) inhibited myogenic differentiation (Fig. 4A, 4B and 4C, Lane 2 compared to Lane 1), which is in line with our previous findings demonstrating YY1 as a repressor of myogenesis [18], [19]. As expected, YY1 over-expression on top of miR-1 (Lane 4: miR-1+YY1) suppressed the pro-myogenic activity of miR-1 (Fig. 4A, 4B and 4C, Lane 4 compared to Lane 3). Results from analysis of myogenic markers, MyHC, Troponin, α-Actin and Myogenin at RNA levels were in agreement with the above findings (Fig. 4D). Together, these data suggest that YY1 functions upstream of miR-1 in regulating its pro-myogenic action.

### YY1-miR-1-Pax 7 axis during myogenesis

**Figure 5:**
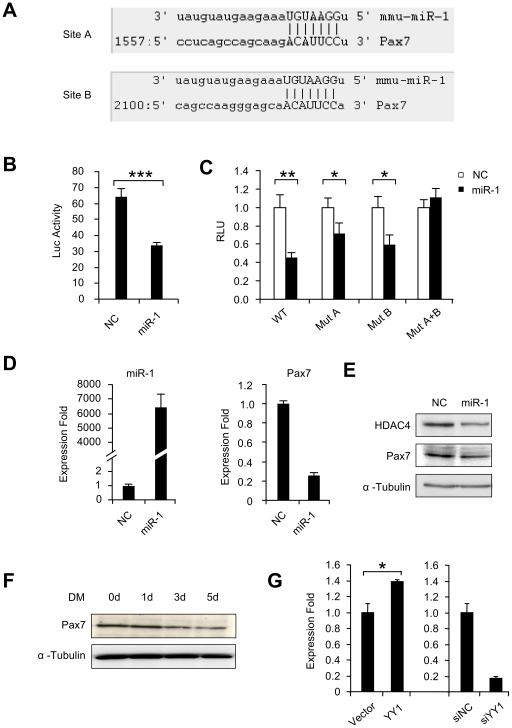
miR-1 targets Pax7 during C2C12 differentiation. (A) Predicted target sites, A and B, of miR-1 in the 3′UTR of mouse Pax7. (B) A luciferase reporter plasmid was generated by cloning a ∼800 bp region of Pax7 3′UTR encompassing both site A and B downstream of the luciferase (Luc) reporter gene. The reporter construct was then transfected into C2C12 cells with negative control (NC) or miR-1 oligos along with Renilla luciferase plasmid. Luciferase activities were determined at 48 h post-transfection and normalized to Renilla readings. The data represent the average of three independent experiments ± S.D. (C) A mutation was introduced in either site A (Mut A) or site B (Mut B) or both (Mut A+B). Their responses to miR-1 over-expression were tested as above. (D) C2C12 myoblasts were transfected with either NC or miR-1 oligos. Both miR-1 and Pax7 mRNAs levels were then measured 48 hr post-transfection. (E) HDAC4 or Pax7 proteins were probed in extracts from cells 48 hr after transfection. Blots were stripped and reprobed for α-Tubulin as the loading control. (F) Proteins extracted from C2C12 differentiated (DM) for 0 d, 1 d, 3 d and 5 d were used for Western blotting assay of Pax7. α-Tubulin was used as a loading control. (G) Left: C2C12 myoblasts were transfected with Vector or YY1 expression plasmid. Pax7 mRNA expression was then measured in extracts from cells 48 hr after transfection using GAPDH as normalization. Right: C2C12 myoblasts were transfected with siNC or siYY1 oligos. Pax7 mRNA expression was then measured in extracts from cells 48 hr after transfection using GAPDH as normalization. Expression folds are shown with respect to Vector or siNC control where Pax7 levels were set to a value of 1. Values are represented as mean ± S.D. The p value was determined by Student's T-test: *p<0.05, **p<0.01, ***p<0,001.

The functions of miR-1 in muscle development and muscle diseases have been the focus of intensive study. Deletion of miR-1-2 in mice caused dysregulation of cardiogenesis [32]; miR-1 was found to promote apoptosis of cardiomyocytes; recently, miR-1 was also demonstrated to exert a strong pro-myogenic influence on poorly differentiated Rhabdomyosarcoma cells [33]; in skeletal myogenesis, miR-1 is known to be pro-myogenic through targeting histone deacetlylase 4 (HDAC4) [15]. Given that miRNAs are known for their multi-targeting capability, we suspected that additional targets may exist downstream of miR-1 and under control of YY1-miR-1 axis. Interestingly, a search for putative miR-1 targets by multiple algorithm (TargetScan, PicTar, and miRanda) uncovered that Pax 7 could be a potential target with two putative target sites identified at 1557 and 2100 bp of its 3′UTR (Fig. 5A). To test whether miR-1 targets Pax7, a ∼800 bp fragment of Pax7 3′UTR encompassing these two sites were cloned into a pMIR-reporter vector. Co-transfection of the resultant reporter plasmid together with miR-1 into C2C12 cells caused a repression on luciferase activity (Fig. 5B), indicating that miR-1 indeed directly targets Pax7. Furthermore, mutation of either site A or site B restored the luciferease activity slightly but mutations of both sites led to a full restoration, indicating that these two sites may function synergistically (Fig. 5C). At the functional level, we predicted that miR-1 binding to the Pax7 3′UTR would lead to the repression on Pax7 expression. Consistent with this prediction, transfection of miR-1 oligos caused the decrease in both Pax7 mRNAs and proteins in addition to inhibiting the previously characterized target, HDAC4 (Fig. 5D–5E and Suppl. Fig. S4). In keeping with the above results, Pax7 expression level was down-regulated during the course of C2C12 differentiation concomitant to the gradual increase of miR-1 expression level (Fig. 5F). Furthermore, Pax7 was up-regulated in YY1 over-expressed C2C12 cells and down-regulated in siYY1 knock-down cells compared to Vector or siNC controls, suggesting that Pax7 is under control by YY1-miR-1 signaling (Fig. 5G). Taken together, these results implicate the existence of a functional YY1-miR-1-Pax7 circuit during myoblast differentiation.

### YY1 regulates miR-1 in cardiotoxin-induced muscle regeneration

Lastly, to address whether the regulation of YY1 on muscle miRs was relevant *in vivo*, we employed a Cardiotoxin (CTX)-induced muscle injury model. After injection with CTX, TA muscle displayed the typical degeneration-regeneration process: fiber degeneration and immune cell infiltration were immediately evident within 1 to 2 days; meanwhile, satellite cell started proliferating followed by activation and myogenic differentiation 3 to 4 days afterwards; newly formed fibers with centrally located nuclei were evident within 5 to 6 days; and muscle architecture was largely restored within 10 days (Suppl. Fig. S5).

**Figure 6:**
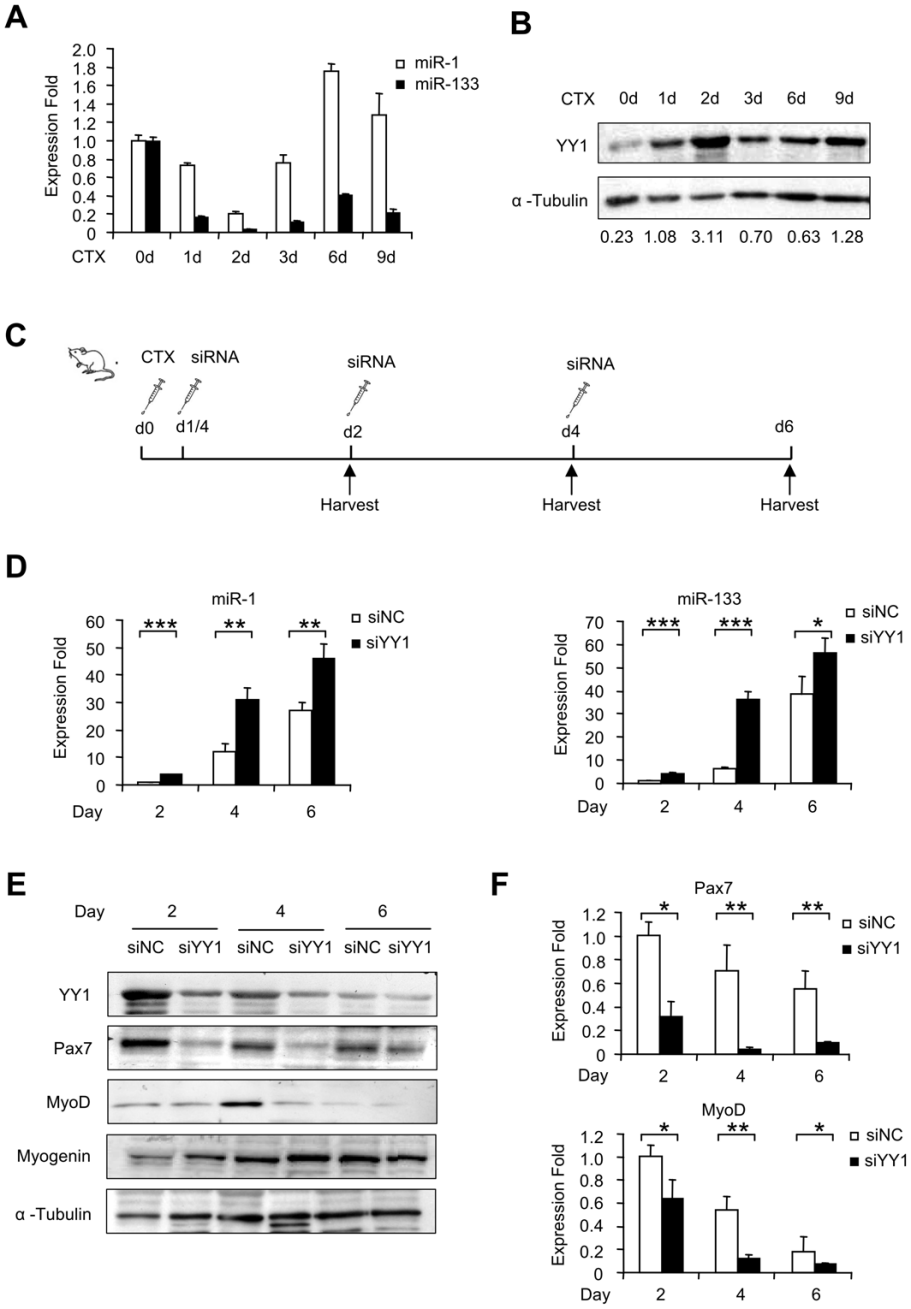
YY1 negatively regulates miR-1 during CTX-induced muscle regeneration. (A) Tibialis anterior (TA) muscles from six-week old C57/BL6 background mice were injected with 10 µM cardiotoxin (CTX). RNAs and proteins were then extracted from injected muscles at the indicated days post-injection, and qRT-PCR was performed to measure the expression of miR-1 and miR-133, normalized to U6. Expression folds are shown with respect to day 0 where miR-1 and miR-133 levels were set to a value of 1. Quantitative values are represented as means ± S.D. (B) YY1 expression was measured by Western blotting. α-Tubulin was used as a loading control. Numbers below indicates the quantification by densitometry. (C) TA muscles from 6 week C57/BL6 background mice were injected CTX at day 0, followed by injection with siNC (left leg) and siYY1 oligos (right leg) 6 hours later. And re-injection of siRNA oligos was performed every other day for two more times. The injected muscles were harvested at the indicated days. N = 6 for each group. (D) Expressions of miR-1 and miR-133 were detected by qRT-PCR in CTX/siRNA injected muscles at day 2, 4 and 6, normalized to U6. Expression folds are shown with respect to siNC where miR-1 and miR-133 levels were set to a value of 1. (E) Western blotting was performed to analyze the expression of YY1, Pax7, MyoD and Myogenin. α-Tubulin was used as a loading control. Data is representative of 6 mice. (F) Expression of Pax7 and MyoD RNA levels were also detected by qRT-PCR normalized with GAPDH. Expression folds are shown with respect to siNC where Pax7 and MyoD levels were set to a value of 1. Quantitative values are represented as mean ± S.D. The p value was determined by Student's T-test: *p<0.05, **p<0.01, ***p<0,001.

The expression profiles of miR-1 and miR-133 were examined by qRT-PCR analysis. As shown in Fig. 6A, for miR-1 expression, a sharp decrease of ∼5 fold in the first two days post-injury was observed, after which its expression level gradually increased to ∼2 fold of pre-injury level on day 6 but underwent a drop on day 9 during fiber maturation stage. The expression of miR-133 displayed a similar change but the decrease in degeneration stage was much stronger (∼10 fold) and the elevation in regeneration stage was lower compared to miR-1 level. This temporal expression pattern suggests that a dynamic change of miR-1 and 133 expressions occur during *in vivo* degeneration-regeneration process. The expression level of YY1 was subsequently examined by Western blotting analysis. To our expectation, it displayed an opposite trend as miR-1/133 expression: a marked increase during first two days followed by a decrease until day 6 and a bounce-back on day 9 (Fig. 6B). The above data suggested that an inverse correlation exists between the expression of miR-1/133 and YY1 during CTX-induced muscle regeneration. To ascertain the functional significance of this regulation, siYY1 or siNC oligos were injected into CTX-injured TA muscles following the administration scheme outlined in Fig. 6C. Six hours after CTX injection into TA muscles, siNC or siYY1 oligos formulated with Lipofectamine 2000 were administrated into left and right hind limbs, respectively. The injections were made every two days for a total of three times. Muscles were collected at day 2, 4 and 6 from treated muscles. Results from qRT-PCR analysis (Fig. 6D) revealed that the expressions of miR-1 and miR-133 were up-regulated in siYY1 injected muscles compared to siNC injected muscles at all three time points (day 2, 4 and 6) during muscle regeneration. A significant decrease of Pax 7 at both protein (Fig. 6E) and RNA levels (Fig. 6F) was observed at all time points examined, in support of the argument that Pax 7 is positively regulated by YY1 mediated through miR-1. An evident decrease of MyoD expression was also detected in siYY1 muscles compared to siNC samples (Fig. 6E and 6F). The decrease of Pax7 and MyoD implied that siYY1 knock-down may have inhibited satellite cell proliferation and activation. However, a significant increase of Myogenin expression was detected in siYY1 injected muscles (Fig. 6E), indicating that the myogenic differentiation was enhanced by siYY1 depletion. Given that a similar pro-myogenic effect was observed when miR-1 oligos were administrated into injured muscles [34], this suggests that YY1-miR-1 axis represents a functional regulatory circuit during CTX induced muscle regeneration.

### A feedback regulation of YY1 by miR-1

**Figure 7:**
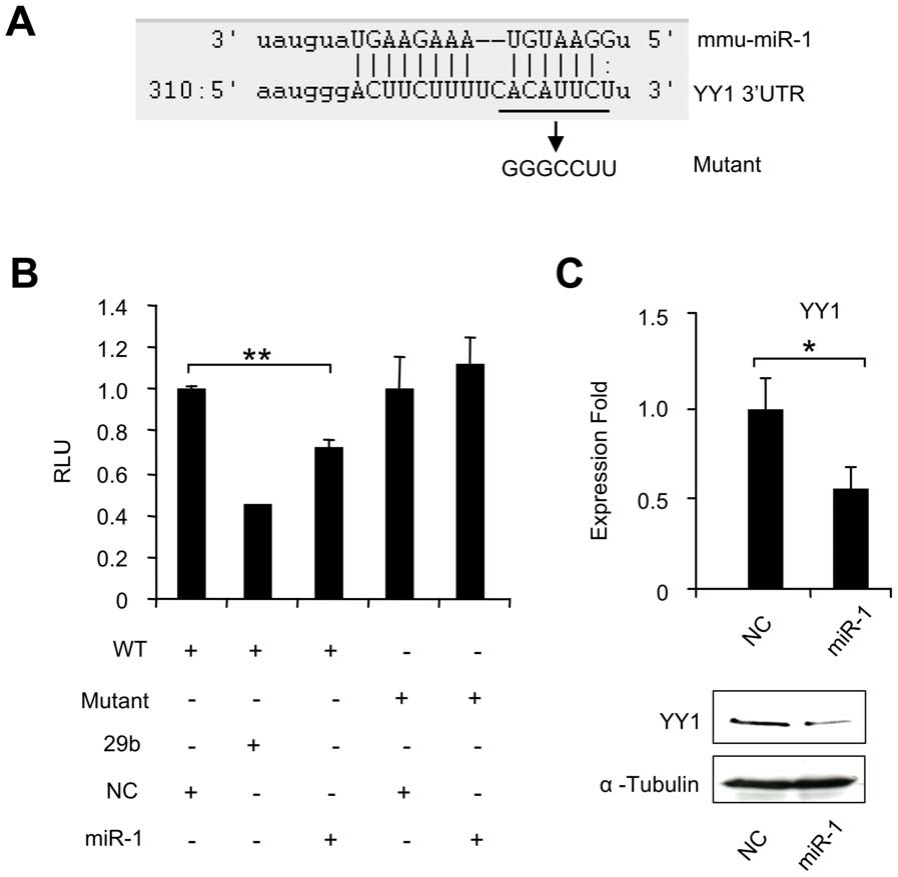
miR-1 inhibits YY1 expression through targeting its 3′UTR. (A) Predicted target site of miR-1 in the 3′UTR of mouse YY1. (B) A wild type (WT) luciferase reporter plasmid was generated by fusing a ∼500 bp fragment of the YY1 3′UTR encompassing the miR-1 binding site downstream of the luciferase (Luc) reporter gene. The mutant plasmid was generated by mutating the miR-1 binding site from ACAUUCU to GGGCCUU. WT or Mutant reporter construct was transfected into C2C12 cells with indicated miRNA oligos and Renilla luciferase reporter plasmid. Luciferase activities were determined at 48 h post-transfection and normalized to Renilla readings. Relative Luciferase Unit (RLU) is shown with respect to wild type and NC transfection where luciferase activities were set to a value of 1. The data represent the average of three independent experiments ± S.D. (C) Upper: C2C12 myoblasts were transfected with either NC or miR-1 oligos. Total RNAs were used to detect YY1 expression level with GAPDH as normalization. Expression folds are shown with respect to negative control where YY1 levels were set to a value of 1. Quantitative values are represented as mean ± S.D. Lower: YY1 protein was then probed in extracts from cells 48 hr after transfection. Blots were stripped and reprobed for α-Tubulin as the loading control. The p value was determined by Student's T-test: *p<0.05, **p<0.01, ***p<0.001.

Having gained insights into how YY1 regulates miR-1/133 family, we were intrigued to find out whether miR-1 can feedback on YY1 to regulate its expression since this type of feedback regulation is commonly existent between TFs and miRNAs. Indeed, a miR-1 binding site was predicted on the 3′ UTR of YY1 at 440 bp upstream of previously defined miR-29 binding site (Fig. 7A). To test whether it mediates YY1 targeting by miR-1, a reporter plasmid was generated by cloning this site into pMIR-reporter vector. Co-transfection of the resultant reporter plasmid with miR-1 oligos led to ∼30% repression on luciferase activity (Figure 7B). On the contrary, co-transfection of a negative control (NC) or an irrelevant miR-518 oligo did not show any repression. Furthermore, when the putative binding site was mutated, the repression on reporter activity was abolished. At the functional level, we predicted that miR-1 binding to the YY1 3′UTR would lead to repression of YY1 levels. Consistent with this prediction, transfection of miR-1 oligos caused decrease on both YY1 mRNA and protein levels (Fig. 7C). Collectively, our data support the existence of a feedback regulation on miR-1 by YY1.

## Discussion

**Figure 8:**
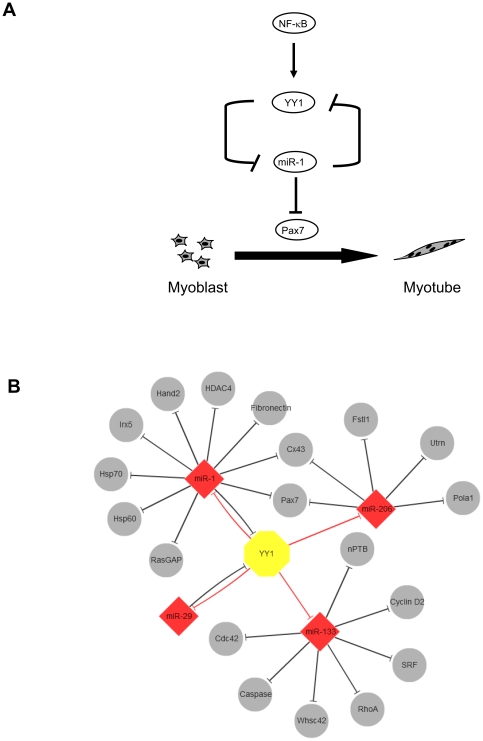
A model of NF-κB-YY1-miR-1-Pax7 circuit in skeletal myogenesis. The model depicts the role of the NF-κB-YY1-miR-1 regulatory circuit in myogenic differentiation. This circuit involves the constitutive activity of NF-κB in myoblasts that regulates YY1 expression which subsequently suppresses miR-1 expression epigenetically and maintains cells in an undifferentiated state. As differentiation is initiated, the down-regulation of the NF-κB-YY1 pathway leads to concomitant up-regulation of miR-1 that in turns further decreases YY1 as well as Pax7 levels to ensure proper differentiation into myotubes. (B) Network visualization depicting YY1-centered circuitry drawn by Cytoscape. Findings from the current studies demonstrate a repression of miR-1, miR-133 and miR-206 by YY1. Together with previous findings, these interactions constitute an YY1-miRNA regulatory network. YY1, Yellow octagon; miRNAs, red diamonds; Experimentally validated miRNA targets, grey circles; red blunted arrows, transcriptional repression; black blunted arrows, post-transcriptional repression.

This study was undertaken to investigate the potential interplay between transcription factor and epigenetic modifier, YY1, and miRNAs in skeletal myogenesis. Computational prediction of YY1-miRNA interaction led to the identification of many miRNAs as potential downstream targets of YY1. Subsequent functional study validated the existence of a novel YY1-miR-1/133 regulatory circuit during skeletal muscle differentiation both *in vitro* in C2C12 cells and *in vivo* in a CTX induced muscle regeneration model. Repression of muscle miR-1/133 family by YY1 was mediated through three YY1 binding sites located in multiple enhancer regions. Further investigation identified Pax7 as a downstream target of miR-1 thus forming a functional YY1-miR-1-Pax7 circuit which down-regulates Pax7 and promotes myogenic differentiation (Fig. 8A). Moreover, we demonstrated that YY1 is targeted by miR-1 through a negative feedback loop. Therefore, the current studies, together with previous findings, establish an YY1-centered functional network which integrates both transcriptional and post-transcriptional regulatory circuitries. As visualized by Cytoscape, miR-1, miR-133, miR-206 as well as miR-29 are all under transcriptional repression by YY1. These miRs, in turn, each down-regulates a set of protein coding genes at post-transcriptional level, among which includes YY1 itself as target of miR-1 and miR-29 (Fig. 8B).

The network of extensive regulatory interactions between transcriptional factors (TFs) and post-transcriptional regulators (miRs) is an interesting global feature and has been extensive focus of study [29], [30]. Our findings support the prevalence of reciprocal regulation between YY1 and miRNAs, i.e. YY1 regulates miR and the miR feeds-back on YY1. This kind of multi-level combinatorial interactions can ensure a gene to be tuned at a level of precision that is higher than what may be obtained by either mechanism alone.

Our findings also underscore the importance of YY1 as a chromatin modifier in skeletal myogenesis. Although emerging evidences implicate the inter-connection of miRNAs and the epigenetic machinery especially in various cancers [35], [36], studies of miRNA-epigenetics interplay is still scarce in the skeletal muscle research field [37]. Our findings support the notion that miRNome-epigenome interplay is a prevalent mechanism in skeletal muscle and also highlight the importance of histone-associated epigenetic control of muscle gene expression.

Despite increasing amounts of reports on the number of miRNAs involved in myogenesis, miR-1 and 133 families remain on the center stage [5]. The functional characterization of these muscle miRs has been critical in our understanding of miRNA-mediated muscle development. An intriguing feature of their function is their opposing roles in regulating myogenic differentiation. miR-1 is shown to promote myoblast differentiation while miR-133 promotes myoblast proliferation and inhibits myogenic differentiation [15]. The rapid induction of miR-133 during differentiation and its high expression level in muscle tissue seems to contradict its documented role as growth-stimulatory and anti-myogenic. We noticed that the level of miR-1 rose faster during both C2C12 myoblast differentiation and CTX induced muscle regeneration, leading us to speculate that the predominant increase in miR-1 overweighs that in miR-133, favoring the progression of myogenic differentiation. Our view is consistent with a recent report demonstrating that miR-1 and miR-133 produced opposing effects on apoptosis in cardiomyocytes despite the similar regulation [38]. It also indicates that it is critical to consider the combinatorial effects of miRs when interpreting their downstream output.

Although miR-1 and miR-133 are clustered on the same chromosomal loci and transcribed together as a single transcript, there seems to be a stronger regulation on miR-133 by YY1 than on miR-1 as depleting YY1 increased miR-133 to a much higher levels than that of miR-1 (Fig. 2F) and over-expressing YY1 also down-regulated miR-133 more than miR-1 (data not shown). We reasoned there may be two possibilities: first, it may be attributed to the fact that three copies of miR-133 are under regulation by YY1 whereas only two copies of miR-1 are subjected to YY1 regulation (Fig. 3A–C). Second, in addition to regulating the transcription of primary miR-1/133 transcripts, YY1 may also exert regulation at a later stage of miR-1/133 biogenesis, resulting in different rates of miR-1 and miR-133 production. Thus, the regulation by YY1 may exert a master control of the ratio of miR-1 and 133 which is critical for their net effect on myogenesis. It should be noted that our study only provided indirect evidence for the above notion and more rigorous experimentation is required to test our hypothesis. In addition, our studies focused on the regulation of YY1 on miR-1 and its downstream event; it will be interesting to explore how YY1 regulated miR-133 affect satellite cell proliferation and differentiation processes.

In this study, we identified two new targets of miR-1, Pax7 and YY1, in addition to the previously known HDAC4. During the preparation of this manuscript, Chen et al recently demonstrated that miR-1 and miR-206 regulate skeletal muscle satellite cell proliferation and differentiation by repressing Pax7 using primary satellite cell culture [39]. Our results from using C2C12 cells reinforced the view that Pax7 is crucial for satellite cell proliferation and needs to be down-regulated for the cells to transit into myogenic differentiation. The YY1-miR-1-Pax7 axis represents an important circuit that regulates transition from proliferation to differentiation. In addition to its anti-differentiation function, YY1, through its regulation on miR-1-Pax7, may play a crucial role in regulating satellite cell proliferation. Indeed, YY1 expression has sharply increased after CTX injury (Fig. 6). And knocking-down of YY1 down-regulated Pax7 in CTX injured muscles. Our results, thus, reinforce the idea that miRNA exerts their function through multiple targets to provide robust regulation during myogenic differentiation.

Others and our findings suggest that miR-1 not only functions during myogenic differentiation but also participates in satellite cell activation, proliferation and possibly self-renewal. In addition, Nakasa T. also showed that administration of miR-1 together with 133 and 206 into injured muscles reduced fibrosis [34], suggesting a role for miR-1 in anti-fibrotic event with unknown mechanism. It will be interesting to dissect the downstream regulatory targets mediating these functions of miR-1 and to elucidate the significance of YY1-miR-1 regulation in various events.

Finally, other than miR-1 and 133 families, a number of miRNAs could be regulated by YY1 according to our results (Table 1 and Suppl. Table S2), strengthening the idea that regulatory circuitry involving miRNAs and TFs are prevalent mechanisms of gene expression. Characterization of their interplay with YY1 and downstream targets will enhance our understanding on the global architecture of the combined transcriptional/post-transcriptional regulatory network. In addition, our current studies demonstrated that bioinformatics pipeline combining computational prediction with expression data can be a powerful tool for constructing TF-miRNA regulatory network, particularly for those TFs on which the genome-wide mapped targets by experimental methods such as ChIP coupled with high throughput sequencing (ChIP-seq) are not available yet. This kind of network analysis will provide insights into biological systems that cannot be obtained in single TF or miRNA studies.

## Materials and Methods

### Cell

Mouse C2C12 myoblasts were obtained from ATCC and cultured in DMEM supplemented with 10% FBS, 2 mM L-glutamine, 100 U/ml penicillin, and 100 µg of Streptomycin at 37°C in 5% CO_2_. For myogenic differentiation experiments, cells were seeded in 60 mm or 100 mm plates and when 90% confluent they were shifted to DMEM without FBS containing 2% horse serum.

### Primary myoblast isolation and culture

Primary myoblasts were isolated from approximately one week old C57/BL6 muscles as previously described. Briefly, total hind limb muscles (3 to 6 mice per group) were digested with type IV collagenase (Invitrogen, 5 mg/ml) and dispase II (Invitrogen, 1.4 mg/ml) for 0.5 hr, and cell suspensions were filtered through 40 µM and 70 µM filters and then pre-plated for an hour. Non-adherent cells were centrifuged and cultured on Gelatin-coated plates (Iwaki) in F10 medium (Invitrogen) supplemented with 20% FBS and Basic Fibroblast Growth Factor (Invitrogen, 25 ng/ml). After removing most fibroblast by pre-plating, primary myoblast cells are cultured in F10/DMEM medium supplemented with 20% FBS and Basic Fibroblast Growth Factor (Invitrogen).

### Transfection and infection

Transient transfection with miRNA precursor oligos or siRNA oligos was performed in 60 mm or 100 mm dishes with Lipofectamine 2000 as suggested by the manufacturer (Invitrogen). For luciferase experiments, C2C12 and primary mouse skeletal muscle myoblasts were transfected in 12-well plates. Cell extracts were prepared and luciferase activity was monitored using Dual Luciferase kit (Promega, E1910).

### Oligonucleotides

Precursor miRNA oligos were obtained from Ambion. siRNA oligos against mouse YY1 were obtained from Santa Cruz technologies. In each case 50 µM oligos were used for transient transfections. The sequences of oligonucleotides used for RT-PCR, ChIP-PCR and cloning are included in Supplemental Material (Suppl. table S5).

### DNA constructs

To construct E1, E2 and E4 luciferase reporter plasmids, the enhancer fragments E1: chr18:10,787,547–10,788,045 (486 bp), E2: chr18:10,784,080–10,784,701 (622 bp), or E4: chr1:20,667,759–20,668,359 (600 bp), were PCR amplified from mouse genomic DNA and cloned between a Kpn1 and Nhe1 site of a PGL2 vector (Promega), upstream of the luciferase gene; E3 luciferase reporter was generated as above by cloning 1150 bp (chr2:180,129,142–180,130,290) of PCR fragment between Kpn1 and Xho1 site of PGL2 vector. Mutant reporter plasmids were generated by deleting YY1 binding site A or mutating binding site B from ATGG to GTCC. YY1 expression plasmid and YY1-3′UTR luciferase reporter plasmid were described before (Wang et al., 2008). A mutant YY1-3′UTR plasmid was generated by changing miR-1 binding site from ACATTC to GGGCCT. To construct Pax7-3′UTR reporter plasmid, an 805 bp (chr4:139,294,480–139,295,283) fragment encompassing binding site A and site B was cloned into pMIR-report vector (ABI) between SpeI and HindIII sites. The mutant Pax7-3′UTR plasmids were generated by deleting each of the miR-1 binding seed region ACATTCC.

### RT-PCR and Real-time RT-PCR

Total RNAs from cells were extracted using TRIzol reagent (Invitrogen). Expression of mature miRNAs was determined using the miRNA-specific Taqman microRNA assay kit (Applied Biosystem) in a 7900HT system (Applied Biosystem). U6 was used for normalization. Expression of mRNA analysis was performed as described using GAPDH for normalization [19].

### Immunoblotting and Immunostaining

Total cell extracts were prepared and used for analysis of protein expression as previously described [19]. The following dilutions were used for each antibody: YY1 (mouse monoclonal from Santa Cruz Biotechnology, Cat# SC-7341; 1∶1000), MyHC (mouse monoclonal from Sigma, Cat# M4276; 1∶1,000), HDAC4(rabbit polyclonal from Santa Cruz Biotechnology, Cat# SC-11418; 1∶1000), Pax7 (mouse monoclonal from DSHB, 1∶1000), α-Actin (goat polyclonal from Santa Cruz Biotechnology, Cat# SC-1615; 1∶1000), Troponin (mouse monoclonal from Sigma, Cat# T6277; 1∶1000), MyoD (rabbit polyclonal from Santa Cruz Biotechnology, Cat# SC-760; 1∶1000), Myogenin (rabbit polyclonal from Santa Cruz Biotechnology, Cat# SC-576; 1∶1000), α-Tubulin (mouse monoclonal from Sigma, Cat# T5168; 1∶5000), GAPDH (mouse monoclonal from Santa Cruz Biotechnology, Cat# SC-137179; 1∶5000). Immunofluorescence of C2C12 cells was performed using a MyHC monoclonal antibody (mouse monoclonal from Sigma, Cat# M4276) at 1∶500 dilution.

### ChIP assays

ChIP assays were performed as previously described (Tong Ihn Lee, 2006) using 5 µg of antibodies against YY1 (rabbit polyclonal from Santa Cruz Biotechnology, Cat# SC-1703), Ezh2 (mouse monoclonal from Cell Signaling, Cat# AC22), trimethyl-histone H3-K27 (rabbit polyclonal from Milipore, Cat# 07-449), or without any antibody as a negative control. Genomic DNA pellets were resuspended in 10 µl of water. PCR was performed with 1 µl of immunoprecipitated material and products were analyzed on an agarose gel visualized by a GelDoc documentation system (Bio-Rad Laboratories).

### Animal studies

Mice used in this study were housed in the animal facilities of the Chinese University of Hong Kong under conventional conditions with constant temperature and humidity and fed a standard diet. Animal experimentation was approved by the CUHK Animal Experimentation Ethics Committee (Ref No. 10/027/MIS). Postnatal muscles were obtained from C57/BL6 mice, and RNAs were extracted for real-time RT-PCR analysis. For Cardiotoxin studies, approximately six-week old C57/BL6 mice were injected with 60 µl of Cardiotoxin at 10 µg/ml into the tibialis anterior muscle. Muscles were harvested at designated times, and RNAs were extracted for real-time RT-PCR analysis, and proteins were extracted for Western analysis. Injections of siRNA oligos were performed six hours after Cardiotoxin injection and re-injected every two days for a total of three times. Oligos were prepared by pre-incubating 5 µM of siRNA oligos with Lipofectamine 2000 for 15 minutes and injections were made in a final volume of 60 µl in OPTI-EM (GIBCO). Mdx (C57BL/10 ScSn DMDmdx) mice were purchased from the Jackson Laboratory. Mice were housed in the animal facilities of the Chinese University of Hong Kong under conventional conditions with constant temperature and humidity and fed a standard diet. Animal experimentation was approved by the CUHK Animal Ethics Committee.

### Computational prediction pipeline for TF binding sites identification

miRNA promoter annotation was retrieved from Marson et al [22]. A promoter prediction program EP3 [23] was used to further refine the miRNA promoter regions using 200 bp window. The refined promoter regions were extended by 15 kbp on both sides and subjected to YY1 binding sites search. In order to predict YY1 binding sites, four YY1 position weight matrices (PWMs) were retrieved from Transfac database [24] and used to scan the promoter regions defined above using STORM program [25] by selecting the top 500 YY1 binding sites among all the predictions. Prediction of miRNA target was performed using three publicly available algorithms: TargetScan (http://www.targetscan.org/), miRanda (http://www.microrna.org/) and PicTar (http://pictar.bio.nyu.edu/). Network visualization was carried out using Cytoscape. (http://cytoscapeweb.cytoscape.org/).

### miRNA gene expression by microarray

RNAs were extracted from C2C12 myoblasts or myotubes by Trizol and used for genome-wide miRNA profiling using a microarray platform developed by the Ohio State University, USA (microRNACHIPv4, http://www.osuccc.osu.edu/microarray/).Each time point was represented by two biological replicates. For each sample, 1 µg of total RNAs were labeled and hybridized to the microarray. Microarrays were scanned on Axon 4000B Scanner (Molecular Devices). Data were subsequently analyzed by a software package limma [40], [41] from R for array normalization. After normalization, the miRNA probes were ranked by RankProd [42] based on their normalized intensity. The heatmap of the ranked miRNA probes was generated using Java Treeview [43]. All microarray data presented in this manuscript are in accordance with Minimum Information About a Microarray Experiment (MIAME) guidelines and have been deposited in the National Center for Biotechnology Information (NCBI) GEO data base (accession number: GSE32238). In order to visualize if YY1 target miRNA genes are enriched in the differentially expressed miRNAs, the frequency of miRNA probes belonging to the miRNA genes that predicted as YY1 targets was calculated within each 300-probe sliding window with 1 probe increment each time from the probe sets that are ranked by RankProd.

### Statistical analysis

Data were represented as means± standard deviation (S.D.). Statistical significance between two groups was determined by Student's t test. A P value of less than 0.05 was considered statistically significant.

## Supporting Information

Figure S1 **YY1 was successfully knocked down by siRNA oligos in C2C12 cells.** C2C12 myoblasts were transfected with 50 nM of negative control (siNC) or YY1 siRNA (siYY1) oligos. 48 hr post-transfection, cells were collected for Western blotting analysis of YY1 protein expression using an YY1 antibody. α-Tubulin was used as a loading control.
(PDF)

Figure S2 **NFκB suppresses miR-1 and miR-133 expression through YY1.** (A) C2C12 cells were treated with 10 ng/ml of TNFα. miR-1 and miR-133 expression was then measured by qRT-PCR normalized to U6. Expression folds are shown with respect to TNF treated cells where miR-1 or miR-133 levels were set to a value of 1. (B) Expression of miR-1 and miR-133 was measured in C2C12 myoblasts stably expressing Vector or the IκBα-SR transgene. Expression folds are shown with respect to vector cells, which were set to a value of 1. Quantitative values are represented as mean ± S.D.
(PDF)

Figure S3 **Activation of E1 luciferase reporter by MyoD and MEF2.** (A and B) C2C12 cells were transfected with MyoD or MEF expressing plasmids along with E1 luciferase reporter plasmid and Renilla reporter plasmid. Luciferase activities were determined at 48 h post-transfection and normalized to Renilla readings. The data represent the average of three independent experiments ± S.D.
(PDF)

Figure S4 **Pax7 protein is down-regulated by miR-1 over-expression.** C2C12 myoblasts were transfected with either NC or miR-1 oligos. Pax7 proteins were probed in extracts from cells 48 hr after transfection. Blots were stripped and reprobed for GAPDH as the loading control.
(PDF)

Figure S5 **CTX induced muscle degeneration and regeneration.** Cardiotoxin was injected into Tibialis anterior muscles of C57/BL6 mice. Muscles were harvested at designated times. H&E staining was performed on cryosections of muscles.
(PDF)

Table S1 **Predicted promoter regions for mouse miRNA genes.** The promoter score was extracted from Marson *et al.* [22]. Four YY1 positional weight matrices from Transfac database, YY1_01, YY1_02, YY1_Q6, and YY1_Q6_02, were used to scan for YY1 binding motif on miRNA promoters. The motif score was calculate by Storm [25]. Relative motif position refers to the distance to promoter start position.
(PDF)

Table S2 **Up-regulated miRNAs during myoblasts differentiation into myotubes as revealed by miRNA microarray profiling.** C2C12 myoblasts were grown in growth medium (GM) or differentiated in differentiation medium (DM) for 1 or 3 days. Total RNAs were extracted and subjected to expression profiling using a microarray platform. A total of 77 miRNAs were found to be up-regulated in DM 3 d as compared to GM.
(PDF)

Table S3 **Down-regulated miRNAs during myoblasts differentiation into myotubes as revealed by miRNA microarray profiling.** A total of 68 miRNAs were found to be down-regulated with a fold change more than 2.
(PDF)

Table S4 **Computational prediction reveals the prevalence of YY1 binding sites on promoters of down-regulated miRNAs.** The 68 down-regulated miRNAs were run through the YY1 binding site search pipeline. A total of 27 miRNAs were found to contain at least one YY1 binding site on their promoters.
(PDF)

Table S5 **Oligonucleotides used for RT-PCR, ChIP-PCR and cloning.**
(PDF)

## References

[1] Bartel DP (2004). MicroRNAs: genomics, biogenesis, mechanism, and function.. Cell.

[2] Liu N, Olson EN (2010). MicroRNA regulatory networks in cardiovascular development.. Dev Cell.

[3] Ivey KN, Srivastava D (2010). MicroRNAs as regulators of differentiation and cell fate decisions.. Cell Stem Cell.

[4] Cordes KR, Srivastava D, Ivey KN (2010). MicroRNAs in cardiac development.. Pediatr Cardiol.

[5] Townley-Tilson WH, Callis TE, Wang D (2010). MicroRNAs 1, 133, and 206: critical factors of skeletal and cardiac muscle development, function, and disease.. Int J Biochem Cell Biol.

[6] Guller I, Russell AP (2010). MicroRNAs in skeletal muscle: their role and regulation in development, disease and function.. J Physiol.

[7] Buckingham M (2006). Myogenic progenitor cells and skeletal myogenesis in vertebrates.. Curr Opin Genet Dev.

[8] Sabourin LA, Rudnicki MA (2000). The molecular regulation of myogenesis.. Clin Genet.

[9] Olguin HC, Olwin BB (2004). Pax-7 up-regulation inhibits myogenesis and cell cycle progression in satellite cells: a potential mechanism for self-renewal.. Dev Biol.

[10] Rao PK, Kumar RM, Farkhondeh M, Baskerville S, Lodish HF (2006). Myogenic factors that regulate expression of muscle-specific microRNAs.. Proc Natl Acad Sci U S A.

[11] Zhao Y, Samal E, Srivastava D (2005). Serum response factor regulates a muscle-specific microRNA that targets Hand2 during cardiogenesis.. Nature.

[12] Anderson C, Catoe H, Werner R (2006). MIR-206 regulates connexin43 expression during skeletal muscle development.. Nucleic Acids Res.

[13] Kim HK, Lee YS, Sivaprasad U, Malhotra A, Dutta A (2006). Muscle-specific microRNA miR-206 promotes muscle differentiation.. J Cell Biol.

[14] Rosenberg MI, Georges SA, Asawachaicharn A, Analau E, Tapscott SJ (2006). MyoD inhibits Fstl1 and Utrn expression by inducing transcription of miR-206.. J Cell Biol.

[15] Chen JF, Mandel EM, Thomson JM, Wu Q, Callis TE (2006). The role of microRNA-1 and microRNA-133 in skeletal muscle proliferation and differentiation.. Nat Genet.

[16] Juan AH, Sartorelli V (2010). MicroRNA-214 and polycomb group proteins: A regulatory circuit controlling differentiation and cell fate decisions.. Cell Cycle.

[17] Griffiths-Jones S, Saini HK, van Dongen S, Enright AJ (2008). miRBase: tools for microRNA genomics.. Nucleic Acids Res.

[18] Wang H, Garzon R, Sun H, Ladner KJ, Singh R (2008). NF-kappaB-YY1-miR-29 regulatory circuitry in skeletal myogenesis and rhabdomyosarcoma.. Cancer Cell.

[19] Wang H, Hertlein E, Bakkar N, Sun H, Acharyya S (2007). NF-kappaB regulation of YY1 inhibits skeletal myogenesis through transcriptional silencing of myofibrillar genes.. Mol Cell Biol.

[20] Caretti G, Di Padova M, Micales B, Lyons GE, Sartorelli V (2004). The Polycomb Ezh2 methyltransferase regulates muscle gene expression and skeletal muscle differentiation.. Genes Dev.

[21] Hyde-DeRuyscher RP, Jennings E, Shenk T (1995). DNA binding sites for the transcriptional activator/repressor YY1.. Nucleic Acids Res.

[22] Marson A, Levine SS, Cole MF, Frampton GM, Brambrink T (2008). Connecting microRNA genes to the core transcriptional regulatory circuitry of embryonic stem cells.. Cell.

[23] Abeel T, Saeys Y, Bonnet E, Rouze P, Van de Peer Y (2008). Generic eukaryotic core promoter prediction using structural features of DNA.. Genome Res.

[24] Matys V, Kel-Margoulis OV, Fricke E, Liebich I, Land S (2006). TRANSFAC and its module TRANSCompel: transcriptional gene regulation in eukaryotes.. Nucleic Acids Res.

[25] Schones DE, Smith AD, Zhang MQ (2007). Statistical significance of cis-regulatory modules.. BMC Bioinformatics.

[26] Liu CG, Calin GA, Volinia S, Croce CM (2008). MicroRNA expression profiling using microarrays.. Nat Protoc.

[27] Wong CF, Tellam RL (2008). MicroRNA-26a targets the histone methyltransferase Enhancer of Zeste homolog 2 during myogenesis.. J Biol Chem.

[28] Fei T, Xia K, Li Z, Zhou B, Zhu S (2010). Genome-wide mapping of SMAD target genes reveals the role of BMP signaling in embryonic stem cell fate determination.. Genome Res.

[29] Martinez NJ, Walhout AJ (2009). The interplay between transcription factors and microRNAs in genome-scale regulatory networks.. Bioessays.

[30] Shalgi R, Lieber D, Oren M, Pilpel Y (2007). Global and local architecture of the mammalian microRNA-transcription factor regulatory network.. PLoS Comput Biol.

[31] Liu N, Williams AH, Kim Y, McAnally J, Bezprozvannaya S (2007). An intragenic MEF2-dependent enhancer directs muscle-specific expression of microRNAs 1 and 133.. Proc Natl Acad Sci U S A.

[32] Zhao Y, Ransom JF, Li A, Vedantham V, von Drehle M (2007). Dysregulation of cardiogenesis, cardiac conduction, and cell cycle in mice lacking miRNA-1-2.. Cell.

[33] Rao PK, Missiaglia E, Shields L, Hyde G, Yuan B (2010). Distinct roles for miR-1 and miR-133a in the proliferation and differentiation of rhabdomyosarcoma cells.. FASEB J.

[34] Nakasa T, Ishikawa M, Shi M, Shibuya H, Adachi N (2010). Acceleration of muscle regeneration by local injection of muscle-specific microRNAs in rat skeletal muscle injury model.. J Cell Mol Med.

[35] Fabbri M, Calin GA (2010). Epigenetics and miRNAs in human cancer.. Adv Genet.

[36] Iorio MV, Ferracin M, Liu CG, Veronese A, Spizzo R (2005). MicroRNA gene expression deregulation in human breast cancer.. Cancer Res.

[37] Saccone V, Puri PL (2010). Epigenetic regulation of skeletal myogenesis.. Organogenesis.

[38] Xu C, Lu Y, Pan Z, Chu W, Luo X (2007). The muscle-specific microRNAs miR-1 and miR-133 produce opposing effects on apoptosis by targeting HSP60, HSP70 and caspase-9 in cardiomyocytes.. J Cell Sci.

[39] Chen JF, Tao Y, Li J, Deng Z, Yan Z (2010). microRNA-1 and microRNA-206 regulate skeletal muscle satellite cell proliferation and differentiation by repressing Pax7.. J Cell Biol.

[40] Ritchie ME, Silver J, Oshlack A, Holmes M, Diyagama D (2007). A comparison of background correction methods for two-colour microarrays.. Bioinformatics.

[41] Smyth GK, Speed T (2003). Normalization of cDNA microarray data.. Methods.

[42] Breitling R, Armengaud P, Amtmann A, Herzyk P (2004). Rank products: a simple, yet powerful, new method to detect differentially regulated genes in replicated microarray experiments.. FEBS Lett.

[43] Saldanha AJ (2004). Java Treeview–extensible visualization of microarray data.. Bioinformatics.

