# Supplementary figures and images for: A Novel YY1-miR-1 Regulatory Circuit in Skeletal Myogenesis Revealed by Genome-Wide Prediction of YY1-miRNA Network
Source: PLoS One. 2012 Feb 1;7(2):e27596. doi: 10.1371/journal.pone.0027596 (PMC3271076; doi:10.1371/journal.pone.0027596)

**Supplemental Figure S1**  
**Lu et al.**

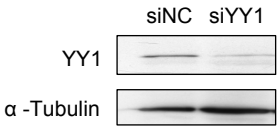

Supplement: Figure S1 — YY1 was successfully knocked down by siRNA oligos in C2C12 cells. C2C12 myoblasts were transfected with 50 nM of negative control (siNC) or YY1 siRNA (siYY1) oligos. 48 hr post-transfection, cells were collected for Western blotting analysis of YY1 protein expression using an YY1 antibody. α-Tubulin was used as a loading control. (PDF) [file pone.0027596.s001.pdf]

Supplemental Figure S2  
Lu et al.

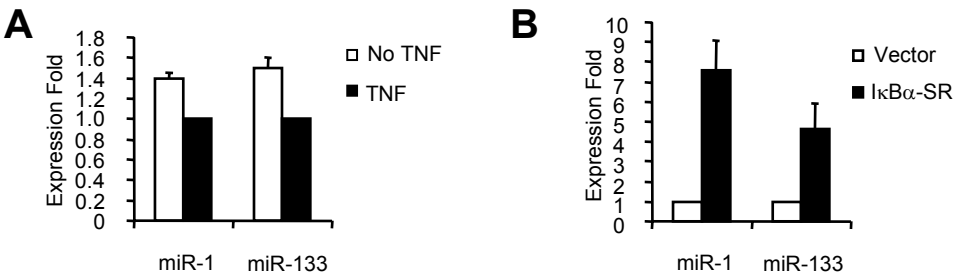

Supplement: Figure S2 — NFκB suppresses miR-1 and miR-133 expression through YY1. (A) C2C12 cells were treated with 10 ng/ml of TNFα. miR-1 and miR-133 expression was then measured by qRT-PCR normalized to U6. Expression folds are shown with respect to TNF treated cells where miR-1 or miR-133 levels were set to a value of 1. (B) Expression of miR-1 and miR-133 was measured in C2C12 myoblasts stably expressing Vector or the IκBα-SR transgene. Expression folds are shown with respect to vector cells, which were set to a value of 1. Quantitative values are represented as mean ± S.D. (PDF) [file pone.0027596.s002.pdf]

Supplemental Figure S3  
Lu et al.

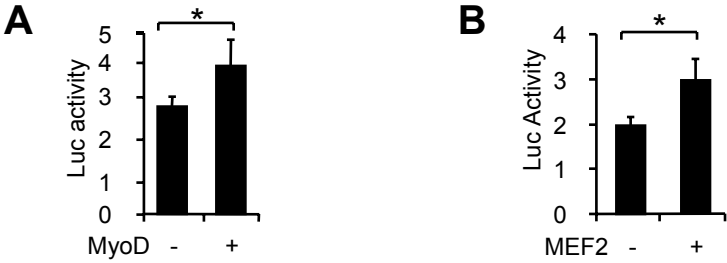

Supplement: Figure S3 — Activation of E1 luciferase reporter by MyoD and MEF2. (A and B) C2C12 cells were transfected with MyoD or MEF expressing plasmids along with E1 luciferase reporter plasmid and Renilla reporter plasmid. Luciferase activities were determined at 48 h post-transfection and normalized to Renilla readings. The data represent the average of three independent experiments ± S.D. (PDF) [file pone.0027596.s003.pdf]

Supplemental Figure S4  
Lu et al.

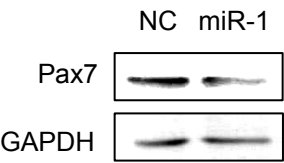

Supplement: Figure S4 — Pax7 protein is down-regulated by miR-1 over-expression. C2C12 myoblasts were transfected with either NC or miR-1 oligos. Pax7 proteins were probed in extracts from cells 48 hr after transfection. Blots were stripped and reprobed for GAPDH as the loading control. (PDF) [file pone.0027596.s004.pdf]

Supplemental Figure S5  
Lu et al.

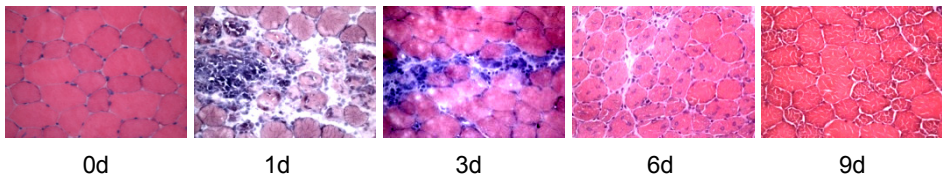

Supplement: Figure S5 — CTX induced muscle degeneration and regeneration. Cardiotoxin was injected into Tibialis anterior muscles of C57/BL6 mice. Muscles were harvested at designated times. H&E staining was performed on cryosections of muscles. (PDF) [file pone.0027596.s005.pdf]
