# Supplementary material for: A Novel YY1-miR-1 Regulatory Circuit in Skeletal Myogenesis Revealed by Genome-Wide Prediction of YY1-miRNA Network
Source: PLoS One. 2012 Feb 1;7(2):e27596. doi: 10.1371/journal.pone.0027596 (PMC3271076; doi:10.1371/journal.pone.0027596)
Supplement: Table S1 — Predicted promoter regions for mouse miRNA genes. The promoter score was extracted from Marson et al. [22]. Four YY1 positional weight matrices from Transfac database, YY1_01, YY1_02, YY1_Q6, and YY1_Q6_02, were used to scan for YY1 binding motif on miRNA promoters. The motif score was calculate by Storm [25]. Relative motif position refers to the distance to promoter start position. (PDF) [file pone.0027596.s006.pdf]

Suppl. Table S1: Predicted promoter regions for mouse miRNAs.
Position weight matrix,YY1_01, was used to scan for YY1 motif.

Motif Sequence	miRNA ID	Promoter Score	Promoter Chr	Promoter Start	Promoter End	Relative Motif Location	Strand	YY1 Motif Search Score
TCTGACCATCTTGGCAT	mmu-let-7a-1	10	chr13	48551775	48553275	92	-	11.3456
TAGCACCATTTATATAG	mmu-let-7a-1	10	chr13	48551775	48553275	947	-	11.2131
TGTGTTCATTTTTCTAG	mmu-let-7a-1	10	chr13	48551775	48553275	179	-	9.71975
TCTCTCCATCCTGGGAA	mmu-let-7a-1	10	chr13	48551775	48553275	1128	-	9.36248
CTTAGCCATTTTTCCAG	mmu-let-7a-1	10	chr13	48551775	48553275	566	-	8.91246
TACATCCATATTCTCTT	mmu-let-7b	0	chr15	85515125	85516200	778	-	9.31552
TGTCCCCATTTTGCAGA	mmu-let-7b	0	chr15	85515125	85516200	901	-	9.06899
AGCCACCATGTATGTAT	mmu-let-7b	0	chr15	85515125	85516200	658	-	9.03517
TACATCCATATTCTCTT	mmu-let-7c-2	0	chr15	85515125	85516200	778	-	9.31552
TGTCCCCATTTTGCAGA	mmu-let-7c-2	0	chr15	85515125	85516200	901	-	9.06899
AGCCACCATGTATGTAT	mmu-let-7c-2	0	chr15	85515125	85516200	658	-	9.03517
TCTGACCATCTTGGCAT	mmu-let-7d	0	chr13	48551775	48553275	92	-	11.3456
TAGCACCATTTATATAG	mmu-let-7d	0	chr13	48551775	48553275	947	-	11.2131
TGTGTTCATTTTTCTAG	mmu-let-7d	0	chr13	48551775	48553275	179	-	9.71975
TCTCTCCATCCTGGGAA	mmu-let-7d	0	chr13	48551775	48553275	1128	-	9.36248
CTTAGCCATTTTTCCAG	mmu-let-7d	0	chr13	48551775	48553275	566	-	8.91246
TCTGACCATCTTGGCAT	mmu-let-7f-1	10	chr13	48551775	48553275	92	-	11.3456
TAGCACCATTTATATAG	mmu-let-7f-1	10	chr13	48551775	48553275	947	-	11.2131
TGTGTTCATTTTTCTAG	mmu-let-7f-1	10	chr13	48551775	48553275	179	-	9.71975
TCTCTCCATCCTGGGAA	mmu-let-7f-1	10	chr13	48551775	48553275	1128	-	9.36248
CTTAGCCATTTTTCCAG	mmu-let-7f-1	10	chr13	48551775	48553275	566	-	8.91246
AAAGATCATTTTTGGTT	mmu-let-7i	10	chr10	122385250	1.22E+08	190	-	10.5121
GCAGTTCATTTTTGAGG	mmu-let-7i	10	chr10	122385250	1.22E+08	1103	+	9.90679
CGCCGCCATCTTTACAG	mmu-let-7i	10	chr10	122385250	1.22E+08	4146	-	9.88756
CGCCGCCATCTTACATC	mmu-let-7i	10	chr10	122385250	1.22E+08	3968	+	8.91008
CACAGCCATATATTCAC	mmu-mir-106a	0	chrX	48985925	48991150	711	+	9.75653
GAAACCCATTTTTTTTC	mmu-mir-106a	0	chrX	48985925	48991150	17	+	9.72013
GGTATCCATCTGTAAAC	mmu-mir-106a	0	chrX	48985925	48991150	395	-	8.92002
GGGTTCCATTTCCTAAT	mmu-mir-106a	0	chrX	48985925	48991150	3369	-	8.83589
TCTCACCATACTGAAAG	mmu-mir-1-2	0	chr18	10848725	10849950	18	-	10.3378
GATCACCATGGTCAGTG	mmu-mir-122	0	chr18	65208050	65210300	1510	+	9.15524
GAAGGCCATTTCTGTTT	mmu-mir-124-1	0	chr14	63540450	63546275	220	-	9.22365

CCACTCCATTGACCGAA	mmu-mir-124-1	0	chr14	63540450	63546275	1194	+	8.79815
GATTTCCATCTTTGGAG	mmu-mir-125b-2	10	chr16	77525525	77528275	1763	+	12.5662
TAAAGCCATATTCAGTT	mmu-mir-125b-2	10	chr16	77525525	77528275	1908	+	10.3376
CGTCTTCATTTTTGCAC	mmu-mir-127	-10	chr12	109987400	1.1E+08	1539	-	11.4637
GGACCCCATTTTCACTA	mmu-mir-127	-10	chr12	109987400	1.1E+08	633	+	9.67413
TATTTCCATTTCTAATT	mmu-mir-129-2	10	chr2	94041175	94045400	1739	+	9.91911
CAGGTCCATTCTGGGAT	mmu-mir-129-2	10	chr2	94041175	94045400	2580	-	9.70695
CAACTCCATGTAGGGGA	mmu-mir-129-2	10	chr2	94041175	94045400	3414	+	9.55498
GGCCTCCATTTTTCCTG	mmu-mir-130a	0	chr2	84542025	84546150	887	-	11.3653
TCACTCCATTCAAGAAT	mmu-mir-130a	0	chr2	84542025	84546150	2577	-	9.8334
GCCGTCCATCTTGAATA	mmu-mir-130a	0	chr2	84542025	84546150	2554	+	9.46515
GGCCACCATCTTCCGGC	mmu-mir-130a	0	chr2	84542025	84546150	1462	+	9.09865
CTACCCCATTTTAGGAT	mmu-mir-130a	0	chr2	84542025	84546150	2784	+	9.03442
CGGCTCCATCTTTAACC	mmu-mir-130b	30	chr16	17038569	17038769	110	+	9.2498
TCTCACCATACTGAAAG	mmu-mir-133a-1	0	chr18	10848725	10849950	18	-	10.3378
TTTCTCCATTTTCTCTC	mmu-mir-134	0	chr12	110043400	1.1E+08	5	-	9.78747
CGTCTTCATTTTTGCAC	mmu-mir-136	-10	chr12	109987400	1.1E+08	1539	-	11.4637
GGACCCCATTTTCACTA	mmu-mir-136	-10	chr12	109987400	1.1E+08	633	+	9.67413
TAAACCCATTTTCCATA	mmu-mir-137	25	chr3	118425075	1.18E+08	1	-	9.45551
TAATTCCATTTATTCAA	mmu-mir-138-1	-10	chr9	122416550	1.22E+08	55	+	11.2988
GCACCCCATCTTTCCTC	mmu-mir-138-1	-10	chr9	122416550	1.22E+08	4393	-	9.84162
ATACACCATTTTTACTT	mmu-mir-138-1	-10	chr9	122416550	1.22E+08	5429	+	9.28225
GAGTACCATTGAGAGAG	mmu-mir-138-1	-10	chr9	122416550	1.22E+08	2584	-	8.8856
AGAGGCCATCTTTGGAT	mmu-mir-141	20	chr6	124683151	1.25E+08	1525	+	10.967
GGCAGCCATTTTGTCTC	mmu-mir-141	20	chr6	124683151	1.25E+08	228	+	9.68977
CAACCCCATCTTTTAAA	mmu-mir-142	0	chr11	87571775	87574325	342	+	10.466
CATCCCCATCTTTAGAA	mmu-mir-142	0	chr11	87571775	87574325	945	-	10.4114
TGGCGCCATGTTGAGTC	mmu-mir-142	0	chr11	87571775	87574325	1099	+	9.9804
GGCGGCCATGTTGCGTG	mmu-mir-142	0	chr11	87571775	87574325	705	-	9.42649
AGTCTTCATTTTGGAGG	mmu-mir-142	0	chr11	87571775	87574325	1932	-	9.17565
CCAGTCCATTCTTTATT	mmu-mir-142	0	chr11	87571775	87574325	1670	-	9.14895
CGCCTCCATTTTTAGAA	mmu-mir-143	20	chr18	61773267	61775675	2277	+	11.6371
GGTGACCATCTTCTGTG	mmu-mir-143	20	chr18	61773267	61775675	2034	+	10.7484
CAGAACCATATACAATG	mmu-mir-143	20	chr18	61773267	61775675	963	+	9.36024
CCTGACCATTCATACAT	mmu-mir-143	20	chr18	61773267	61775675	407	-	8.81873
AGTGGCCATAGTTGAAA	mmu-mir-143	20	chr18	61773267	61775675	1777	-	8.80651

CGCCTCCATTTTTAGAA	mmu-mir-145	20	chr18	61773267	61775675	2277	+	11.6371
GGTGACCATCTTCTGTG	mmu-mir-145	20	chr18	61773267	61775675	2034	+	10.7484
CAGAACCATATACAATG	mmu-mir-145	20	chr18	61773267	61775675	963	+	9.36024
CCTGACCATTCATACAT	mmu-mir-145	20	chr18	61773267	61775675	407	-	8.81873
AGTGGCCATAGTTGAAA	mmu-mir-145	20	chr18	61773267	61775675	1777	-	8.80651
ACACTCCATATTGGACA	mmu-mir-146a	0	chr11	43227200	43230425	752	+	10.0267
GGTGGCCATTTTTGAGA	mmu-mir-146b	0	chr19	46390100	46393375	1899	+	10.6474
GGCGGCCATTTTTGTTT	mmu-mir-146b	0	chr19	46390100	46393375	1992	+	9.78149
GGAAGCCATCTAGTCAT	mmu-mir-148a	15	chr6	51198300	51201975	3187	-	9.28252
CCAGACCATCTAGGGAC	mmu-mir-150	20	chr7	44988600	44989794	535	+	11.5248
GATACCCATCTTTGGGG	mmu-mir-150	20	chr7	44988600	44989794	22	-	8.9583
TTTCTCCATTTTCTCTC	mmu-mir-154	0	chr12	110043400	1.1E+08	5	-	9.78747
GCACACCATGTATGTAT	mmu-mir-155	0	chr16	84584475	84588475	2310	+	10.9221
CGGCGCCATTTTCGAGT	mmu-mir-15a	15	chr14	60602792	60602992	92	-	9.2853
CACCGCCATCTTCGGGC	mmu-mir-15b	25	chr3	69092800	69093000	8	-	9.00833
AGGCGCCATTTTCGAGT	mmu-mir-15b	25	chr3	69092800	69093000	111	+	8.97365
CGGCGCCATTTTCGAGT	mmu-mir-16-1	15	chr14	60602792	60602992	92	-	9.2853
CACCGCCATCTTCGGGC	mmu-mir-16-2	25	chr3	69092800	69093000	8	-	9.00833
AGGCGCCATTTTCGAGT	mmu-mir-16-2	25	chr3	69092800	69093000	111	+	8.97365
CCACTCCAGTTTAAAAC	mmu-mir-17	10	chr14	113921300	1.14E+08	210	+	9.73828
TTTCTCCATTTATTATA	mmu-mir-17	10	chr14	113921300	1.14E+08	513	-	9.62075
TATTGTCATTTTCACAT	mmu-mir-17	10	chr14	113921300	1.14E+08	5062	-	9.04197
CGTGAACATTTTTACAG	mmu-mir-17	10	chr14	113921300	1.14E+08	5101	-	8.94114
CGAAACCAGTTTGAAAT	mmu-mir-17	10	chr14	113921300	1.14E+08	674	+	8.84033
CCCGACCATTTAGAAGC	mmu-mir-181c	10	chr8	87069525	87071900	262	-	9.18028
TATTGCCATACTTACAC	mmu-mir-181c	10	chr8	87069525	87071900	1933	+	9.05639
CCCGACCATTTAGAAGC	mmu-mir-181d	10	chr8	87069525	87071900	262	-	9.18028
TATTGCCATACTTACAC	mmu-mir-181d	10	chr8	87069525	87071900	1933	+	9.05639
TCCCACCATTTTTGGCA	mmu-mir-182	10	chr6	30114875	30130825	1114	-	10.3837
GTTTTCCATTTTTGCAA	mmu-mir-182	10	chr6	30114875	30130825	12149	-	10.0079
TTCCTCCATTTTCTGAG	mmu-mir-182	10	chr6	30114875	30130825	14431	+	9.97098
GCTTGCCATCTACCAAG	mmu-mir-182	10	chr6	30114875	30130825	1503	+	9.74037
ACCCGCCATGTATCAAG	mmu-mir-182	10	chr6	30114875	30130825	13758	+	9.58357
GGAAATCATCTAGGGAG	mmu-mir-182	10	chr6	30114875	30130825	15201	+	9.29284
CCTCCCCATTTCGGAAA	mmu-mir-182	10	chr6	30114875	30130825	13537	-	9.25616
AGTGTCCATCTTACCAA	mmu-mir-182	10	chr6	30114875	30130825	5937	-	9.0865

TCCCACCATTTTTGGCA	mmu-mir-183	10	chr6	30114875	30130825	1114	-	10.3837
GTTTTCCATTTTTGCAA	mmu-mir-183	10	chr6	30114875	30130825	12149	-	10.0079
TTCCTCCATTTTCTGAG	mmu-mir-183	10	chr6	30114875	30130825	14431	+	9.97098
GCTTGCCATCTACCAAG	mmu-mir-183	10	chr6	30114875	30130825	1503	+	9.74037
ACCCGCCATGTATCAAG	mmu-mir-183	10	chr6	30114875	30130825	13758	+	9.58357
GGAAATCATCTAGGGAG	mmu-mir-183	10	chr6	30114875	30130825	15201	+	9.29284
CCTCCCCATTTCGGAAA	mmu-mir-183	10	chr6	30114875	30130825	13537	-	9.25616
AGTGTCCATCTTACCAA	mmu-mir-183	10	chr6	30114875	30130825	5937	-	9.0865
GAATTCCATATCTAAAC	mmu-mir-184	0	chr9	89676975	89678850	529	-	11.0331
GAATTCCATATCTAAAC	mmu-mir-184	0	chr9	89676975	89678850	456	-	11.0331
CAAAATCATATTCACAT	mmu-mir-184	0	chr9	89676975	89678850	332	-	8.87255
CCCGGCCATCTTCAGAG	mmu-mir-185	24	chr16	18261507	18261707	25	-	9.38534
CCACTCCAGTTTAAAAC	mmu-mir-18a	10	chr14	113921300	1.14E+08	210	+	9.73828
TTTCTCCATTTATTATA	mmu-mir-18a	10	chr14	113921300	1.14E+08	513	-	9.62075
TATTGTCATTTTCACAT	mmu-mir-18a	10	chr14	113921300	1.14E+08	5062	-	9.04197
CGTGAACATTTTTACAG	mmu-mir-18a	10	chr14	113921300	1.14E+08	5101	-	8.94114
CGAAACCAGTTTGAAAT	mmu-mir-18a	10	chr14	113921300	1.14E+08	674	+	8.84033
CACAGCCATATATTCAC	mmu-mir-18b	0	chrX	48985925	48991150	711	+	9.75653
GAAACCCATTTTTTTTC	mmu-mir-18b	0	chrX	48985925	48991150	17	+	9.72013
GGTATCCATCTGTAAAC	mmu-mir-18b	0	chrX	48985925	48991150	395	-	8.92002
GGGTTCCATTTCCTAAT	mmu-mir-18b	0	chrX	48985925	48991150	3369	-	8.83589
TCTCTCCATTTAGTAGT	mmu-mir-190b	-15	chr3	90134875	90141050	2356	+	10.4587
TGACTACATTTTGAAAG	mmu-mir-190b	-15	chr3	90134875	90141050	1792	+	10.0921
TATAGTCATATTGAAAG	mmu-mir-190b	-15	chr3	90134875	90141050	1421	+	9.83853
AGAAACCATATAGCATT	mmu-mir-190b	-15	chr3	90134875	90141050	1309	-	9.50883
GAAAACCATATGGAAAT	mmu-mir-190b	-15	chr3	90134875	90141050	3982	+	9.45704
TAGAGCCATTTTAGTAA	mmu-mir-190b	-15	chr3	90134875	90141050	4528	-	9.00641
GATCTCCATCTCTCAGG	mmu-mir-191	20	chr9	108424326	1.08E+08	2244	+	8.94883
GATGACCATTTTGAGAC	mmu-mir-193	10	chr11	79526175	79531800	3113	+	13.8923
GGTGCCCATCTTCCAAG	mmu-mir-193	10	chr11	79526175	79531800	3800	-	10.0297
AAAGACCATATAAGAGG	mmu-mir-193	10	chr11	79526175	79531800	2797	+	9.61794
ACATTCCATATATGCAC	mmu-mir-193b	15	chr16	13359750	13363900	4012	+	11.0176
GATGACCATGTTCTCTG	mmu-mir-193b	15	chr16	13359750	13363900	171	+	10.7119
TATTTCCATCTTTAATA	mmu-mir-193b	15	chr16	13359750	13363900	2942	-	10.4289
TCAATCCAGATTGGGAG	mmu-mir-193b	15	chr16	13359750	13363900	2963	-	9.43238
TCTCCCCATTTTAGCCC	mmu-mir-193b	15	chr16	13359750	13363900	2874	-	9.19802

CACATCCATATTTATAA	mmu-mir-193b	15	chr16	13359750	13363900	2824	+	9.18864
TCAGTCCATGTATGAAT	mmu-mir-194-1	0	chr1	186908300	1.87E+08	3345	+	11.741
ATAGTCCATTTTTGAAG	mmu-mir-194-1	0	chr1	186908300	1.87E+08	618	-	11.0625
ACACACCATCTTAAATC	mmu-mir-195	15	chr11	70048125	70050450	1856	+	10.2811
CAGGACCATCTTCCCAA	mmu-mir-195	15	chr11	70048125	70050450	1066	+	9.26701
AAAGACCATTTTTGTTG	mmu-mir-196a-1	-10	chr11	96075625	96077750	1695	-	11.3089
AGACACCATTTTTCTGG	mmu-mir-196a-1	-10	chr11	96075625	96077750	1249	-	9.39378
GTGTTCCATATATGAAT	mmu-mir-196a-1	-10	chr11	96075625	96077750	1437	-	9.03442
AGACACCATGTTGAGAA	mmu-mir-196a-2	10	chr15	102799650	1.03E+08	2929	-	10.8794
GCTGACCATTTTCAATT	mmu-mir-196b	10	chr6	52159743	52171275	6002	-	11.7446
ACATTCCATATTTAATG	mmu-mir-196b	10	chr6	52159743	52171275	8725	+	10.6337
GAAGGCCAGATTTAAAA	mmu-mir-196b	10	chr6	52159743	52171275	9287	-	9.43682
CCATGCCATTTAAGCAA	mmu-mir-196b	10	chr6	52159743	52171275	9369	+	9.39002
ACCCATCATTTTCCAAG	mmu-mir-196b	10	chr6	52159743	52171275	7261	+	9.35377
TGAAACCAGATTTTCAC	mmu-mir-196b	10	chr6	52159743	52171275	2424	+	8.8906
CATTCCCATCTTAGGAG	mmu-mir-199a-1	10	chr9	21244900	21248875	1003	+	9.52816
CCTGGCCATATTTTTTA	mmu-mir-199a-1	10	chr9	21244900	21248875	1284	+	8.84213
TCATCCCATATATGGAC	mmu-mir-199a-2	10	chr1	164052250	1.64E+08	2011	+	10.7411
GAACACCATGTAAACAA	mmu-mir-199a-2	10	chr1	164052250	1.64E+08	1097	+	10.523
ACACACCATTTTCAAGG	mmu-mir-199a-2	10	chr1	164052250	1.64E+08	4473	+	10.3232
TAACACCATGTCTTCAT	mmu-mir-199a-2	10	chr1	164052250	1.64E+08	4584	-	10.0418
GACCATCATATAGAGAT	mmu-mir-199a-2	10	chr1	164052250	1.64E+08	5522	+	9.87096
AGAAACCATGTTAGGAA	mmu-mir-199a-2	10	chr1	164052250	1.64E+08	659	+	9.66192
GATGTCCATGTAAATAT	mmu-mir-199a-2	10	chr1	164052250	1.64E+08	5655	+	9.55843
GTATTCCATTTTTGTAT	mmu-mir-199a-2	10	chr1	164052250	1.64E+08	6459	+	9.53656
GATTGTCATTTTGGATA	mmu-mir-199a-2	10	chr1	164052250	1.64E+08	6990	+	9.51435
TCTCTTCATATTAAATG	mmu-mir-199a-2	10	chr1	164052250	1.64E+08	4272	+	9.49556
AAAGCCCATTTCTGAAA	mmu-mir-199a-2	10	chr1	164052250	1.64E+08	8111	+	9.35137
AGAGTCCATATATGGGA	mmu-mir-199a-2	10	chr1	164052250	1.64E+08	2014	-	9.31512
GCCTTTCATTTTTAAAA	mmu-mir-199a-2	10	chr1	164052250	1.64E+08	9053	+	9.18259
AACAATCATTTATAGAC	mmu-mir-199a-2	10	chr1	164052250	1.64E+08	8289	-	9.133
GAAGTCCAGATATAGTT	mmu-mir-199a-2	10	chr1	164052250	1.64E+08	6565	+	8.96301
GGAACCCATCTAGGGAG	mmu-mir-199b	15	chr2	32138775	32141975	1391	+	9.7112
TGACTTCATTTCCTGAC	mmu-mir-199b	15	chr2	32138775	32141975	605	+	9.19111
GCTTGCCATTGTGGATG	mmu-mir-199b	15	chr2	32138775	32141975	876	+	8.98297
CCACTCCAGTTTAAAAC	mmu-mir-19a	10	chr14	113921300	1.14E+08	210	+	9.73828

TTTCTCCATTTATTATA	mmu-mir-19a	10	chr14	113921300	1.14E+08	513	-	9.62075
TATTGTCATTTTCACAT	mmu-mir-19a	10	chr14	113921300	1.14E+08	5062	-	9.04197
CGTGAACATTTTTACAG	mmu-mir-19a	10	chr14	113921300	1.14E+08	5101	-	8.94114
CGAAACCAGTTTGAAAT	mmu-mir-19a	10	chr14	113921300	1.14E+08	674	+	8.84033
CACAGCCATATATTCAC	mmu-mir-19b	0	chrX	48985925	48991150	711	+	9.75653
GAAACCCATTTTTTTTC	mmu-mir-19b	0	chrX	48985925	48991150	17	+	9.72013
GGTATCCATCTGTAAAC	mmu-mir-19b	0	chrX	48985925	48991150	395	-	8.92002
GGGTTCCATTTCCTAAT	mmu-mir-19b	0	chrX	48985925	48991150	3369	-	8.83589
GAACACCATGTAGGGAG	mmu-mir-200a	15	chr4	154903109	1.55E+08	55	-	12.8833
GAACACCATGTAGGGAG	mmu-mir-200b	15	chr4	154903109	1.55E+08	55	-	12.8833
AGAGGCCATCTTTGGAT	mmu-mir-200c	20	chr6	124683151	1.25E+08	1525	+	10.967
GGCAGCCATTTTGTCTC	mmu-mir-200c	20	chr6	124683151	1.25E+08	228	+	9.68977
GCTGTCCATTCATACAG	mmu-mir-203	15	chr12	112577075	1.13E+08	2122	-	9.64884
CATCTCCCTTTTGTCAC	mmu-mir-203	15	chr12	112577075	1.13E+08	71	+	9.28392
AGAAGCCATTTACTAAT	mmu-mir-205	0	chr1	195208350	1.95E+08	559	-	9.91412
CCACTCCAGTTTAAAAC	mmu-mir-20a	10	chr14	113921300	1.14E+08	210	+	9.73828
TTTCTCCATTTATTATA	mmu-mir-20a	10	chr14	113921300	1.14E+08	513	-	9.62075
TATTGTCATTTTCACAT	mmu-mir-20a	10	chr14	113921300	1.14E+08	5062	-	9.04197
CGTGAACATTTTTACAG	mmu-mir-20a	10	chr14	113921300	1.14E+08	5101	-	8.94114
CGAAACCAGTTTGAAAT	mmu-mir-20a	10	chr14	113921300	1.14E+08	674	+	8.84033
CACAGCCATATATTCAC	mmu-mir-20b	0	chrX	48985925	48991150	711	+	9.75653
GAAACCCATTTTTTTTC	mmu-mir-20b	0	chrX	48985925	48991150	17	+	9.72013
GGTATCCATCTGTAAAC	mmu-mir-20b	0	chrX	48985925	48991150	395	-	8.92002
GGGTTCCATTTCCTAAT	mmu-mir-20b	0	chrX	48985925	48991150	3369	-	8.83589
TCATCCCATATATGGAC	mmu-mir-214	10	chr1	164052250	1.64E+08	2011	+	10.7411
GAACACCATGTAAACAA	mmu-mir-214	10	chr1	164052250	1.64E+08	1097	+	10.523
ACACACCATTTTCAAGG	mmu-mir-214	10	chr1	164052250	1.64E+08	4473	+	10.3232
TAACACCATGTCTTCAT	mmu-mir-214	10	chr1	164052250	1.64E+08	4584	-	10.0418
GACCATCATATAGAGAT	mmu-mir-214	10	chr1	164052250	1.64E+08	5522	+	9.87096
AGAAACCATGTTAGGAA	mmu-mir-214	10	chr1	164052250	1.64E+08	659	+	9.66192
GATGTCCATGTAAATAT	mmu-mir-214	10	chr1	164052250	1.64E+08	5655	+	9.55843
GTATTCCATTTTTGTAT	mmu-mir-214	10	chr1	164052250	1.64E+08	6459	+	9.53656
GATTGTCATTTTGGATA	mmu-mir-214	10	chr1	164052250	1.64E+08	6990	+	9.51435
TCTCTTCATATTAAATG	mmu-mir-214	10	chr1	164052250	1.64E+08	4272	+	9.49556
AAAGCCCATTTCTGAAA	mmu-mir-214	10	chr1	164052250	1.64E+08	8111	+	9.35137
AGAGTCCATATATGGGA	mmu-mir-214	10	chr1	164052250	1.64E+08	2014	-	9.31512

GCCTTTCATTTTTAAAA	mmu-mir-214	10	chr1	164052250	1.64E+08	9053	+	9.18259
AACAATCATTTATAGAC	mmu-mir-214	10	chr1	164052250	1.64E+08	8289	-	9.133
GAAGTCCAGATATAGTT	mmu-mir-214	10	chr1	164052250	1.64E+08	6565	+	8.96301
TCAGTCCATGTATGAAT	mmu-mir-215	0	chr1	186908300	1.87E+08	3345	+	11.741
ATAGTCCATTTTTGAAG	mmu-mir-215	0	chr1	186908300	1.87E+08	618	-	11.0625
CAGCTCCATTTAAACAA	mmu-mir-220	0	chr6	136427625	1.36E+08	234	+	10.1241
GAATGCCATTTATGAAC	mmu-mir-221	-10	chrX	18319475	18326525	2107	+	12.9403
GATCTCCATTTTTCAGG	mmu-mir-221	-10	chrX	18319475	18326525	2022	-	12.2627
AATCTCCATTTATGAGT	mmu-mir-221	-10	chrX	18319475	18326525	3294	-	11.134
AAGTACCATTTTCAGAG	mmu-mir-221	-10	chrX	18319475	18326525	5586	-	10.8105
GGAAACCATGTTCAAAA	mmu-mir-221	-10	chrX	18319475	18326525	4042	+	10.4155
TGAGGCCATTGTTGAAT	mmu-mir-221	-10	chrX	18319475	18326525	6327	-	10.3195
TAAAACCATTGTGAAAT	mmu-mir-221	-10	chrX	18319475	18326525	1343	+	10.0192
CATTACCATCTTAAAAA	mmu-mir-221	-10	chrX	18319475	18326525	3672	+	9.98884
GCAGTTCATTTATAGTT	mmu-mir-221	-10	chrX	18319475	18326525	5077	-	9.79075
AAACTTCATCTTTAAAA	mmu-mir-221	-10	chrX	18319475	18326525	2526	-	9.73564
GGAATCCAGTTTTAAAT	mmu-mir-221	-10	chrX	18319475	18326525	6176	+	9.69069
GATTCCCATCTTGGCTG	mmu-mir-221	-10	chrX	18319475	18326525	4994	-	9.33244
TATTTCCATGTGTGAAT	mmu-mir-221	-10	chrX	18319475	18326525	6841	-	9.23795
TACTTTCATTTTGTAAA	mmu-mir-221	-10	chrX	18319475	18326525	3851	+	9.20584
GAAGATCATTTTTCTTA	mmu-mir-221	-10	chrX	18319475	18326525	2979	-	9.01445
GATGTACATTTAAAAAT	mmu-mir-221	-10	chrX	18319475	18326525	2477	+	8.91142
CCCCACCATATAACATA	mmu-mir-221	-10	chrX	18319475	18326525	4145	-	8.84884
GAATGCCATTTATGAAC	mmu-mir-222	-10	chrX	18319475	18326525	2107	+	12.9403
GATCTCCATTTTTCAGG	mmu-mir-222	-10	chrX	18319475	18326525	2022	-	12.2627
AATCTCCATTTATGAGT	mmu-mir-222	-10	chrX	18319475	18326525	3294	-	11.134
AAGTACCATTTTCAGAG	mmu-mir-222	-10	chrX	18319475	18326525	5586	-	10.8105
GGAAACCATGTTCAAAA	mmu-mir-222	-10	chrX	18319475	18326525	4042	+	10.4155
TGAGGCCATTGTTGAAT	mmu-mir-222	-10	chrX	18319475	18326525	6327	-	10.3195
TAAAACCATTGTGAAAT	mmu-mir-222	-10	chrX	18319475	18326525	1343	+	10.0192
CATTACCATCTTAAAAA	mmu-mir-222	-10	chrX	18319475	18326525	3672	+	9.98884
GCAGTTCATTTATAGTT	mmu-mir-222	-10	chrX	18319475	18326525	5077	-	9.79075
AAACTTCATCTTTAAAA	mmu-mir-222	-10	chrX	18319475	18326525	2526	-	9.73564
GGAATCCAGTTTTAAAT	mmu-mir-222	-10	chrX	18319475	18326525	6176	+	9.69069
GATTCCCATCTTGGCTG	mmu-mir-222	-10	chrX	18319475	18326525	4994	-	9.33244
TATTTCCATGTGTGAAT	mmu-mir-222	-10	chrX	18319475	18326525	6841	-	9.23795

TACTTTCATTTTGTAAA	mmu-mir-222	-10	chrX	18319475	18326525	3851	+	9.20584
GAAGATCATTTTTCTTA	mmu-mir-222	-10	chrX	18319475	18326525	2979	-	9.01445
GATGTACATTTAAAAAT	mmu-mir-222	-10	chrX	18319475	18326525	2477	+	8.91142
CCCCACCATATAACATA	mmu-mir-222	-10	chrX	18319475	18326525	4145	-	8.84884
TACTTTCATCTTGGGAG	mmu-mir-223	-10	chrX	92297875	92303675	4955	+	9.09392
TGAGACCATATTGTGTA	mmu-mir-23a	0	chr8	87086300	87095525	2655	-	11.0069
GAACACCATCTTTCTGC	mmu-mir-23a	0	chr8	87086300	87095525	3936	-	9.81584
TCATTCCATTTTGCTTT	mmu-mir-23a	0	chr8	87086300	87095525	2962	-	9.6332
TGACTCCATGCTTCCAG	mmu-mir-23a	0	chr8	87086300	87095525	6895	+	9.19503
CATTTCCATATACTGCC	mmu-mir-23a	0	chr8	87086300	87095525	3847	-	9.18382
TACAATCATTTTGAAAC	mmu-mir-23b	9	chr13	63284054	63284254	118	-	10.1235
CGAATCCATTTCGCCAC	mmu-mir-23b	7	chr13	63249379	63249579	162	-	9.40181
TACAATCATTTTGAAAC	mmu-mir-24-1	9	chr13	63284054	63284254	118	-	10.1235
CGAATCCATTTCGCCAC	mmu-mir-24-1	7	chr13	63249379	63249579	162	-	9.40181
TGAGACCATATTGTGTA	mmu-mir-24-2	0	chr8	87086300	87095525	2655	-	11.0069
GAACACCATCTTTCTGC	mmu-mir-24-2	0	chr8	87086300	87095525	3936	-	9.81584
TCATTCCATTTTGCTTT	mmu-mir-24-2	0	chr8	87086300	87095525	2962	-	9.6332
TGACTCCATGCTTCCAG	mmu-mir-24-2	0	chr8	87086300	87095525	6895	+	9.19503
CATTTCCATATACTGCC	mmu-mir-24-2	0	chr8	87086300	87095525	3847	-	9.18382
GGGTTCCATGTTTGCAT	mmu-mir-26b	30	chr1	74324705	74324905	164	+	10.2416
TGAGACCATATTGTGTA	mmu-mir-27a	0	chr8	87086300	87095525	2655	-	11.0069
GAACACCATCTTTCTGC	mmu-mir-27a	0	chr8	87086300	87095525	3936	-	9.81584
TCATTCCATTTTGCTTT	mmu-mir-27a	0	chr8	87086300	87095525	2962	-	9.6332
TGACTCCATGCTTCCAG	mmu-mir-27a	0	chr8	87086300	87095525	6895	+	9.19503
CATTTCCATATACTGCC	mmu-mir-27a	0	chr8	87086300	87095525	3847	-	9.18382
TACAATCATTTTGAAAC	mmu-mir-27b	9	chr13	63284054	63284254	118	-	10.1235
CGAATCCATTTCGCCAC	mmu-mir-27b	7	chr13	63249379	63249579	162	-	9.40181
CAAGGCCATTTTCTGTG	mmu-mir-296	-15	chr2	173925825	1.74E+08	5082	+	11.3128
TGTCTCCAGTTTTAGTA	mmu-mir-296	-15	chr2	173925825	1.74E+08	6629	+	9.28002
GCACACCATACTCATAC	mmu-mir-296	-15	chr2	173925825	1.74E+08	7150	+	8.97742
GCACGTCATATACACAC	mmu-mir-297a-6	0	chr2	169051150	1.69E+08	1355	-	9.32676
AATGTCCATTTTGTATT	mmu-mir-297a-7	-10	chr10	42708700	42711800	2516	+	11.6907
GTACTCCATTTTGTCAG	mmu-mir-297a-7	-10	chr10	42708700	42711800	2881	-	10.9536
TGTCTTCATATTTTCAC	mmu-mir-297a-7	-10	chr10	42708700	42711800	3044	+	10.8594
CAGAGCCATCTTTGAAA	mmu-mir-297a-7	-10	chr10	42708700	42711800	2867	-	9.84574
AAACGCCATGTACCCAA	mmu-mir-297a-7	-10	chr10	42708700	42711800	2680	-	9.25771

TATCATCATTTCCCAAA	mmu-mir-297a-7	-10	chr10	42708700	42711800	2727	+	8.8388
CAAGGCCATTTTCTGTG	mmu-mir-298	-15	chr2	173925825	1.74E+08	5082	+	11.3128
TGTCTCCAGTTTTAGTA	mmu-mir-298	-15	chr2	173925825	1.74E+08	6629	+	9.28002
GCACACCATACTCATAC	mmu-mir-298	-15	chr2	173925825	1.74E+08	7150	+	8.97742
TTTCTCCATTTTCTCTC	mmu-mir-299	0	chr12	110043400	1.1E+08	5	-	9.78747
GACGGCCATTTTGGTTT	mmu-mir-29b-2	-10	chr1	196676100	1.97E+08	3600	+	10.013
CATCTCCATGTAGGAGA	mmu-mir-29b-2	-10	chr1	196676100	1.97E+08	2368	+	9.59209
TAGCCCCATTTTTCCTA	mmu-mir-29b-2	-10	chr1	196676100	1.97E+08	1764	-	9.52251
GAACACCATGTTTATCT	mmu-mir-29b-2	-10	chr1	196676100	1.97E+08	2794	-	9.39645
ATTGTCCATTTTTTATA	mmu-mir-29b-2	-10	chr1	196676100	1.97E+08	3004	+	9.05154
GCTGGCCAGTTTAGCAG	mmu-mir-29b-2	-10	chr1	196676100	1.97E+08	261	-	8.90666
GACGGCCATTTTGGTTT	mmu-mir-29c	-10	chr1	196676100	1.97E+08	3600	+	10.013
CATCTCCATGTAGGAGA	mmu-mir-29c	-10	chr1	196676100	1.97E+08	2368	+	9.59209
TAGCCCCATTTTTCCTA	mmu-mir-29c	-10	chr1	196676100	1.97E+08	1764	-	9.52251
GAACACCATGTTTATCT	mmu-mir-29c	-10	chr1	196676100	1.97E+08	2794	-	9.39645
ATTGTCCATTTTTTATA	mmu-mir-29c	-10	chr1	196676100	1.97E+08	3004	+	9.05154
GCTGGCCAGTTTAGCAG	mmu-mir-29c	-10	chr1	196676100	1.97E+08	261	-	8.90666
TTTCTCCATTTTCTCTC	mmu-mir-300	0	chr12	110043400	1.1E+08	5	-	9.78747
CGCCTCCATATTGAATA	mmu-mir-301a	25	chr11	86925352	86925552	106	-	10.1189
CGGCTCCATCTTTAACC	mmu-mir-301b	30	chr16	17038569	17038769	110	+	9.2498
CATCACCATTGCTAAAG	mmu-mir-302a	10	chr3	127537000	1.28E+08	807	-	9.17836
CATCACCATTGCTAAAG	mmu-mir-302b	10	chr3	127537000	1.28E+08	807	-	9.17836
CATCACCATTGCTAAAG	mmu-mir-302c	10	chr3	127537000	1.28E+08	807	-	9.17836
CATCACCATTGCTAAAG	mmu-mir-302d	10	chr3	127537000	1.28E+08	807	-	9.17836
TAAATTCATTTAGAAAA	mmu-mir-30a	-5	chr1	23209700	23213275	3075	+	9.70983
GGAATGCATATTTAAAG	mmu-mir-30a	-5	chr1	23209700	23213275	2856	+	9.0199
GCCTACCATTTCACAAC	mmu-mir-30a	-5	chr1	23209700	23213275	1717	-	8.93278
TGTCTCCATTTTCCAAA	mmu-mir-30b	-8	chr15	68190175	68194550	555	-	12.3659
CCTCTCCATATTGGCCC	mmu-mir-30b	-8	chr15	68190175	68194550	2966	-	10.5553
GGGCTCCATAGTGAGAC	mmu-mir-30b	-8	chr15	68190175	68194550	3471	-	9.77395
GATAGCCATATTCAAGG	mmu-mir-30b	-8	chr15	68190175	68194550	95	+	9.59943
ATTGTCCATATTTTCAT	mmu-mir-30b	-8	chr15	68190175	68194550	49	-	9.17525
TGTGATCATTTAATGTC	mmu-mir-30b	-8	chr15	68190175	68194550	3588	-	9.15114
AAACAACATTTACTAAG	mmu-mir-30b	-8	chr15	68190175	68194550	182	+	9.06012
AACGATCATCTTGTGAC	mmu-mir-30b	-8	chr15	68190175	68194550	3319	-	8.79907
TAAATTCATTTAGAAAA	mmu-mir-30c-2	-5	chr1	23209700	23213275	3075	+	9.70983

GGAATGCATATTTAAAG	mmu-mir-30c-2	-5	chr1	23209700	23213275	2856	+	9.0199
GCCTACCATTTCACAAC	mmu-mir-30c-2	-5	chr1	23209700	23213275	1717	-	8.93278
AAGCACCATATAGCAAG	mmu-mir-31	0	chr4	88399300	88401975	1718	-	11.2161
GCTCACCATTTAGAACT	mmu-mir-31	0	chr4	88399300	88401975	1112	-	10.6856
AATGATCATCTTGGGAA	mmu-mir-31	0	chr4	88399300	88401975	1811	-	9.45383
CCTTACCATTGAGAAAG	mmu-mir-31	0	chr4	88399300	88401975	1759	-	8.7976
CAAGGCCATGTAGCGAG	mmu-mir-320	2	chr14	69176250	69180100	999	+	10.6731
AGGCTCCATTTCCTGAG	mmu-mir-320	2	chr14	69176250	69180100	315	-	9.25693
TATGTACATTTTCTGAA	mmu-mir-320	2	chr14	69176250	69180100	3425	-	9.11526
TGTGTCCATTTTGAAAA	mmu-mir-322	0	chrX	49298875	49304275	775	-	12.3276
CGTGTCCATGTAAAAAT	mmu-mir-322	0	chrX	49298875	49304275	1193	-	9.95205
CGGCGCCATTTTCGAGT	mmu-mir-322	0	chrX	49298875	49304275	2419	-	9.2853
GCGCTTCATGTTTTGAA	mmu-mir-322	0	chrX	49298875	49304275	28	+	9.03749
GCCGGCCATTTCAAGAC	mmu-mir-322	0	chrX	49298875	49304275	727	-	9
TGCGATCATTTTCGCAA	mmu-mir-322	0	chrX	49298875	49304275	4836	-	8.9176
TGGGGCCATTTTGACCC	mmu-mir-322	0	chrX	49298875	49304275	1724	-	8.87392
TTTCTCCATTTTCTCTC	mmu-mir-323	0	chr12	110043400	1.1E+08	5	-	9.78747
CAGGTCCATTTTCAGAA	mmu-mir-326	10	chr7	99425150	99427125	374	+	11.1215
TCAGACCATCTTCTGTG	mmu-mir-327	-10	chr14	43909450	43912875	3190	-	10.5278
CATCACCATATTAATAG	mmu-mir-328	-15	chr8	108213550	1.08E+08	329	+	11.0007
ACTGACCATTCATGCAC	mmu-mir-328	-15	chr8	108213550	1.08E+08	3846	-	9.50628
TCTATCCATTTTGCCCT	mmu-mir-328	-15	chr8	108213550	1.08E+08	1165	-	9.21221
GCTGGCCATAGTGGCAT	mmu-mir-328	-15	chr8	108213550	1.08E+08	2984	+	9.12315
TTTCTCCATTTTCTCTC	mmu-mir-329	0	chr12	110043400	1.1E+08	5	-	9.78747
CGCCGCCATGTTTGAGC	mmu-mir-33	17	chr15	81974358	81974558	158	-	9.18111
CCTGTTCATTTCTGAAC	mmu-mir-331	0	chr10	93443775	93445175	829	-	9.53449
CGTCTTCATTTTTGCAC	mmu-mir-337	-10	chr12	109987400	1.1E+08	1539	-	11.4637
GGACCCCATTTTCACTA	mmu-mir-337	-10	chr12	109987400	1.1E+08	633	+	9.67413
CATCTCCATATTTCCTT	mmu-mir-339	10	chr5	139624300	1.4E+08	2155	-	11.5914
CATTTCCATTTCCAGAG	mmu-mir-339	10	chr5	139624300	1.4E+08	1888	+	9.98763
AGACCCCATTTTCTGCC	mmu-mir-339	10	chr5	139624300	1.4E+08	812	-	8.93323
AAAGGCCATTTCTCCTG	mmu-mir-339	10	chr5	139624300	1.4E+08	476	+	8.88172
TTTCTCCATTTTCTCTC	mmu-mir-341	0	chr12	110043400	1.1E+08	5	-	9.78747
TCAGTCCATTCTCTATT	mmu-mir-344-1	0	chr7	61818450	61820325	668	-	8.89725
AAATTCCATTTCTTGTA	mmu-mir-344-1	0	chr7	61818450	61820325	1776	-	8.81826
TCAGTCCATTCTCTATT	mmu-mir-344-2	10	chr7	61818450	61820325	668	-	8.89725

AAATTCCATTTCTTGTA	mmu-mir-344-2	10	chr7	61818450	61820325	1776	-	8.81826
CCTCACCATTCAGGGAT	mmu-mir-34a	0	chr4	148890450	1.49E+08	5285	+	10.054
GGGCTCCATAGTGAAAC	mmu-mir-34a	0	chr4	148890450	1.49E+08	431	+	9.82818
CGTTTCCATTTATTAGC	mmu-mir-34a	0	chr4	148890450	1.49E+08	4032	+	9.8047
TATCATCATCTCTGGAG	mmu-mir-34a	0	chr4	148890450	1.49E+08	2767	+	9.40223
GATATCCAGCTATGGAG	mmu-mir-34a	0	chr4	148890450	1.49E+08	4094	+	9.14515
CGGCTCCATTGTGACAC	mmu-mir-34a	0	chr4	148890450	1.49E+08	1278	-	9.06497
GAAAGCCAGATTGGCAG	mmu-mir-34a	0	chr4	148890450	1.49E+08	4585	-	9.01799
AAATTCCATTTTTGGAA	mmu-mir-350	0	chr1	178636600	1.79E+08	3015	-	12.2701
GAACAGCATTTTTCATT	mmu-mir-350	0	chr1	178636600	1.79E+08	1120	-	9.90782
TACTTTCATTTATAAAG	mmu-mir-350	0	chr1	178636600	1.79E+08	1885	+	9.60161
AAAATCCATTTTCATTT	mmu-mir-350	0	chr1	178636600	1.79E+08	796	+	9.26534
GCTAGCCATATTGGTTT	mmu-mir-351	10	chrX	49296350	49298075	89	-	9.38398
AGCAGCCATTTTCTCAG	mmu-mir-351	10	chrX	49296350	49298075	1661	+	9.07849
CACAGCCATATATTCAC	mmu-mir-363	0	chrX	48985925	48991150	711	+	9.75653
GAAACCCATTTTTTTTC	mmu-mir-363	0	chrX	48985925	48991150	17	+	9.72013
GGTATCCATCTGTAAAC	mmu-mir-363	0	chrX	48985925	48991150	395	-	8.92002
GGGTTCCATTTCCTAAT	mmu-mir-363	0	chrX	48985925	48991150	3369	-	8.83589
ACATTCCATATATGCAC	mmu-mir-365-1	6	chr16	13359750	13363900	4012	+	11.0176
GATGACCATGTTCTCTG	mmu-mir-365-1	6	chr16	13359750	13363900	171	+	10.7119
TATTTCCATCTTTAATA	mmu-mir-365-1	6	chr16	13359750	13363900	2942	-	10.4289
TCAATCCAGATTGGGAG	mmu-mir-365-1	6	chr16	13359750	13363900	2963	-	9.43238
TCTCCCCATTTTAGCCC	mmu-mir-365-1	6	chr16	13359750	13363900	2874	-	9.19802
CACATCCATATTTATAA	mmu-mir-365-1	6	chr16	13359750	13363900	2824	+	9.18864
GATGACCATTTTGAGAC	mmu-mir-365-2	0	chr11	79526175	79531800	3113	+	13.8923
GGTGCCCATCTTCCAAG	mmu-mir-365-2	0	chr11	79526175	79531800	3800	-	10.0297
AAAGACCATATAAGAGG	mmu-mir-365-2	0	chr11	79526175	79531800	2797	+	9.61794
CATCACCATTGCTAAAG	mmu-mir-367	10	chr3	127537000	1.28E+08	807	-	9.17836
TTTCTCCATTTTCTCTC	mmu-mir-368	0	chr12	110043400	1.1E+08	5	-	9.78747
TTTCTCCATTTTCTCTC	mmu-mir-369	0	chr12	110043400	1.1E+08	5	-	9.78747
TTTCTCCATTTTCTCTC	mmu-mir-370	0	chr12	110043400	1.1E+08	5	-	9.78747
TCTGACCATTTTCAATA	mmu-mir-374	0	chrX	99817575	99820975	488	+	11.159
CATTTCCATTGTTGGAA	mmu-mir-374	0	chrX	99817575	99820975	378	-	9.97034
GCACAGCATTTATGATG	mmu-mir-374	0	chrX	99817575	99820975	2339	+	9.66315
GAATTCCATCTTCCGGG	mmu-mir-374	0	chrX	99817575	99820975	2537	+	9.29531
TTTCTCCATTTTCTCTC	mmu-mir-376a	0	chr12	110043400	1.1E+08	5	-	9.78747

TTTCTCCATTTTCTCTC	mmu-mir-376b	0	chr12	110043400	1.1E+08	5	-	9.78747
TTTCTCCATTTTCTCTC	mmu-mir-377	0	chr12	110043400	1.1E+08	5	-	9.78747
TTTCTCCATTTTCTCTC	mmu-mir-379	0	chr12	110043400	1.1E+08	5	-	9.78747
TTTCTCCATTTTCTCTC	mmu-mir-380	0	chr12	110043400	1.1E+08	5	-	9.78747
TTTCTCCATTTTCTCTC	mmu-mir-381	0	chr12	110043400	1.1E+08	5	-	9.78747
TTTCTCCATTTTCTCTC	mmu-mir-382	0	chr12	110043400	1.1E+08	5	-	9.78747
TCAGACCATCTTTCCAC	mmu-mir-384	-10	chrX	101592850	1.02E+08	2220	+	11.5686
TCTGGCCATTTTCAACC	mmu-mir-384	-10	chrX	101592850	1.02E+08	1517	+	10.1363
TTTCTCCATTTTCTCTC	mmu-mir-409	0	chr12	110043400	1.1E+08	5	-	9.78747
TTTCTCCATTTTCTCTC	mmu-mir-410	0	chr12	110043400	1.1E+08	5	-	9.78747
TTTCTCCATTTTCTCTC	mmu-mir-411	0	chr12	110043400	1.1E+08	5	-	9.78747
TTTCTCCATTTTCTCTC	mmu-mir-412	0	chr12	110043400	1.1E+08	5	-	9.78747
GCCCTCCATGTTACATT	mmu-mir-421	0	chrX	99822432	99826775	3793	+	9.58518
GAACACCAGGTACGAAA	mmu-mir-421	0	chrX	99822432	99826775	3952	+	9.08194
GATCGCCATCTTGTTCC	mmu-mir-423	35	chr11	76894530	76894730	54	+	8.93757
GATCTCCATCTCTCAGG	mmu-mir-425	20	chr9	108424326	1.08E+08	2244	+	8.94883
GAACACCATGTAGGGAG	mmu-mir-429	15	chr4	154903109	1.55E+08	55	-	12.8833
CGTCTTCATTTTTGCAC	mmu-mir-431	-10	chr12	109987400	1.1E+08	1539	-	11.4637
GGACCCCATTTTCACTA	mmu-mir-431	-10	chr12	109987400	1.1E+08	633	+	9.67413
CGTCTTCATTTTTGCAC	mmu-mir-433	-10	chr12	109987400	1.1E+08	1539	-	11.4637
GGACCCCATTTTCACTA	mmu-mir-433	-10	chr12	109987400	1.1E+08	633	+	9.67413
CGTCTTCATTTTTGCAC	mmu-mir-434	-10	chr12	109987400	1.1E+08	1539	-	11.4637
GGACCCCATTTTCACTA	mmu-mir-434	-10	chr12	109987400	1.1E+08	633	+	9.67413
AGAATCCATTTCTTAAG	mmu-mir-449a	15	chr13	114154825	1.14E+08	1957	-	9.84312
AGAATCCATTTCTTAAG	mmu-mir-449b	15	chr13	114154825	1.14E+08	1957	-	9.84312
GCTAGCCATATTGGTTT	mmu-mir-450a-1	0	chrX	49296350	49298075	89	-	9.38398
AGCAGCCATTTTCTCAG	mmu-mir-450a-1	0	chrX	49296350	49298075	1661	+	9.07849
GCTAGCCATATTGGTTT	mmu-mir-450a-2	0	chrX	49296350	49298075	89	-	9.38398
AGCAGCCATTTTCTCAG	mmu-mir-450a-2	0	chrX	49296350	49298075	1661	+	9.07849
GCTAGCCATATTGGTTT	mmu-mir-450b	0	chrX	49296350	49298075	89	-	9.38398
AGCAGCCATTTTCTCAG	mmu-mir-450b	0	chrX	49296350	49298075	1661	+	9.07849
TATCATCATATACTGTA	mmu-mir-451	0	chr11	77887875	77889475	1332	+	9.10851
TTTCTCCATTTTCTCTC	mmu-mir-453	0	chr12	110043400	1.1E+08	5	-	9.78747
GAATTCCATTTAAGCAG	mmu-mir-484	2	chr16	14070875	14074575	854	+	11.7445
GAACCCCAGTTTAGGAG	mmu-mir-484	2	chr16	14070875	14074575	2408	+	9.5496
TAAGACCCTATTTTGAG	mmu-mir-484	2	chr16	14070875	14074575	2358	-	9.4311

CAGGGTCATTTATTGAT	mmu-mir-484	2	chr16	14070875	14074575	1308	-	8.98289
TTTCTCCATTTTCTCTC	mmu-mir-485	0	chr12	110043400	1.1E+08	5	-	9.78747
TTTCTCCATTTTCTCTC	mmu-mir-487b	0	chr12	110043400	1.1E+08	5	-	9.78747
CGTCTTCATTTTTGCAC	mmu-mir-493	-10	chr12	109987400	1.1E+08	1539	-	11.4637
GGACCCCATTTTCACTA	mmu-mir-493	-10	chr12	109987400	1.1E+08	633	+	9.67413
TTTCTCCATTTTCTCTC	mmu-mir-494	0	chr12	110043400	1.1E+08	5	-	9.78747
TTTCTCCATTTTCTCTC	mmu-mir-495	0	chr12	110043400	1.1E+08	5	-	9.78747
TTTCTCCATTTTCTCTC	mmu-mir-496	0	chr12	110043400	1.1E+08	5	-	9.78747
ACACACCATCTTAAATC	mmu-mir-497	15	chr11	70048125	70050450	1856	+	10.2811
CAGGACCATCTTCCCAA	mmu-mir-497	15	chr11	70048125	70050450	1066	+	9.26701
TGTGTCCATTTTGAAAA	mmu-mir-503	0	chrX	49298875	49304275	775	-	12.3276
CGTGTCCATGTAAAAAT	mmu-mir-503	0	chrX	49298875	49304275	1193	-	9.95205
CGGCGCCATTTTCGAGT	mmu-mir-503	0	chrX	49298875	49304275	2419	-	9.2853
GCGCTTCATGTTTTGAA	mmu-mir-503	0	chrX	49298875	49304275	28	+	9.03749
GCCGGCCATTTCAAGAC	mmu-mir-503	0	chrX	49298875	49304275	727	-	9
TGCGATCATTTTCGCAA	mmu-mir-503	0	chrX	49298875	49304275	4836	-	8.9176
TGGGGCCATTTTGACCC	mmu-mir-503	0	chrX	49298875	49304275	1724	-	8.87392
TGAGTTCATTTTGTTAT	mmu-mir-504	20	chrX	55597651	55597851	112	-	9.23937
TTTCTCCATTTTCTCTC	mmu-mir-539	0	chr12	110043400	1.1E+08	5	-	9.78747
CGTCTTCATTTTTGCAC	mmu-mir-540	-10	chr12	109987400	1.1E+08	1539	-	11.4637
GGACCCCATTTTCACTA	mmu-mir-540	-10	chr12	109987400	1.1E+08	633	+	9.67413
TTTCTCCATTTTCTCTC	mmu-mir-541	0	chr12	110043400	1.1E+08	5	-	9.78747
GCTAGCCATATTGGTTT	mmu-mir-542	10	chrX	49296350	49298075	89	-	9.38398
AGCAGCCATTTTCTCAG	mmu-mir-542	10	chrX	49296350	49298075	1661	+	9.07849
TTTCTCCATTTTCTCTC	mmu-mir-543	0	chr12	110043400	1.1E+08	5	-	9.78747
TTTCTCCATTTTCTCTC	mmu-mir-544	0	chr12	110043400	1.1E+08	5	-	9.78747
TATTGCCATTCTTAGAT	mmu-mir-598	0	chr14	62559175	62563900	35	+	9.58798
TAAAGCCATGTTTACTA	mmu-mir-598	0	chr14	62559175	62563900	308	+	9.25856
CGCCGCCATCTTGACTC	mmu-mir-598	0	chr14	62559175	62563900	1456	-	8.99216
TTTCTCCATTTTCTCTC	mmu-mir-654	0	chr12	110043400	1.1E+08	5	-	9.78747
CGTCTTCATTTTTGCAC	mmu-mir-665	-10	chr12	109987400	1.1E+08	1539	-	11.4637
GGACCCCATTTTCACTA	mmu-mir-665	-10	chr12	109987400	1.1E+08	633	+	9.67413
TTTCTCCATTTTCTCTC	mmu-mir-666	0	chr12	110043400	1.1E+08	5	-	9.78747
CGTCTTCATTTTTGCAC	mmu-mir-673	-10	chr12	109987400	1.1E+08	1539	-	11.4637
GGACCCCATTTTCACTA	mmu-mir-673	-10	chr12	109987400	1.1E+08	633	+	9.67413
TGCCTCCATTTGGTGTC	mmu-mir-674	0	chr2	116827650	1.17E+08	483	+	8.80633

TTTCTCCATTTTCTCTC	mmu-mir-758	0	chr12	110043400	1.1E+08	5	-	9.78747
GAAAACCATTTTGTGTG	mmu-mir-760	22	chr3	122285704	1.22E+08	933	-	12.1904
TCTCTTCATATAGCAAC	mmu-mir-760	22	chr3	122285704	1.22E+08	1839	-	10.5729
ATTGGCCATTTTAAGAG	mmu-mir-760	22	chr3	122285704	1.22E+08	1251	-	8.96733
CGTCTTCATTTTTGCAC	mmu-mir-770	-10	chr12	109987400	1.1E+08	1539	-	11.4637
GGACCCCATTTTCACTA	mmu-mir-770	-10	chr12	109987400	1.1E+08	633	+	9.67413
GAGCTCCATTCAGGATC	mmu-mir-7a-2	0	chr7	78755900	78757275	1143	-	9.89473
GCTGTCCAGTTTCCGAC	mmu-mir-7b	6	chr17	55874475	55876575	994	-	10.0395
ACGATCCATCTTTAAAG	mmu-mir-802	10	chr16	93257500	93258650	476	-	9.72043
GGTTTCCATTTAAGCCT	mmu-mir-802	10	chr16	93257500	93258650	140	-	8.82451
TTTGACCATTTACATAC	mmu-mir-802	10	chr16	93257500	93258650	251	+	8.80627
TCTTTCCATTTTTTATT	mmu-mir-873	0	chr4	37072975	37074125	459	+	11.4564
AGTAGCCATGTTTGAAA	mmu-mir-873	0	chr4	37072975	37074125	157	-	10.0164
TGTTACCATCTATAATG	mmu-mir-874	0	chr13	58075200	58078100	108	+	10.0165
TACTTCCATTTTCAGAT	mmu-mir-875	0	chr15	35693875	35694875	369	-	11.1787
TCTTTCCATTTTTTATT	mmu-mir-876	0	chr4	37072975	37074125	459	+	11.4564
AGTAGCCATGTTTGAAA	mmu-mir-876	0	chr4	37072975	37074125	157	-	10.0164
GCTCTCCATGTAGGCTC	mmu-mir-877-1	20	chr17	35577703	35577903	146	+	11.12
TTTCTCCATTTTCTCTC	mmu-mir-882	0	chr12	110043400	1.1E+08	5	-	9.78747
CACAGCCATATATTCAC	mmu-mir-92a	0	chrX	48985925	48991150	711	+	9.75653
GAAACCCATTTTTTTTC	mmu-mir-92a	0	chrX	48985925	48991150	17	+	9.72013
GGTATCCATCTGTAAAC	mmu-mir-92a	0	chrX	48985925	48991150	395	-	8.92002
GGGTTCCATTTCCTAAT	mmu-mir-92a	0	chrX	48985925	48991150	3369	-	8.83589
GATGGCCATTTATGACT	mmu-mir-92b	20	chr3	89313125	89315675	2066	-	10.9679
TACCTCCATCTTGGCCC	mmu-mir-9-3	10	chr7	79377250	79382900	3209	+	9.69287
TGTTGCCATTTATTGGG	mmu-mir-9-3	10	chr7	79377250	79382900	2601	-	9.29362
CATCACCATTTCCCAGC	mmu-mir-9-3	10	chr7	79377250	79382900	1813	+	9.21554
ACTCTCCATCTACCGTA	mmu-mir-9-3	10	chr7	79377250	79382900	3488	-	9.01219
TCCCACCATTTTTGGCA	mmu-mir-96	10	chr6	30114875	30130825	1114	-	10.3837
GTTTTCCATTTTTGCAA	mmu-mir-96	10	chr6	30114875	30130825	12149	-	10.0079
TTCCTCCATTTTCTGAG	mmu-mir-96	10	chr6	30114875	30130825	14431	+	9.97098
GCTTGCCATCTACCAAG	mmu-mir-96	10	chr6	30114875	30130825	1503	+	9.74037
ACCCGCCATGTATCAAG	mmu-mir-96	10	chr6	30114875	30130825	13758	+	9.58357
GGAAATCATCTAGGGAG	mmu-mir-96	10	chr6	30114875	30130825	15201	+	9.29284
CCTCCCCATTTCGGAAA	mmu-mir-96	10	chr6	30114875	30130825	13537	-	9.25616
AGTGTCCATCTTACCAA	mmu-mir-96	10	chr6	30114875	30130825	5937	-	9.0865

Suppl. Table S1: Predicted promoter regions for mouse miRNAs.
Position weight matrix,YY1_02, was used to scan for YY1 motif.

Motif Sequence	miRNA ID	Promoter Score	Promoter Chr	Promoter Start	Promoter End	Relative Motif Location	Strand	Motif Search Score
GGACATCCATCTTGATTGTG	mmu-let-7a-1	10	chr13	48551775	48553275	676	+	8.89735
GGACATCCATCTTGATTGTG	mmu-let-7d	0	chr13	48551775	48553275	676	+	8.89735
TCTCCGCCCTCTTCCCGCCT	mmu-let-7e	20	chr17	17530726	17533550	2734	+	10.8153
TCTGAGCCAGGTTGGTTGGG	mmu-let-7e	20	chr17	17530726	17533550	2563	+	9.23549
CTTCAACCATTTTGCTAGCG	mmu-let-7e	20	chr17	17530726	17533550	551	-	8.95637
ACCACGCCTTCTTGGCGGAT	mmu-let-7e	20	chr17	17530726	17533550	155	+	8.84982
CTCGGGCCAGCTTCGGGGCA	mmu-let-7e	20	chr17	17530726	17533550	896	+	8.45257
TCTCTAGCATCTGGTCCCTT	mmu-let-7e	20	chr17	17530726	17533550	1568	-	8.12608
GGACATCCATCTTGATTGTG	mmu-let-7f-1	10	chr13	48551775	48553275	676	+	8.89735
GCGGCGGCATGCTGGCTCCG	mmu-let-7f-2	20	chrX	147143897	147144097	129	+	8.53692
TGTCCGCCATCTTGAAGGGA	mmu-let-7g	25	chr9	106028929	106029129	111	-	14.6747
CCGCCGCCATCTTTACAGCG	mmu-let-7i	10	chr10	122385250	122391850	4144	-	14.8881
CCGCCGCCATCTTACATCCG	mmu-let-7i	10	chr10	122385250	122391850	3967	+	13.4623
GCACAGCCATCAGGCATGGT	mmu-let-7i	10	chr10	122385250	122391850	6425	-	12.5499
ATTGAGCCATCTTTCCAGCA	mmu-let-7i	10	chr10	122385250	122391850	5932	-	11.5149
CCAGGGCCATGGTGTGGGAG	mmu-let-7i	10	chr10	122385250	122391850	3530	+	9.60934
TCTCAGGCATCTGTACTCAT	mmu-let-7i	10	chr10	122385250	122391850	6014	+	9.05302
CCCCCCCCATCATCCATCTT	mmu-let-7i	10	chr10	122385250	122391850	6087	+	8.68052
AGTAGGCCATCAATGTTTCA	mmu-let-7i	10	chr10	122385250	122391850	850	+	8.23598
TCTAGGCCATCTGTGCGGTA	mmu-mir-106a	0	chrX	48985925	48991150	1832	-	12.4464
TCTCCGCCATCTGTTTCTCG	mmu-mir-106a	0	chrX	48985925	48991150	931	+	9.48384
ACGATGCAATTTTGGTTGGT	mmu-mir-106a	0	chrX	48985925	48991150	433	-	9.07979
ATCCTGACATTTTGATTGCA	mmu-mir-106a	0	chrX	48985925	48991150	85	-	8.65137
TCCTGGCCCTCTGCTCTCCT	mmu-mir-106a	0	chrX	48985925	48991150	3115	+	8.52476
AGGCAGCCAAGTGGCCTCTA	mmu-mir-10a	14	chr11	96130725	96131975	500	+	7.90573
GCCTGGCCATCTGGGCCGAA	mmu-mir-122	0	chr18	65208050	65210300	1624	-	11.4389
GATGGGCCATGGTGACCAGG	mmu-mir-122	0	chr18	65208050	65210300	1005	-	8.30131
ACCAGGCCATCATCCATCCA	mmu-mir-124-1	0	chr14	63540450	63546275	5401	-	12.6306
TGTCGGCCTTCTACAATGGG	mmu-mir-124-1	0	chr14	63540450	63546275	4746	-	9.57304
GGGCGGCCAGGGTGGCTTCA	mmu-mir-124-1	0	chr14	63540450	63546275	1350	+	9.18477
CTTGGGCTATGTTGAATTCA	mmu-mir-124-1	0	chr14	63540450	63546275	375	-	9.18168

CCCCTGCAATCCGCACTCGA	mmu-mir-124-1	0	chr14	63540450	63546275	1929	+	8.36747
GGACGTCCATCTGGCCCCGG	mmu-mir-124-1	0	chr14	63540450	63546275	771	+	8.18191
CCTGAGCCAACTGGACTGTG	mmu-mir-124-3	15	chr2	180819000	180825000	1284	+	9.74205
GAGGGGCCCTCAGGGCTGCG	mmu-mir-124-3	15	chr2	180819000	180825000	4430	-	9.32194
CAAAGGCCATGCGCGCCGCA	mmu-mir-124-3	15	chr2	180819000	180825000	1233	-	8.64834
CTGAGGGCAGCTTGTCTCCT	mmu-mir-124-3	15	chr2	180819000	180825000	573	-	8.45033
TCTCCGCCCTCTTCCCGCCT	mmu-mir-125a	20	chr17	17530726	17533550	2734	+	10.8153
TCTGAGCCAGGTTGGTTGGG	mmu-mir-125a	20	chr17	17530726	17533550	2563	+	9.23549
CTTCAACCATTTTGCTAGCG	mmu-mir-125a	20	chr17	17530726	17533550	551	-	8.95637
ACCACGCCTTCTTGGCGGAT	mmu-mir-125a	20	chr17	17530726	17533550	155	+	8.84982
CTCGGGCCAGCTTCGGGGCA	mmu-mir-125a	20	chr17	17530726	17533550	896	+	8.45257
TCTCTAGCATCTGGTCCCTT	mmu-mir-125a	20	chr17	17530726	17533550	1568	-	8.12608
TCTCTCCCATCTTTTCGCCT	mmu-mir-125b-2	10	chr16	77525525	77528275	2601	-	10.473
TCCCCACCCTTTTCCCTGCT	mmu-mir-125b-2	10	chr16	77525525	77528275	1179	-	8.65981
CATCAGCCATCTTTAAGGGT	mmu-mir-127	-10	chr12	109987400	109991350	3278	-	12.0596
CCGCAGCCATCATGCGCAAT	mmu-mir-127	-10	chr12	109987400	109991350	2382	-	9.85884
CCTGAGCCATTTTCCAGACA	mmu-mir-127	-10	chr12	109987400	109991350	3234	-	8.71333
CCAGCGCCCTCTTGCGGCCA	mmu-mir-127	-10	chr12	109987400	109991350	2573	-	8.0899
TCTCCTCCATCTTGTTTCTT	mmu-mir-129-1	10	chr6	28970400	28972325	893	+	13.4019
CTGGAGCCAAGTTCTCTCAT	mmu-mir-129-1	10	chr6	28970400	28972325	1291	+	9.08646
GCGGTGCAATTTTGGTTGGA	mmu-mir-129-2	10	chr2	94041175	94045400	3549	-	10.0724
TGCCGTCCATCTTGAATACT	mmu-mir-130a	0	chr2	84542025	84546150	2553	+	11.7759
GAGCTGCCATGTGGAATTTT	mmu-mir-130a	0	chr2	84542025	84546150	3503	+	11.5058
TCTCTGCCATACTTGCTGCA	mmu-mir-130a	0	chr2	84542025	84546150	1789	+	11.0325
AGGCCTCCATTTTTCCTGCT	mmu-mir-130a	0	chr2	84542025	84546150	885	-	8.88695
TCAGAGCCATGCAGGCCTCT	mmu-mir-130a	0	chr2	84542025	84546150	719	+	8.0063
CCCGGGGCATCTGGGAACAG	mmu-mir-130a	0	chr2	84542025	84546150	2945	-	7.93422
CTGCCACCAGCTTCCCAGCT	mmu-mir-133b	0	chr1	20471175	20472525	234	+	8.73308
GCACTGCCAAGTAGTCTGAA	mmu-mir-135a-2	-10	chr10	91591125	91593550	2153	-	10.0037
CCACGGACATCAACAATGTT	mmu-mir-135a-2	-10	chr10	91591125	91593550	2197	+	8.71376
CATCAGCCATCTTTAAGGGT	mmu-mir-136	-10	chr12	109987400	109991350	3278	-	12.0596
CCGCAGCCATCATGCGCAAT	mmu-mir-136	-10	chr12	109987400	109991350	2382	-	9.85884
CCTGAGCCATTTTCCAGACA	mmu-mir-136	-10	chr12	109987400	109991350	3234	-	8.71333
CCAGCGCCCTCTTGCGGCCA	mmu-mir-136	-10	chr12	109987400	109991350	2573	-	8.0899
TTCCTGCCATCTAGGATGGT	mmu-mir-138-1	-10	chr9	122416550	122422625	3617	+	16.4157
GCTGTTCCATGCTCGCTCCA	mmu-mir-138-1	-10	chr9	122416550	122422625	3704	-	8.29093

CGCCCACCATCTTCGTCCCC	mmu-mir-138-1	-10	chr9	122416550	122422625	1766	-	7.99161
TTCCTGCCATGCTCAGACCT	mmu-mir-140	10	chr8	110434114	110434314	157	+	7.84586
AGGCAGCCATTTTGTCTCTT	mmu-mir-141	20	chr6	124683151	124685075	227	+	15.2025
CGGGGGCCATCTTTACCAGA	mmu-mir-141	20	chr6	124683151	124685075	378	+	12.5698
TGGCGGCCATGTTGCGTGGA	mmu-mir-142	0	chr11	87571775	87574325	703	-	16.459
TCACAACCATCTGTAATGAG	mmu-mir-142	0	chr11	87571775	87574325	2299	-	9.67131
CTGGCGCCATGTTGAGTCAC	mmu-mir-142	0	chr11	87571775	87574325	1098	+	9.33424
AAGAGGCCATCAGATCTCAT	mmu-mir-142	0	chr11	87571775	87574325	2284	+	8.466
CCCCCGCCATGGTCCACGGG	mmu-mir-143	20	chr18	61773267	61775675	208	+	8.767
TCGCTGCCTTCATGCTGGCT	mmu-mir-143	20	chr18	61773267	61775675	367	-	8.60234
TGCAGGACATCTTCTCCCTT	mmu-mir-143	20	chr18	61773267	61775675	1277	+	7.84728
CCCCCGCCATGGTCCACGGG	mmu-mir-145	20	chr18	61773267	61775675	208	+	8.767
TCGCTGCCTTCATGCTGGCT	mmu-mir-145	20	chr18	61773267	61775675	367	-	8.60234
TGCAGGACATCTTCTCCCTT	mmu-mir-145	20	chr18	61773267	61775675	1277	+	7.84728
CAGCAACCATCTTGACCTGT	mmu-mir-146a	0	chr11	43227200	43230425	2499	-	12.2679
AATGCGCCATTCGCTCTGCA	mmu-mir-146a	0	chr11	43227200	43230425	1062	+	9.7669
CTGCCGCCATCCAGGGAGAG	mmu-mir-146a	0	chr11	43227200	43230425	1318	+	9.50351
CTGCGGGCATCTGCATGGGT	mmu-mir-146a	0	chr11	43227200	43230425	1608	-	8.80057
TCTGGGCCATGTTTGATCAG	mmu-mir-146b	0	chr19	46390100	46393375	78	-	13.7285
CGGCGGCCATTTTTGTTTTG	mmu-mir-146b	0	chr19	46390100	46393375	1991	+	11.7863
TCCCTTCCATCTTGACTTTT	mmu-mir-148a	15	chr6	51198300	51201975	2179	+	12.061
AGGAAGCCATCTAGTCATGT	mmu-mir-148a	15	chr6	51198300	51201975	3185	-	9.80377
GCCCGGCCCTCTGCGCCCCG	mmu-mir-148a	15	chr6	51198300	51201975	2120	+	9.09341
CCCCAGCCATTCTCACCACA	mmu-mir-148a	15	chr6	51198300	51201975	1710	+	7.93455
GCGCCTCCATCTTGCCCGTA	mmu-mir-148b	25	chr15	103100832	103101032	115	-	11.278
CGGCGGCCAGGGAGCCTCGG	mmu-mir-149	10	chr1	94662155	94662355	154	+	7.8421
CCACGGCCTTCTGCATTTAA	mmu-mir-150	20	chr7	44988600	44989794	1126	-	8.97112
ATAAAACCATGATGCCTGGA	mmu-mir-150	20	chr7	44988600	44989794	578	+	7.96958
TGTCAGCCATGCACGTTCAG	mmu-mir-153	16	chr12	116927628	116927828	38	-	8.24669
TCTGTACCATCTGCATCTCT	mmu-mir-155	0	chr16	84584475	84588475	3158	-	8.48442
TCCCGGCCACCCTGCTGCCA	mmu-mir-155	0	chr16	84584475	84588475	1057	+	8.43936
GAGTGACCATGTTGGTTATA	mmu-mir-155	0	chr16	84584475	84588475	149	-	8.43914
GCTCAGCCATGCTCCTTAGG	mmu-mir-155	0	chr16	84584475	84588475	3637	+	8.39952
TCGGCGCCATTTTCGAGTGA	mmu-mir-15a	15	chr14	60602792	60602992	90	-	10.0759
CCACCGCCATCTTCGGGCCG	mmu-mir-15b	25	chr3	69092800	69093000	6	-	13.5425
TAGGCGCCATTTTCGAGTGA	mmu-mir-15b	25	chr3	69092800	69093000	110	+	8.92806

TCGGCGCCATTTTCGAGTGA	mmu-mir-16-1	15	chr14	60602792	60602992	90	-	10.0759
CCACCGCCATCTTCGGGCCG	mmu-mir-16-2	25	chr3	69092800	69093000	6	-	13.5425
TAGGCGCCATTTTCGAGTGA	mmu-mir-16-2	25	chr3	69092800	69093000	110	+	8.92806
CCGCCGCCATGTTCCTGCGG	mmu-mir-17	10	chr14	113921300	113927025	2440	+	12.1382
CCGCCACCATCTTCGCCAGC	mmu-mir-17	10	chr14	113921300	113927025	1708	-	9.48417
AGGCCACCATCAGTTTTGCA	mmu-mir-17	10	chr14	113921300	113927025	4596	-	9.20484
GCCCAGCCAACTGTCCTGTT	mmu-mir-17	10	chr14	113921300	113927025	4815	+	8.07844
TGCGGGCCATCTTGCAGACA	mmu-mir-181c	10	chr8	87069525	87071900	1037	+	12.718
TCCCGACCATTTAGAAGCCT	mmu-mir-181c	10	chr8	87069525	87071900	260	-	10.7779
ATTGTGCCCTGTGGTCTGCA	mmu-mir-181c	10	chr8	87069525	87071900	1741	+	9.04803
TGCGGGCCATCTTGCAGACA	mmu-mir-181d	10	chr8	87069525	87071900	1037	+	12.718
TCCCGACCATTTAGAAGCCT	mmu-mir-181d	10	chr8	87069525	87071900	260	-	10.7779
ATTGTGCCCTGTGGTCTGCA	mmu-mir-181d	10	chr8	87069525	87071900	1741	+	9.04803
GCGGGGCCATCTGGAACGAG	mmu-mir-182	10	chr6	30114875	30130825	9976	+	12.8263
CCTGGCCCATCTGGCTTACT	mmu-mir-182	10	chr6	30114875	30130825	4509	-	10.9442
GTTCTGCCATGGTTTCTCAG	mmu-mir-182	10	chr6	30114875	30130825	12375	+	9.98518
GTAGGGCCAGCTTCTGTGAT	mmu-mir-182	10	chr6	30114875	30130825	4346	-	9.48147
ATGGGGCCAGCTTGCCATCT	mmu-mir-182	10	chr6	30114875	30130825	1494	+	9.38086
TCAGTACCATGTGCACCGCA	mmu-mir-182	10	chr6	30114875	30130825	8569	-	9.28307
GGGCCTCCATCCTGCCTTCT	mmu-mir-182	10	chr6	30114875	30130825	5561	-	9.19071
GGTCCTCCATCTAGGATAGT	mmu-mir-182	10	chr6	30114875	30130825	394	+	9.13194
CAAGGGCCCTTTAGTTTGGT	mmu-mir-182	10	chr6	30114875	30130825	10356	-	8.57461
AGTGTACCATCTTTGGGGTT	mmu-mir-182	10	chr6	30114875	30130825	1874	-	8.35442
TGCCCGCCCTTTTGCCTACA	mmu-mir-182	10	chr6	30114875	30130825	6407	+	8.31798
CTGCGACCCTTTAGCATCCT	mmu-mir-182	10	chr6	30114875	30130825	13715	+	8.03552
CACTGACCATCTGGTAGCCA	mmu-mir-182	10	chr6	30114875	30130825	3656	-	7.98221
CCTCTGCCCTGTGCAATCTA	mmu-mir-182	10	chr6	30114875	30130825	11737	+	7.90795
TACCCGCCATGTATCAAGTT	mmu-mir-182	10	chr6	30114875	30130825	13757	+	7.87843
CCGGAGCCATCTGTCTGTAA	mmu-mir-182	10	chr6	30114875	30130825	10542	-	7.80862
GCGGGGCCATCTGGAACGAG	mmu-mir-183	10	chr6	30114875	30130825	9976	+	12.8263
CCTGGCCCATCTGGCTTACT	mmu-mir-183	10	chr6	30114875	30130825	4509	-	10.9442
GTTCTGCCATGGTTTCTCAG	mmu-mir-183	10	chr6	30114875	30130825	12375	+	9.98518
GTAGGGCCAGCTTCTGTGAT	mmu-mir-183	10	chr6	30114875	30130825	4346	-	9.48147
ATGGGGCCAGCTTGCCATCT	mmu-mir-183	10	chr6	30114875	30130825	1494	+	9.38086
TCAGTACCATGTGCACCGCA	mmu-mir-183	10	chr6	30114875	30130825	8569	-	9.28307
GGGCCTCCATCCTGCCTTCT	mmu-mir-183	10	chr6	30114875	30130825	5561	-	9.19071

GGTCCTCCATCTAGGATAGT	mmu-mir-183	10	chr6	30114875	30130825	394	+	9.13194
CAAGGGCCCTTTAGTTTGGT	mmu-mir-183	10	chr6	30114875	30130825	10356	-	8.57461
AGTGTACCATCTTTGGGGTT	mmu-mir-183	10	chr6	30114875	30130825	1874	-	8.35442
TGCCCGCCCTTTTGCCTACA	mmu-mir-183	10	chr6	30114875	30130825	6407	+	8.31798
CTGCGACCCTTTAGCATCCT	mmu-mir-183	10	chr6	30114875	30130825	13715	+	8.03552
CACTGACCATCTGGTAGCCA	mmu-mir-183	10	chr6	30114875	30130825	3656	-	7.98221
CCTCTGCCCTGTGCAATCTA	mmu-mir-183	10	chr6	30114875	30130825	11737	+	7.90795
TACCCGCCATGTATCAAGTT	mmu-mir-183	10	chr6	30114875	30130825	13757	+	7.87843
CCGGAGCCATCTGTCTGTAA	mmu-mir-183	10	chr6	30114875	30130825	10542	-	7.80862
TCCCGGCCATCTTCAGAGGA	mmu-mir-185	24	chr16	18261507	18261707	23	-	14.7492
CCGCCACCATCTGAGTACCT	mmu-mir-187	2	chr18	24608400	24611775	880	+	9.21919
TCCCGGACTTCTTTGCTGCA	mmu-mir-187	2	chr18	24608400	24611775	2258	-	8.68275
CAGACGCCGTCTTGGCAGAG	mmu-mir-188	10	chrX	6476159	6476359	101	-	8.27082
CCGCCGCCATGTTCCTGCGG	mmu-mir-18a	10	chr14	113921300	113927025	2440	+	12.1382
CCGCCACCATCTTCGCCAGC	mmu-mir-18a	10	chr14	113921300	113927025	1708	-	9.48417
AGGCCACCATCAGTTTTGCA	mmu-mir-18a	10	chr14	113921300	113927025	4596	-	9.20484
GCCCAGCCAACTGTCCTGTT	mmu-mir-18a	10	chr14	113921300	113927025	4815	+	8.07844
TCTAGGCCATCTGTGCGGTA	mmu-mir-18b	0	chrX	48985925	48991150	1832	-	12.4464
TCTCCGCCATCTGTTTCTCG	mmu-mir-18b	0	chrX	48985925	48991150	931	+	9.48384
ACGATGCAATTTTGGTTGGT	mmu-mir-18b	0	chrX	48985925	48991150	433	-	9.07979
ATCCTGACATTTTGATTGCA	mmu-mir-18b	0	chrX	48985925	48991150	85	-	8.65137
TCCTGGCCCTCTGCTCTCCT	mmu-mir-18b	0	chrX	48985925	48991150	3115	+	8.52476
TATAGGCCAGCTGGGCAGGA	mmu-mir-190	19	chr9	67338659	67338859	38	-	8.82883
GAGCCACCATGTGGTTTCTG	mmu-mir-190b	-15	chr3	90134875	90141050	5565	-	10.4126
CATCCACCATGTTTTCTGAC	mmu-mir-190b	-15	chr3	90134875	90141050	3922	+	8.78104
CCCCAACCACCTTCAATCTA	mmu-mir-190b	-15	chr3	90134875	90141050	2052	+	8.08829
TGTGGGCCAGTTTGGCCCCT	mmu-mir-191	20	chr9	108424326	108426775	1688	+	9.42867
CCGCTGCTATGTGGGCCAGT	mmu-mir-191	20	chr9	108424326	108426775	1679	+	8.09524
TTGGGGCCATCTTGCCTGAG	mmu-mir-192	-10	chr19	6247500	6249425	133	-	18.0559
CCTGGGCCATAGGCACTCCA	mmu-mir-192	-10	chr19	6247500	6249425	563	-	10.09
CTGCGCCCATCTGCGCCACA	mmu-mir-192	-10	chr19	6247500	6249425	743	+	9.37405
TTCCTACCATCTTGATAGAT	mmu-mir-193	10	chr11	79526175	79531800	3096	+	12.028
CCCCAGCCATGTCCTTTGAT	mmu-mir-193	10	chr11	79526175	79531800	25	+	9.992
CTGCTGCCTTCTGGAAGGCT	mmu-mir-193	10	chr11	79526175	79531800	4580	+	8.84215
GTGTGGCCATGTGCCCCGCT	mmu-mir-193	10	chr11	79526175	79531800	4939	+	8.37826
GCTCTGCCATTTAGACTGTA	mmu-mir-193b	15	chr16	13359750	13363900	347	-	14.685

CTTCTGCTATCTTTTTGCCT	mmu-mir-193b	15	chr16	13359750	13363900	3652	-	7.9639
CATGTGCCATGGTGCATGTA	mmu-mir-194-1	0	chr1	186908300	186912075	3029	+	11.5589
TCTAGGCCAGCTTGGGCCAA	mmu-mir-194-1	0	chr1	186908300	186912075	3713	-	8.53308
CAGCAGCCAGGTATGCTCCT	mmu-mir-194-1	0	chr1	186908300	186912075	2312	-	8.43335
CCTCTGCCAGCCACTCTACT	mmu-mir-194-1	0	chr1	186908300	186912075	3627	+	8.41585
TTGGGGCCATCTTGCCTGAG	mmu-mir-194-2	-10	chr19	6247500	6249425	133	-	18.0559
CCTGGGCCATAGGCACTCCA	mmu-mir-194-2	-10	chr19	6247500	6249425	563	-	10.09
CTGCGCCCATCTGCGCCACA	mmu-mir-194-2	-10	chr19	6247500	6249425	743	+	9.37405
TCAGGACCATCTTCCCAACA	mmu-mir-195	15	chr11	70048125	70050450	1065	+	12.2652
GGGGGACCATGGGGAATGGA	mmu-mir-195	15	chr11	70048125	70050450	2195	+	9.25983
TAAAGACCATTTTTGTTGCA	mmu-mir-196a-1	-10	chr11	96075625	96077750	1693	-	10.0558
CTGGGGCGATCTAGGAGGCT	mmu-mir-196b	10	chr6	52159743	52171275	3039	-	10.1428
TCCCAGCCATTTTTGTCGTA	mmu-mir-196b	10	chr6	52159743	52171275	5870	+	9.67438
ATGAGACCCTCTTTGCTGCT	mmu-mir-196b	10	chr6	52159743	52171275	10972	+	9.22902
TCCCTGCCATTTGCAGAGGT	mmu-mir-196b	10	chr6	52159743	52171275	6224	-	9.21019
TCTCAGCTATCTAGGGGGCT	mmu-mir-196b	10	chr6	52159743	52171275	8547	+	8.59015
CCAATGCCATGGTCTGTGAG	mmu-mir-196b	10	chr6	52159743	52171275	7570	-	8.41764
CAGCCACCATGTAGGAGAGA	mmu-mir-199a-1	10	chr9	21244900	21248875	907	+	8.73695
CTGAGGCCAGGGTCTCTCCT	mmu-mir-199a-1	10	chr9	21244900	21248875	784	-	8.11572
GCAGGGCTATCTTTGCTTTT	mmu-mir-199a-2	10	chr1	164052250	164061375	8718	-	9.97795
GCTGGCCCATCTTTTTTTTT	mmu-mir-199a-2	10	chr1	164052250	164061375	3242	+	8.98584
TAGCTACCATGTTGCTGTTT	mmu-mir-199a-2	10	chr1	164052250	164061375	8569	+	8.96291
CCACCACCATGATGTCCAAA	mmu-mir-199a-2	10	chr1	164052250	164061375	5490	+	8.52947
TTTGCACCATCATGGCCCCT	mmu-mir-199b	15	chr2	32138775	32141975	3016	-	11.4468
GAGGGGCCATGATGGTGCAA	mmu-mir-199b	15	chr2	32138775	32141975	3015	+	10.3535
GCCAAGCCATCTGGACCTCA	mmu-mir-199b	15	chr2	32138775	32141975	2904	-	9.45866
TCTGGTCCATCCAGGCAGCA	mmu-mir-199b	15	chr2	32138775	32141975	1678	-	9.26558
GCCCAGCCATTTGCTTTTTA	mmu-mir-199b	15	chr2	32138775	32141975	2065	-	9.09629
TCGGGGCCATGTGCTCACAC	mmu-mir-199b	15	chr2	32138775	32141975	927	+	8.86025
ATCCCACCCTCTTCCCTCAT	mmu-mir-199b	15	chr2	32138775	32141975	1984	-	8.47463
TGACTGCCATCGAGCTGCGG	mmu-mir-199b	15	chr2	32138775	32141975	1920	+	8.39946
CCGCCGCCATGTTCCTGCGG	mmu-mir-19a	10	chr14	113921300	113927025	2440	+	12.1382
CCGCCACCATCTTCGCCAGC	mmu-mir-19a	10	chr14	113921300	113927025	1708	-	9.48417
AGGCCACCATCAGTTTTGCA	mmu-mir-19a	10	chr14	113921300	113927025	4596	-	9.20484
GCCCAGCCAACTGTCCTGTT	mmu-mir-19a	10	chr14	113921300	113927025	4815	+	8.07844
TCTAGGCCATCTGTGCGGTA	mmu-mir-19b	0	chrX	48985925	48991150	1832	-	12.4464

TCTCCGCCATCTGTTTCTCG	mmu-mir-19b	0	chrX	48985925	48991150	931	+	9.48384
ACGATGCAATTTTGGTTGGT	mmu-mir-19b	0	chrX	48985925	48991150	433	-	9.07979
ATCCTGACATTTTGATTGCA	mmu-mir-19b	0	chrX	48985925	48991150	85	-	8.65137
TCCTGGCCCTCTGCTCTCCT	mmu-mir-19b	0	chrX	48985925	48991150	3115	+	8.52476
AGGCAGCCATTTTGTCTCTT	mmu-mir-200c	20	chr6	124683151	124685075	227	+	15.2025
CGGGGGCCATCTTTACCAGA	mmu-mir-200c	20	chr6	124683151	124685075	378	+	12.5698
AGGCTGCCATTATGGCACCA	mmu-mir-202	0	chr7	139821975	139823300	133	+	11.5366
CCTGGGCCTTGTGGACTGGG	mmu-mir-202	0	chr7	139821975	139823300	28	-	10.6446
CCTGGGCCAGCCTGTTTTGG	mmu-mir-202	0	chr7	139821975	139823300	8	-	8.52274
CTTGTGCCATGTACCAGGGA	mmu-mir-203	15	chr12	112577075	112579650	995	-	9.18554
CTTGGGCCAGCTGGCCAGAA	mmu-mir-203	15	chr12	112577075	112579650	795	+	9.0543
ACAGGGCCCTTTGGACTGCG	mmu-mir-203	15	chr12	112577075	112579650	968	+	8.83406
CCCAGGCCATCAGGCCTCTG	mmu-mir-205	0	chr1	195208350	195211700	906	+	12.4992
CCTCTCCCATCTAGCCCCCA	mmu-mir-205	0	chr1	195208350	195211700	890	+	10.7346
CAGGGGCCTTCAGGGCTGTG	mmu-mir-205	0	chr1	195208350	195211700	1731	+	9.3563
TCTGGGTCATGGAGGCTGTT	mmu-mir-205	0	chr1	195208350	195211700	2880	+	9.3177
CTGCCACCAGCTTCCCAGCT	mmu-mir-206	0	chr1	20471175	20472525	234	+	8.73308
CCGCCGCCATGTTCCTGCGG	mmu-mir-20a	10	chr14	113921300	113927025	2440	+	12.1382
CCGCCACCATCTTCGCCAGC	mmu-mir-20a	10	chr14	113921300	113927025	1708	-	9.48417
AGGCCACCATCAGTTTTGCA	mmu-mir-20a	10	chr14	113921300	113927025	4596	-	9.20484
GCCCAGCCAACTGTCCTGTT	mmu-mir-20a	10	chr14	113921300	113927025	4815	+	8.07844
TCTAGGCCATCTGTGCGGTA	mmu-mir-20b	0	chrX	48985925	48991150	1832	-	12.4464
TCTCCGCCATCTGTTTCTCG	mmu-mir-20b	0	chrX	48985925	48991150	931	+	9.48384
ACGATGCAATTTTGGTTGGT	mmu-mir-20b	0	chrX	48985925	48991150	433	-	9.07979
ATCCTGACATTTTGATTGCA	mmu-mir-20b	0	chrX	48985925	48991150	85	-	8.65137
TCCTGGCCCTCTGCTCTCCT	mmu-mir-20b	0	chrX	48985925	48991150	3115	+	8.52476
GCAGGGCTATCTTTGCTTTT	mmu-mir-214	10	chr1	164052250	164061375	8718	-	9.97795
GCTGGCCCATCTTTTTTTTT	mmu-mir-214	10	chr1	164052250	164061375	3242	+	8.98584
TAGCTACCATGTTGCTGTTT	mmu-mir-214	10	chr1	164052250	164061375	8569	+	8.96291
CCACCACCATGATGTCCAAA	mmu-mir-214	10	chr1	164052250	164061375	5490	+	8.52947
CATGTGCCATGGTGCATGTA	mmu-mir-215	0	chr1	186908300	186912075	3029	+	11.5589
TCTAGGCCAGCTTGGGCCAA	mmu-mir-215	0	chr1	186908300	186912075	3713	-	8.53308
CAGCAGCCAGGTATGCTCCT	mmu-mir-215	0	chr1	186908300	186912075	2312	-	8.43335
CCTCTGCCAGCCACTCTACT	mmu-mir-215	0	chr1	186908300	186912075	3627	+	8.41585
TCAGGGCCTTCTGCACCCAT	mmu-mir-216a	-10	chr11	28482175	28485250	2814	-	8.1458
TCAGGGCCTTCTGCACCCAT	mmu-mir-216b	-10	chr11	28482175	28485250	2814	-	8.1458

TCAGGGCCTTCTGCACCCAT	mmu-mir-217	-10	chr11	28482175	28485250	2814	-	8.1458
ACGGGGCCATCAATGGTCAT	mmu-mir-219-2	15	chr2	29666575	29669250	1849	+	11.3895
CCTCGTCCATCGGTGCTGCA	mmu-mir-219-2	15	chr2	29666575	29669250	2478	-	10.9057
CCTGTTCCATCTTGCCACCT	mmu-mir-220	0	chr6	136427625	136429100	143	-	11.6281
GTCCTGCCATCTACAGCTGT	mmu-mir-220	0	chr6	136427625	136429100	895	+	8.0946
TCTGAGCCATTCAGATTGTT	mmu-mir-221	-10	chrX	18319475	18326525	6619	-	9.37864
AAGCAGCCACCTACTATCCT	mmu-mir-221	-10	chrX	18319475	18326525	3420	+	9.01221
GCTCAGCCATTAAGACTAGG	mmu-mir-221	-10	chrX	18319475	18326525	5441	-	8.64092
CCACCCCCATCTTGCTCCTG	mmu-mir-221	-10	chrX	18319475	18326525	4672	+	8.59298
TGATTCCCATCTTGGCTGGG	mmu-mir-221	-10	chrX	18319475	18326525	4992	-	8.25768
TCTGAGCCATTCAGATTGTT	mmu-mir-222	-10	chrX	18319475	18326525	6619	-	9.37864
AAGCAGCCACCTACTATCCT	mmu-mir-222	-10	chrX	18319475	18326525	3420	+	9.01221
GCTCAGCCATTAAGACTAGG	mmu-mir-222	-10	chrX	18319475	18326525	5441	-	8.64092
CCACCCCCATCTTGCTCCTG	mmu-mir-222	-10	chrX	18319475	18326525	4672	+	8.59298
TGATTCCCATCTTGGCTGGG	mmu-mir-222	-10	chrX	18319475	18326525	4992	-	8.25768
CCGGTACCATCTTGGGGATT	mmu-mir-223	-10	chrX	92297875	92303675	4868	+	11.2268
ACCCGGCCATGAGTGATGTT	mmu-mir-223	-10	chrX	92297875	92303675	3774	+	10.8932
TCACTACAATCTTCTATCCT	mmu-mir-223	-10	chrX	92297875	92303675	5103	-	10.3922
CTTGAGCCATCATGCCTGGC	mmu-mir-223	-10	chrX	92297875	92303675	2711	+	10.3725
TCTGAGCCATCTTGGAAGTC	mmu-mir-223	-10	chrX	92297875	92303675	4635	+	10.2517
CCACTGCCCTCCTGTCGCCT	mmu-mir-223	-10	chrX	92297875	92303675	2455	+	9.67404
GGTCGGCCCTGGGGGCTCCA	mmu-mir-223	-10	chrX	92297875	92303675	1108	+	8.80151
GGGCTCCCATGTTGTATATT	mmu-mir-223	-10	chrX	92297875	92303675	2081	+	8.5167
TCTGGGCCAGCCTGCCTGTT	mmu-mir-23a	0	chr8	87086300	87095525	3562	+	11.8217
CTGGGAACATCTGCCCTCCA	mmu-mir-23a	0	chr8	87086300	87095525	5650	-	9.52752
GGGCGGCCATTTGGCCAGGC	mmu-mir-23a	0	chr8	87086300	87095525	7984	-	8.81359
AGGGGGCCATGAGGCCGTCT	mmu-mir-23a	0	chr8	87086300	87095525	3423	+	8.52869
TTGAGGCCAGCTTGGACTCT	mmu-mir-23a	0	chr8	87086300	87095525	3224	-	8.03959
TCTGGGCCAGCCTGCCTGTT	mmu-mir-24-2	0	chr8	87086300	87095525	3562	+	11.8217
CTGGGAACATCTGCCCTCCA	mmu-mir-24-2	0	chr8	87086300	87095525	5650	-	9.52752
GGGCGGCCATTTGGCCAGGC	mmu-mir-24-2	0	chr8	87086300	87095525	7984	-	8.81359
AGGGGGCCATGAGGCCGTCT	mmu-mir-24-2	0	chr8	87086300	87095525	3423	+	8.52869
TTGAGGCCAGCTTGGACTCT	mmu-mir-24-2	0	chr8	87086300	87095525	3224	-	8.03959
TCTGGGCCAGCCTGCCTGTT	mmu-mir-27a	0	chr8	87086300	87095525	3562	+	11.8217
CTGGGAACATCTGCCCTCCA	mmu-mir-27a	0	chr8	87086300	87095525	5650	-	9.52752
GGGCGGCCATTTGGCCAGGC	mmu-mir-27a	0	chr8	87086300	87095525	7984	-	8.81359

AGGGGGCCATGAGGCCGTCT	mmu-mir-27a	0	chr8	87086300	87095525	3423	+	8.52869
TTGAGGCCAGCTTGGACTCT	mmu-mir-27a	0	chr8	87086300	87095525	3224	-	8.03959
CTAGCGCCATCCTGACTCCA	mmu-mir-296	-15	chr2	173925825	173933325	4971	+	14.8045
CCAAGGCCATTTTCTGTGAA	mmu-mir-296	-15	chr2	173925825	173933325	5081	+	12.7673
TCTGCGCCATGAGCTATGCG	mmu-mir-296	-15	chr2	173925825	173933325	1303	+	9.94141
GATCCGACATCTTCCCAGCT	mmu-mir-296	-15	chr2	173925825	173933325	3664	-	9.55363
CACGTGCGATTTAGGCTCCA	mmu-mir-296	-15	chr2	173925825	173933325	3113	-	8.65742
CCTAGGCCATAGAGGCAGGA	mmu-mir-296	-15	chr2	173925825	173933325	3510	+	8.1068
CCGCGGCCAGGTTGGGGCGA	mmu-mir-297a-6	0	chr2	169051150	169052700	530	-	10.3263
CCCCTGCCAGCTTGCAGGGA	mmu-mir-297a-6	0	chr2	169051150	169052700	1017	+	10.0852
TCCATGCCATGCTGACCGCG	mmu-mir-297a-6	0	chr2	169051150	169052700	1110	+	9.02501
CCCCCCCCATCTACCTTCTG	mmu-mir-297a-6	0	chr2	169051150	169052700	1198	-	8.44448
ATGCAGCCATCGTGTCACTT	mmu-mir-297a-7	-10	chr10	42708700	42711800	604	-	12.4331
CCGGTGCCTTCTGCTTTACT	mmu-mir-297a-7	-10	chr10	42708700	42711800	2430	+	8.92733
TCAGAGCCATCTTTGAAAAG	mmu-mir-297a-7	-10	chr10	42708700	42711800	2865	-	8.38202
CTAGCGCCATCCTGACTCCA	mmu-mir-298	-15	chr2	173925825	173933325	4971	+	14.8045
CCAAGGCCATTTTCTGTGAA	mmu-mir-298	-15	chr2	173925825	173933325	5081	+	12.7673
TCTGCGCCATGAGCTATGCG	mmu-mir-298	-15	chr2	173925825	173933325	1303	+	9.94141
GATCCGACATCTTCCCAGCT	mmu-mir-298	-15	chr2	173925825	173933325	3664	-	9.55363
CACGTGCGATTTAGGCTCCA	mmu-mir-298	-15	chr2	173925825	173933325	3113	-	8.65742
CCTAGGCCATAGAGGCAGGA	mmu-mir-298	-15	chr2	173925825	173933325	3510	+	8.1068
GCAGGGCCAACCACTCTGCA	mmu-mir-29a	0	chr6	31006975	31008175	125	+	9.04598
GCAGGGCCAACCACTCTGCA	mmu-mir-29b-1	0	chr6	31006975	31008175	125	+	9.04598
AGACGGCCATTTTGGTTTGG	mmu-mir-29b-2	-10	chr1	196676100	196679875	3599	+	13.0742
CCGCCGCCCTTCTGTCTGAA	mmu-mir-29b-2	-10	chr1	196676100	196679875	883	-	9.77225
AACTGGCCATCAGGGCCCCT	mmu-mir-29b-2	-10	chr1	196676100	196679875	1563	+	8.43379
TGTCTACCATCCTGAAAACA	mmu-mir-29b-2	-10	chr1	196676100	196679875	3238	-	8.35306
CTGCGGCCAAGTTCGGCGTT	mmu-mir-29b-2	-10	chr1	196676100	196679875	925	-	8.08416
AGACGGCCATTTTGGTTTGG	mmu-mir-29c	-10	chr1	196676100	196679875	3599	+	13.0742
CCGCCGCCCTTCTGTCTGAA	mmu-mir-29c	-10	chr1	196676100	196679875	883	-	9.77225
AACTGGCCATCAGGGCCCCT	mmu-mir-29c	-10	chr1	196676100	196679875	1563	+	8.43379
TGTCTACCATCCTGAAAACA	mmu-mir-29c	-10	chr1	196676100	196679875	3238	-	8.35306
CTGCGGCCAAGTTCGGCGTT	mmu-mir-29c	-10	chr1	196676100	196679875	925	-	8.08416
CATCGGCCACCTTCCCTGGA	mmu-mir-30b	-8	chr15	68190175	68194550	3566	+	13.9397
AGTAGGCCATCTTGTTACTT	mmu-mir-30b	-8	chr15	68190175	68194550	907	-	13.1992
CTTAGGCCATGTACTCCACA	mmu-mir-30b	-8	chr15	68190175	68194550	3406	-	9.60096

CGGCCGCCATCTTGTTTGCG	mmu-mir-30c-1	14	chr4	120323146	120323346	157	-	18.0044
CGGCTGCTTTCTTCGCTGGA	mmu-mir-30c-1	14	chr4	120323146	120323346	35	-	8.27184
CGGCCGCCATCTTGTTTGCG	mmu-mir-30e	14	chr4	120323146	120323346	157	-	18.0044
CGGCTGCTTTCTTCGCTGGA	mmu-mir-30e	14	chr4	120323146	120323346	35	-	8.27184
GGTCGGCCATCGCGCCTCTG	mmu-mir-32	24	chr4	57041383	57041583	72	+	10.2592
CAGGTACCTTCTTGACTCCG	mmu-mir-320	2	chr14	69176250	69180100	1773	+	10.0314
GCGCTGCCATCTTACCCTGG	mmu-mir-320	2	chr14	69176250	69180100	2266	+	9.17421
GTGGGGCCATTTTGACCCGT	mmu-mir-322	0	chrX	49298875	49304275	1722	-	13.531
TAGCCGCCCTGTACCCTCCT	mmu-mir-322	0	chrX	49298875	49304275	3306	-	9.25215
TCGGCGCCATTTTCGAGTGG	mmu-mir-322	0	chrX	49298875	49304275	2417	-	9.10612
ACTCTACCCTCCTGTCTTCT	mmu-mir-322	0	chrX	49298875	49304275	4957	-	8.40585
GCGAGGACATCTGGGCGTCT	mmu-mir-322	0	chrX	49298875	49304275	4457	+	8.39377
CCCGGCCCTTCCTGGCTGCA	mmu-mir-322	0	chrX	49298875	49304275	3102	-	7.86191
TCCCCGCCATCCTCCTTGGG	mmu-mir-324	20	chr11	69816711	69816911	170	+	13.0294
AAGCAGACATCTGGGATGGG	mmu-mir-326	10	chr7	99425150	99427125	1445	+	9.25948
CCAGGGCCATATACATGGGT	mmu-mir-326	10	chr7	99425150	99427125	1471	-	8.462
TTCAGACCATCTTCTGTGGT	mmu-mir-327	-10	chr14	43909450	43912875	3188	-	12.6487
TAAGTGCCATTTGTTTTCCA	mmu-mir-327	-10	chr14	43909450	43912875	493	+	9.53377
GCCCAGCCATCAGCTCGGTG	mmu-mir-327	-10	chr14	43909450	43912875	1464	+	8.77448
TGCAGGCCATCTACTGCCTT	mmu-mir-328	-15	chr8	108213550	108217600	3743	+	9.3288
CCAGGGCCATCTGTAGGACA	mmu-mir-328	-15	chr8	108213550	108217600	3890	-	9.26496
TAACAGCCATCCAGTCCCTG	mmu-mir-328	-15	chr8	108213550	108217600	288	-	9.08194
CTAGTGACATTTGGGCTCAT	mmu-mir-328	-15	chr8	108213550	108217600	81	+	8.10795
GAGCAGCCATCTTCTGGGGC	mmu-mir-328	-15	chr8	108213550	108217600	1563	+	8.04589
CCGCCGCCATGTTTGAGCTG	mmu-mir-33	17	chr15	81974358	81974558	156	-	11.2738
CTCCGGCCATGGTGGTTTCA	mmu-mir-331	0	chr10	93443775	93445175	1068	-	13.2724
CTTCTGCCATCTTTGACTAA	mmu-mir-331	0	chr10	93443775	93445175	918	-	10.9215
ATGCGGCCATCTCCATGGAG	mmu-mir-331	0	chr10	93443775	93445175	1243	-	8.51589
AGAAAGCCAGCTTGTTTGGA	mmu-mir-335	9	chr6	30683456	30683656	121	+	8.14379
ACCCGCCCATCCTGCCGCAG	mmu-mir-335	25	chr6	30687962	30688162	28	-	7.84579
CATCAGCCATCTTTAAGGGT	mmu-mir-337	-10	chr12	109987400	109991350	3278	-	12.0596
CCGCAGCCATCATGCGCAAT	mmu-mir-337	-10	chr12	109987400	109991350	2382	-	9.85884
CCTGAGCCATTTTCCAGACA	mmu-mir-337	-10	chr12	109987400	109991350	3234	-	8.71333
CCAGCGCCCTCTTGCGGCCA	mmu-mir-337	-10	chr12	109987400	109991350	2573	-	8.0899
CTTTGGCCATCTTGGCTAGT	mmu-mir-339	10	chr5	139624300	139627075	2466	+	15.0021
AGGCGTCCATCTTCCAGCCA	mmu-mir-339	22	chr5	139714076	139714276	141	+	9.59727

CAGCCGCCATGAAGTGAGAT	mmu-mir-343	20	chr7	18540460	18540660	108	+	8.64167
GCAGGGCCATCCATGCTCAG	mmu-mir-34a	0	chr4	148890450	148895975	3608	+	11.4459
CCCGGGCCATCGCGATTCTT	mmu-mir-34a	0	chr4	148890450	148895975	1997	-	10.5455
TCTGGAGCATCCTGGCTGAG	mmu-mir-34a	0	chr4	148890450	148895975	1308	+	9.59151
CCTGGGCCTTCTTGCACTCT	mmu-mir-34a	0	chr4	148890450	148895975	3485	-	9.55589
ACAGGGACATTTTTGCTTCT	mmu-mir-34a	0	chr4	148890450	148895975	4644	+	8.22259
TCTGCGCCATCTTCACTAAT	mmu-mir-34b	20	chr9	50855701	50858400	1473	+	15.5025
GCGCGGCCCTGCTGGGTGTG	mmu-mir-34b	20	chr9	50855701	50858400	284	-	8.25789
AAGGTGCCTTCCTGGATCTA	mmu-mir-34b	20	chr9	50855701	50858400	2559	-	7.97705
TCTGCGCCATCTTCACTAAT	mmu-mir-34c	20	chr9	50855701	50858400	1473	+	15.5025
GCGCGGCCCTGCTGGGTGTG	mmu-mir-34c	20	chr9	50855701	50858400	284	-	8.25789
AAGGTGCCTTCCTGGATCTA	mmu-mir-34c	20	chr9	50855701	50858400	2559	-	7.97705
GGAATGCCATCTGGTTTGGG	mmu-mir-350	0	chr1	178636600	178640175	1451	-	11.7479
CCAGTGGCATTTTGTTTGAT	mmu-mir-350	0	chr1	178636600	178640175	557	-	9.55732
CCTGCGCCATCTGGCTGCTT	mmu-mir-351	10	chrX	49296350	49298075	846	-	12.7211
AAGCAGCCATTTTCTCAGAG	mmu-mir-351	10	chrX	49296350	49298075	1660	+	10.447
TATCCGCCATCTTGACAGGA	mmu-mir-361	20	chrX	109302359	109302559	64	+	16.1693
CAGACGCCGTCTTGGCAGAG	mmu-mir-362	10	chrX	6476159	6476359	101	-	8.27082
TCTAGGCCATCTGTGCGGTA	mmu-mir-363	0	chrX	48985925	48991150	1832	-	12.4464
TCTCCGCCATCTGTTTCTCG	mmu-mir-363	0	chrX	48985925	48991150	931	+	9.48384
ACGATGCAATTTTGGTTGGT	mmu-mir-363	0	chrX	48985925	48991150	433	-	9.07979
ATCCTGACATTTTGATTGCA	mmu-mir-363	0	chrX	48985925	48991150	85	-	8.65137
TCCTGGCCCTCTGCTCTCCT	mmu-mir-363	0	chrX	48985925	48991150	3115	+	8.52476
GCTCTGCCATTTAGACTGTA	mmu-mir-365-1	6	chr16	13359750	13363900	347	-	14.685
CTTCTGCTATCTTTTTGCCT	mmu-mir-365-1	6	chr16	13359750	13363900	3652	-	7.9639
TTCCTACCATCTTGATAGAT	mmu-mir-365-2	0	chr11	79526175	79531800	3096	+	12.028
CCCCAGCCATGTCCTTTGAT	mmu-mir-365-2	0	chr11	79526175	79531800	25	+	9.992
CTGCTGCCTTCTGGAAGGCT	mmu-mir-365-2	0	chr11	79526175	79531800	4580	+	8.84215
GTGTGGCCATGTGCCCCGCT	mmu-mir-365-2	0	chr11	79526175	79531800	4939	+	8.37826
GCTCAACCATTTTGTTGGCT	mmu-mir-374	0	chrX	99817575	99820975	2247	-	11.0264
TCCCCGCCATCTTCCAGCCG	mmu-mir-378	35	chr18	61525700	61525900	26	+	13.101
ATTCAGCCATCTTCCTGGAT	mmu-mir-384	-10	chrX	101592850	101595950	1377	+	11.9558
TGGCCGCCATCCTCCCTTTG	mmu-mir-384	-10	chrX	101592850	101595950	316	+	11.6522
ATCAGACCATCTTTCCACAA	mmu-mir-384	-10	chrX	101592850	101595950	2219	+	8.71162
AGTCGGCCATTTTATACCCT	mmu-mir-421	0	chrX	99822432	99826775	1294	+	8.96797
TTGATACCATGTGGTCAGAT	mmu-mir-421	0	chrX	99822432	99826775	232	-	8.85867

GGATCGCCATCTTGTTCCCG	mmu-mir-423	35	chr11	76894530	76894730	53	+	8.32256
TGTGGGCCAGTTTGGCCCCT	mmu-mir-425	20	chr9	108424326	108426775	1688	+	9.42867
CCGCTGCTATGTGGGCCAGT	mmu-mir-425	20	chr9	108424326	108426775	1679	+	8.09524
CATCAGCCATCTTTAAGGGT	mmu-mir-431	-10	chr12	109987400	109991350	3278	-	12.0596
CCGCAGCCATCATGCGCAAT	mmu-mir-431	-10	chr12	109987400	109991350	2382	-	9.85884
CCTGAGCCATTTTCCAGACA	mmu-mir-431	-10	chr12	109987400	109991350	3234	-	8.71333
CCAGCGCCCTCTTGCGGCCA	mmu-mir-431	-10	chr12	109987400	109991350	2573	-	8.0899
CATCAGCCATCTTTAAGGGT	mmu-mir-433	-10	chr12	109987400	109991350	3278	-	12.0596
CCGCAGCCATCATGCGCAAT	mmu-mir-433	-10	chr12	109987400	109991350	2382	-	9.85884
CCTGAGCCATTTTCCAGACA	mmu-mir-433	-10	chr12	109987400	109991350	3234	-	8.71333
CCAGCGCCCTCTTGCGGCCA	mmu-mir-433	-10	chr12	109987400	109991350	2573	-	8.0899
CATCAGCCATCTTTAAGGGT	mmu-mir-434	-10	chr12	109987400	109991350	3278	-	12.0596
CCGCAGCCATCATGCGCAAT	mmu-mir-434	-10	chr12	109987400	109991350	2382	-	9.85884
CCTGAGCCATTTTCCAGACA	mmu-mir-434	-10	chr12	109987400	109991350	3234	-	8.71333
CCAGCGCCCTCTTGCGGCCA	mmu-mir-434	-10	chr12	109987400	109991350	2573	-	8.0899
CAGCAGCCAGGTTCTCTGCT	mmu-mir-449a	15	chr13	114154825	114157525	384	-	11.4067
GCTGAGCCATCTCTCCAGCT	mmu-mir-449a	15	chr13	114154825	114157525	2480	+	8.03814
AGTGAACCATCTTTGCATGA	mmu-mir-449a	15	chr13	114154825	114157525	27	-	8.03034
ATAGTGCCATCTGGTGGATA	mmu-mir-449a	15	chr13	114154825	114157525	2156	+	7.97764
CAGCAGCCAGGTTCTCTGCT	mmu-mir-449b	15	chr13	114154825	114157525	384	-	11.4067
GCTGAGCCATCTCTCCAGCT	mmu-mir-449b	15	chr13	114154825	114157525	2480	+	8.03814
AGTGAACCATCTTTGCATGA	mmu-mir-449b	15	chr13	114154825	114157525	27	-	8.03034
ATAGTGCCATCTGGTGGATA	mmu-mir-449b	15	chr13	114154825	114157525	2156	+	7.97764
CCTGCGCCATCTGGCTGCTT	mmu-mir-450a-1	0	chrX	49296350	49298075	846	-	12.7211
AAGCAGCCATTTTCTCAGAG	mmu-mir-450a-1	0	chrX	49296350	49298075	1660	+	10.447
CCTGCGCCATCTGGCTGCTT	mmu-mir-450a-2	0	chrX	49296350	49298075	846	-	12.7211
AAGCAGCCATTTTCTCAGAG	mmu-mir-450a-2	0	chrX	49296350	49298075	1660	+	10.447
CCTGCGCCATCTGGCTGCTT	mmu-mir-450b	0	chrX	49296350	49298075	846	-	12.7211
AAGCAGCCATTTTCTCAGAG	mmu-mir-450b	0	chrX	49296350	49298075	1660	+	10.447
TCGCGGCCCTGCAGCCTCGT	mmu-mir-484	2	chr16	14070875	14074575	2313	+	9.93334
TCCAGACCAGCTACGTTGCT	mmu-mir-484	2	chr16	14070875	14074575	1772	+	8.22156
CATCAGCCATCTTTAAGGGT	mmu-mir-493	-10	chr12	109987400	109991350	3278	-	12.0596
CCGCAGCCATCATGCGCAAT	mmu-mir-493	-10	chr12	109987400	109991350	2382	-	9.85884
CCTGAGCCATTTTCCAGACA	mmu-mir-493	-10	chr12	109987400	109991350	3234	-	8.71333
CCAGCGCCCTCTTGCGGCCA	mmu-mir-493	-10	chr12	109987400	109991350	2573	-	8.0899
TCAGGACCATCTTCCCAACA	mmu-mir-497	15	chr11	70048125	70050450	1065	+	12.2652

GGGGGACCATGGGGAATGGA	mmu-mir-497	15	chr11	70048125	70050450	2195	+	9.25983
CAGACGCCGTCTTGGCAGAG	mmu-mir-500	10	chrX	6476159	6476359	101	-	8.27082
CAGACGCCGTCTTGGCAGAG	mmu-mir-501	10	chrX	6476159	6476359	101	-	8.27082
GTGGGGCCATTTTGACCCGT	mmu-mir-503	0	chrX	49298875	49304275	1722	-	13.531
TAGCCGCCCTGTACCCTCCT	mmu-mir-503	0	chrX	49298875	49304275	3306	-	9.25215
TCGGCGCCATTTTCGAGTGG	mmu-mir-503	0	chrX	49298875	49304275	2417	-	9.10612
ACTCTACCCTCCTGTCTTCT	mmu-mir-503	0	chrX	49298875	49304275	4957	-	8.40585
GCGAGGACATCTGGGCGTCT	mmu-mir-503	0	chrX	49298875	49304275	4457	+	8.39377
CCCGGCCCTTCCTGGCTGCA	mmu-mir-503	0	chrX	49298875	49304275	3102	-	7.86191
CGCCTGCCCTCCTGCCGCCT	mmu-mir-505	20	chrX	56750496	56750696	87	+	7.81687
CAGACGCCGTCTTGGCAGAG	mmu-mir-532	10	chrX	6476159	6476359	101	-	8.27082
CATCAGCCATCTTTAAGGGT	mmu-mir-540	-10	chr12	109987400	109991350	3278	-	12.0596
CCGCAGCCATCATGCGCAAT	mmu-mir-540	-10	chr12	109987400	109991350	2382	-	9.85884
CCTGAGCCATTTTCCAGACA	mmu-mir-540	-10	chr12	109987400	109991350	3234	-	8.71333
CCAGCGCCCTCTTGCGGCCA	mmu-mir-540	-10	chr12	109987400	109991350	2573	-	8.0899
CCTGCGCCATCTGGCTGCTT	mmu-mir-542	10	chrX	49296350	49298075	846	-	12.7211
AAGCAGCCATTTTCTCAGAG	mmu-mir-542	10	chrX	49296350	49298075	1660	+	10.447
CCGCTGCCATTCTGCATGGA	mmu-mir-568	-10	chr16	43528050	43530275	1159	-	13.8517
GAAATGCCATCAGCTATGTA	mmu-mir-568	-10	chr16	43528050	43530275	546	+	8.39733
GGGCGGCCATCTTGGCCGGT	mmu-mir-598	0	chr14	62559175	62563900	1215	+	18.4888
TCGCCGCCATCTTGACTCTC	mmu-mir-598	0	chr14	62559175	62563900	1454	-	15.4325
ACACAGCCATTTTGGTGGGG	mmu-mir-598	0	chr14	62559175	62563900	832	+	11.2783
CATCAGCCATCTTTAAGGGT	mmu-mir-665	-10	chr12	109987400	109991350	3278	-	12.0596
CCGCAGCCATCATGCGCAAT	mmu-mir-665	-10	chr12	109987400	109991350	2382	-	9.85884
CCTGAGCCATTTTCCAGACA	mmu-mir-665	-10	chr12	109987400	109991350	3234	-	8.71333
CCAGCGCCCTCTTGCGGCCA	mmu-mir-665	-10	chr12	109987400	109991350	2573	-	8.0899
CATCAGCCATCTTTAAGGGT	mmu-mir-673	-10	chr12	109987400	109991350	3278	-	12.0596
CCGCAGCCATCATGCGCAAT	mmu-mir-673	-10	chr12	109987400	109991350	2382	-	9.85884
CCTGAGCCATTTTCCAGACA	mmu-mir-673	-10	chr12	109987400	109991350	3234	-	8.71333
CCAGCGCCCTCTTGCGGCCA	mmu-mir-673	-10	chr12	109987400	109991350	2573	-	8.0899
TATGTACCATCTATTTGGCT	mmu-mir-674	0	chr2	116827650	116829025	1129	+	8.63538
TGAAAACCATTTTGTGTGCA	mmu-mir-760	22	chr3	122285704	122287675	931	-	9.00205
CATCAGCCATCTTTAAGGGT	mmu-mir-770	-10	chr12	109987400	109991350	3278	-	12.0596
CCGCAGCCATCATGCGCAAT	mmu-mir-770	-10	chr12	109987400	109991350	2382	-	9.85884
CCTGAGCCATTTTCCAGACA	mmu-mir-770	-10	chr12	109987400	109991350	3234	-	8.71333
CCAGCGCCCTCTTGCGGCCA	mmu-mir-770	-10	chr12	109987400	109991350	2573	-	8.0899

TCCCGGGCATCCTGGGACTT	mmu-mir-7a-2	0	chr7	78755900	78757275	602	-	8.03257
AGAAGGCCAACTGGACTGTT	mmu-mir-7b	6	chr17	55874475	55876575	1131	+	9.38597
GATGTGCCAACTTGCATTGT	mmu-mir-7b	6	chr17	55874475	55876575	1107	-	8.50599
CAGCGGCCAGCATCCATGCT	mmu-mir-874	0	chr13	58075200	58078100	1809	-	10.832
TCTAGGCCATCTGTGCGGTA	mmu-mir-92a	0	chrX	48985925	48991150	1832	-	12.4464
TCTCCGCCATCTGTTTCTCG	mmu-mir-92a	0	chrX	48985925	48991150	931	+	9.48384
ACGATGCAATTTTGGTTGGT	mmu-mir-92a	0	chrX	48985925	48991150	433	-	9.07979
ATCCTGACATTTTGATTGCA	mmu-mir-92a	0	chrX	48985925	48991150	85	-	8.65137
TCCTGGCCCTCTGCTCTCCT	mmu-mir-92a	0	chrX	48985925	48991150	3115	+	8.52476
CCCCGGGCATTCGGGCTCCT	mmu-mir-92b	20	chr3	89313125	89315675	1925	+	9.83856
CCATCGCCATGGTGACGGGT	mmu-mir-92b	20	chr3	89313125	89315675	240	+	7.9488
ACTGGACCATCAGCCCTCCT	mmu-mir-9-3	10	chr7	79377250	79382900	1194	-	12.5244
GTGCTACCATCCTGACTTGA	mmu-mir-9-3	10	chr7	79377250	79382900	3072	+	11.9055
TACCGGCCATATGGGCCTCA	mmu-mir-9-3	10	chr7	79377250	79382900	4736	+	9.47711
TTACCTCCATCTTGGCCCAG	mmu-mir-9-3	10	chr7	79377250	79382900	3208	+	9.47018
CCCCACCCATCCTGTCCCAT	mmu-mir-9-3	10	chr7	79377250	79382900	4405	+	8.24129
GCGGGGCCATCTGGAACGAG	mmu-mir-96	10	chr6	30114875	30130825	9976	+	12.8263
CCTGGCCCATCTGGCTTACT	mmu-mir-96	10	chr6	30114875	30130825	4509	-	10.9442
GTTCTGCCATGGTTTCTCAG	mmu-mir-96	10	chr6	30114875	30130825	12375	+	9.98518
GTAGGGCCAGCTTCTGTGAT	mmu-mir-96	10	chr6	30114875	30130825	4346	-	9.48147
ATGGGGCCAGCTTGCCATCT	mmu-mir-96	10	chr6	30114875	30130825	1494	+	9.38086
TCAGTACCATGTGCACCGCA	mmu-mir-96	10	chr6	30114875	30130825	8569	-	9.28307
GGGCCTCCATCCTGCCTTCT	mmu-mir-96	10	chr6	30114875	30130825	5561	-	9.19071
GGTCCTCCATCTAGGATAGT	mmu-mir-96	10	chr6	30114875	30130825	394	+	9.13194
CAAGGGCCCTTTAGTTTGGT	mmu-mir-96	10	chr6	30114875	30130825	10356	-	8.57461
AGTGTACCATCTTTGGGGTT	mmu-mir-96	10	chr6	30114875	30130825	1874	-	8.35442
TGCCCGCCCTTTTGCCTACA	mmu-mir-96	10	chr6	30114875	30130825	6407	+	8.31798
CTGCGACCCTTTAGCATCCT	mmu-mir-96	10	chr6	30114875	30130825	13715	+	8.03552
CACTGACCATCTGGTAGCCA	mmu-mir-96	10	chr6	30114875	30130825	3656	-	7.98221
CCTCTGCCCTGTGCAATCTA	mmu-mir-96	10	chr6	30114875	30130825	11737	+	7.90795
TACCCGCCATGTATCAAGTT	mmu-mir-96	10	chr6	30114875	30130825	13757	+	7.87843
CCGGAGCCATCTGTCTGTAA	mmu-mir-96	10	chr6	30114875	30130825	10542	-	7.80862
TCTCCGCCCTCTTCCCGCCT	mmu-mir-99b	20	chr17	17530726	17533550	2734	+	10.8153
TCTGAGCCAGGTTGGTTGGG	mmu-mir-99b	20	chr17	17530726	17533550	2563	+	9.23549
CTTCAACCATTTTGCTAGCG	mmu-mir-99b	20	chr17	17530726	17533550	551	-	8.95637
ACCACGCCTTCTTGGCGGAT	mmu-mir-99b	20	chr17	17530726	17533550	155	+	8.84982

CTCGGGCCAGCTTCGGGGCA	mmu-mir-99b	20	chr17	17530726	17533550	896	+	8.45257
TCTCTAGCATCTGGTCCCTT	mmu-mir-99b	20	chr17	17530726	17533550	1568	-	8.12608

Suppl. Table S1: Predicted promoter regions for mouse miRNAs.
Position weight matrix,YY1_Q6, was used to scan for YY1 motif.

Motif Sequence	miRNA ID	Promoter Score	Promoter Chr	Promoter Start	Promoter End	Relative Motif Location	Strand	Motif Search Score
GCCATTTTT	mmu-let-7a-1	10	chr13	48551775	48553275	570	-	12.2387
TCCATCTTG	mmu-let-7a-1	10	chr13	48551775	48553275	681	+	11.2215
GCCATCTCT	mmu-let-7a-1	10	chr13	48551775	48553275	1140	-	10.3619
ACCATCTTG	mmu-let-7a-1	10	chr13	48551775	48553275	96	-	10.3187
ACCATCTTT	mmu-let-7a-2	8	chr9	41225884	41226084	158	+	9.85665
GCCATTTTT	mmu-let-7d	0	chr13	48551775	48553275	570	-	12.2387
TCCATCTTG	mmu-let-7d	0	chr13	48551775	48553275	681	+	11.2215
GCCATCTCT	mmu-let-7d	0	chr13	48551775	48553275	1140	-	10.3619
ACCATCTTG	mmu-let-7d	0	chr13	48551775	48553275	96	-	10.3187
ACCATTTTG	mmu-let-7e	20	chr17	17530726	17533550	557	-	9.81414
GCCATTTTT	mmu-let-7f-1	10	chr13	48551775	48553275	570	-	12.2387
TCCATCTTG	mmu-let-7f-1	10	chr13	48551775	48553275	681	+	11.2215
GCCATCTCT	mmu-let-7f-1	10	chr13	48551775	48553275	1140	-	10.3619
ACCATCTTG	mmu-let-7f-1	10	chr13	48551775	48553275	96	-	10.3187
GCCATCTTG	mmu-let-7g	25	chr9	106028929	106029129	117	-	13.2054
GCCATCTTT	mmu-let-7i	10	chr10	122385250	122391850	5938	-	12.7433
GCCATCTTT	mmu-let-7i	10	chr10	122385250	122391850	4150	-	12.7433
GCCATCTTA	mmu-let-7i	10	chr10	122385250	122391850	3972	+	11.0695
TCCATCTTT	mmu-let-7i	10	chr10	122385250	122391850	6099	+	10.7594
GCCATGGTG	mmu-let-7i	10	chr10	122385250	122391850	3535	+	9.60737
GCCATCTCC	mmu-let-7i	10	chr10	122385250	122391850	1500	-	9.53163
GCCATCTAG	mmu-let-7i	10	chr10	122385250	122391850	329	-	9.51535
ACCATCTTT	mmu-mir-100	8	chr9	41225884	41226084	158	+	9.85665
GCCATCTGT	mmu-mir-106a	0	chrX	48985925	48991150	936	+	10.3844
GCCATCTGT	mmu-mir-106a	0	chrX	48985925	48991150	1838	-	10.3844
GCCATCTGG	mmu-mir-122	0	chr18	65208050	65210300	1630	-	10.8465
GCCATGGTG	mmu-mir-122	0	chr18	65208050	65210300	1011	-	9.60737
GCTATTTTG	mmu-mir-122	0	chr18	65208050	65210300	243	+	9.52151
GCCATTTCT	mmu-mir-124-1	0	chr14	63540450	63546275	224	-	9.85738
GCTATGTTG	mmu-mir-124-1	0	chr14	63540450	63546275	381	-	9.4844
ACCATTTTG	mmu-mir-125a	20	chr17	17530726	17533550	557	-	9.81414

ACCATCTTT	mmu-mir-125b-1	7	chr9	41225884	41226084	158	+	9.85665
TCCATCTTT	mmu-mir-125b-2	10	chr16	77525525	77528275	1767	+	10.7594
GCCATATTC	mmu-mir-125b-2	10	chr16	77525525	77528275	1912	+	9.94887
GCCATCTCT	mmu-mir-126	19	chr2	26402991	26403191	17	+	10.3619
GCCATCTTT	mmu-mir-127	-10	chr12	109987400	109991350	1969	+	12.7433
GCCATCTTT	mmu-mir-127	-10	chr12	109987400	109991350	3284	-	12.7433
GCCATTTTC	mmu-mir-127	-10	chr12	109987400	109991350	3240	-	11.4084
GCCATCTCT	mmu-mir-127	-10	chr12	109987400	109991350	2771	-	10.3619
GCCATCATG	mmu-mir-127	-10	chr12	109987400	109991350	2388	-	10.1993
GCCATGTCT	mmu-mir-127	-10	chr12	109987400	109991350	1896	-	9.82026
GCCATCTGC	mmu-mir-127	-10	chr12	109987400	109991350	1034	+	9.55408
TCCATCTTG	mmu-mir-129-1	10	chr6	28970400	28972325	898	+	11.2215
TCCATCTTG	mmu-mir-130a	0	chr2	84542025	84546150	2558	+	11.2215
GCCATTTCG	mmu-mir-130a	0	chr2	84542025	84546150	513	-	10.3194
GCCATGTGG	mmu-mir-130a	0	chr2	84542025	84546150	3508	+	10.3048
TCCATTTTT	mmu-mir-130a	0	chr2	84542025	84546150	891	-	10.2548
GCCATCTGC	mmu-mir-130a	0	chr2	84542025	84546150	2037	-	9.55408
TCCATCTTT	mmu-mir-130b	30	chr16	17038569	17038769	114	+	10.7594
GCTATCTTT	mmu-mir-134	0	chr12	110043400	110045125	868	+	9.56401
TCCATTTTC	mmu-mir-134	0	chr12	110043400	110045125	9	-	9.42452
GCTATCTTT	mmu-mir-135a-2	-10	chr10	91591125	91593550	1528	-	9.56401
GCCATCTTT	mmu-mir-136	-10	chr12	109987400	109991350	1969	+	12.7433
GCCATCTTT	mmu-mir-136	-10	chr12	109987400	109991350	3284	-	12.7433
GCCATTTTC	mmu-mir-136	-10	chr12	109987400	109991350	3240	-	11.4084
GCCATCTCT	mmu-mir-136	-10	chr12	109987400	109991350	2771	-	10.3619
GCCATCATG	mmu-mir-136	-10	chr12	109987400	109991350	2388	-	10.1993
GCCATGTCT	mmu-mir-136	-10	chr12	109987400	109991350	1896	-	9.82026
GCCATCTGC	mmu-mir-136	-10	chr12	109987400	109991350	1034	+	9.55408
GCCATTTGG	mmu-mir-137	25	chr3	118425075	118425859	556	+	10.3419
TCCATCTTC	mmu-mir-138-1	-10	chr9	122416550	122422625	5493	-	9.92909
GCCATTTGT	mmu-mir-138-1	-10	chr9	122416550	122422625	4620	-	9.87983
GCCATTTGT	mmu-mir-138-1	-10	chr9	122416550	122422625	2169	+	9.87983
GCCATCTAG	mmu-mir-138-1	-10	chr9	122416550	122422625	3622	+	9.51535
GCCATCTTT	mmu-mir-141	20	chr6	124683151	124685075	1529	+	12.7433
GCCATCTTT	mmu-mir-141	20	chr6	124683151	124685075	383	+	12.7433
GCCATTTTG	mmu-mir-141	20	chr6	124683151	124685075	232	+	12.7008

TCCATCTTC	mmu-mir-141	20	chr6	124683151	124685075	441	-	9.92909
GCCATTGTG	mmu-mir-141	20	chr6	124683151	124685075	629	+	9.64448
GCCATGTTG	mmu-mir-142	0	chr11	87571775	87574325	1103	+	12.6637
GCCATGTTG	mmu-mir-142	0	chr11	87571775	87574325	709	-	12.6637
GCCATTTCT	mmu-mir-142	0	chr11	87571775	87574325	719	+	9.85738
TCCATCTTT	mmu-mir-143	20	chr18	61773267	61775675	1582	-	10.7594
TCCATTTTT	mmu-mir-143	20	chr18	61773267	61775675	2281	+	10.2548
TCCATCTTT	mmu-mir-145	20	chr18	61773267	61775675	1582	-	10.7594
TCCATTTTT	mmu-mir-145	20	chr18	61773267	61775675	2281	+	10.2548
ACCATCTTG	mmu-mir-146a	0	chr11	43227200	43230425	2505	-	10.3187
GCCATTTTT	mmu-mir-146b	0	chr19	46390100	46393375	1903	+	12.2387
GCCATTTTT	mmu-mir-146b	0	chr19	46390100	46393375	1996	+	12.2387
GCCATGTTT	mmu-mir-146b	0	chr19	46390100	46393375	84	-	12.2016
GCCATCTCG	mmu-mir-146b	0	chr19	46390100	46393375	1085	-	10.824
GCCATGTGG	mmu-mir-146b	0	chr19	46390100	46393375	1242	+	10.3048
TCCATCTTG	mmu-mir-148a	15	chr6	51198300	51201975	2184	+	11.2215
GCCATCTAG	mmu-mir-148a	15	chr6	51198300	51201975	3191	-	9.51535
TCCATCTTG	mmu-mir-148b	25	chr15	103100832	103101032	121	-	11.2215
GCTATCTTT	mmu-mir-154	0	chr12	110043400	110045125	868	+	9.56401
TCCATTTTC	mmu-mir-154	0	chr12	110043400	110045125	9	-	9.42452
ACCATGTTG	mmu-mir-155	0	chr16	84584475	84588475	155	-	9.77703
GCCATTTTC	mmu-mir-15a	15	chr14	60602792	60602992	96	-	11.4084
GCCATCTTC	mmu-mir-15b	25	chr3	69092800	69093000	12	-	11.913
GCCATTTTC	mmu-mir-15b	25	chr3	69092800	69093000	115	+	11.4084
GCCATTTTC	mmu-mir-16-1	15	chr14	60602792	60602992	96	-	11.4084
GCCATCTTC	mmu-mir-16-2	25	chr3	69092800	69093000	12	-	11.913
GCCATTTTC	mmu-mir-16-2	25	chr3	69092800	69093000	115	+	11.4084
GCCATCTTT	mmu-mir-17	10	chr14	113921300	113927025	5562	-	12.7433
GCCATGTTC	mmu-mir-17	10	chr14	113921300	113927025	2445	+	11.3713
GCCATTGTG	mmu-mir-17	10	chr14	113921300	113927025	1951	-	9.64448
GCCATCTTG	mmu-mir-181c	10	chr8	87069525	87071900	1042	+	13.2054
GCCATGTGT	mmu-mir-181c	10	chr8	87069525	87071900	1462	+	9.84271
GCCATCTTG	mmu-mir-181d	10	chr8	87069525	87071900	1042	+	13.2054
GCCATGTGT	mmu-mir-181d	10	chr8	87069525	87071900	1462	+	9.84271
GCCATCTGG	mmu-mir-182	10	chr6	30114875	30130825	9981	+	10.8465
GCCATCTCG	mmu-mir-182	10	chr6	30114875	30130825	5	-	10.824

GCCATCTGT	mmu-mir-182	10	chr6	30114875	30130825	10548	-	10.3844
GCCATCTGT	mmu-mir-182	10	chr6	30114875	30130825	4364	-	10.3844
GCCATCTGT	mmu-mir-182	10	chr6	30114875	30130825	782	+	10.3844
TCCATTTTT	mmu-mir-182	10	chr6	30114875	30130825	12153	-	10.2548
ACCATCTTT	mmu-mir-182	10	chr6	30114875	30130825	1880	-	9.85665
GCCATCTCC	mmu-mir-182	10	chr6	30114875	30130825	654	+	9.53163
TCCATTTTC	mmu-mir-182	10	chr6	30114875	30130825	14435	+	9.42452
TCCATTTTC	mmu-mir-182	10	chr6	30114875	30130825	13039	-	9.42452
GCCATCTGG	mmu-mir-183	10	chr6	30114875	30130825	9981	+	10.8465
GCCATCTCG	mmu-mir-183	10	chr6	30114875	30130825	5	-	10.824
GCCATCTGT	mmu-mir-183	10	chr6	30114875	30130825	4364	-	10.3844
GCCATCTGT	mmu-mir-183	10	chr6	30114875	30130825	782	+	10.3844
GCCATCTGT	mmu-mir-183	10	chr6	30114875	30130825	10548	-	10.3844
TCCATTTTT	mmu-mir-183	10	chr6	30114875	30130825	12153	-	10.2548
ACCATCTTT	mmu-mir-183	10	chr6	30114875	30130825	1880	-	9.85665
GCCATCTCC	mmu-mir-183	10	chr6	30114875	30130825	654	+	9.53163
TCCATTTTC	mmu-mir-183	10	chr6	30114875	30130825	14435	+	9.42452
TCCATTTTC	mmu-mir-183	10	chr6	30114875	30130825	13039	-	9.42452
GCCATATTC	mmu-mir-184	0	chr9	89676975	89678850	150	+	9.94887
GCCATGTGT	mmu-mir-184	0	chr9	89676975	89678850	285	+	9.84271
GCCATCTTC	mmu-mir-185	24	chr16	18261507	18261707	29	-	11.913
GCCATCTCT	mmu-mir-187	2	chr18	24608400	24611775	119	-	10.3619
GCCATCTTT	mmu-mir-18a	10	chr14	113921300	113927025	5562	-	12.7433
GCCATGTTC	mmu-mir-18a	10	chr14	113921300	113927025	2445	+	11.3713
GCCATTGTG	mmu-mir-18a	10	chr14	113921300	113927025	1951	-	9.64448
GCCATCTGT	mmu-mir-18b	0	chrX	48985925	48991150	1838	-	10.3844
GCCATCTGT	mmu-mir-18b	0	chrX	48985925	48991150	936	+	10.3844
GCCATCTCG	mmu-mir-190b	-15	chr3	90134875	90141050	5505	-	10.824
GCCATTTTA	mmu-mir-190b	-15	chr3	90134875	90141050	4532	-	10.5649
GCCATTTTA	mmu-mir-191	20	chr9	108424326	108426775	1281	-	10.5649
GCCATCTGC	mmu-mir-191	20	chr9	108424326	108426775	1121	-	9.55408
GCCATCTTG	mmu-mir-192	-10	chr19	6247500	6249425	139	-	13.2054
GCCATCTCT	mmu-mir-192	-10	chr19	6247500	6249425	1871	-	10.3619
ACCATCTTG	mmu-mir-193	10	chr11	79526175	79531800	3101	+	10.3187
ACCATTTTG	mmu-mir-193	10	chr11	79526175	79531800	3117	+	9.81414
TCCATCTTT	mmu-mir-193b	15	chr16	13359750	13363900	2946	-	10.7594

GCTATCTTT	mmu-mir-193b	15	chr16	13359750	13363900	3658	-	9.56401
GCCATCTCT	mmu-mir-194-1	0	chr1	186908300	186912075	89	-	10.3619
GCCATCTCT	mmu-mir-194-1	0	chr1	186908300	186912075	469	+	10.3619
GCCATCTCT	mmu-mir-194-1	0	chr1	186908300	186912075	3580	+	10.3619
GCCATGTGG	mmu-mir-194-1	0	chr1	186908300	186912075	2174	+	10.3048
TCCATTTTT	mmu-mir-194-1	0	chr1	186908300	186912075	622	-	10.2548
GCCATGGTG	mmu-mir-194-1	0	chr1	186908300	186912075	3034	+	9.60737
GCCATGGTG	mmu-mir-194-1	0	chr1	186908300	186912075	2762	+	9.60737
GCCATCTTG	mmu-mir-194-2	-10	chr19	6247500	6249425	139	-	13.2054
GCCATCTCT	mmu-mir-194-2	-10	chr19	6247500	6249425	1871	-	10.3619
ACCATGTTG	mmu-mir-196a-2	10	chr15	102799650	102802750	2933	-	9.77703
GCCATTTTT	mmu-mir-196b	10	chr6	52159743	52171275	5875	+	12.2387
GCCATCTCG	mmu-mir-196b	10	chr6	52159743	52171275	10380	+	10.824
GCCATGTCT	mmu-mir-196b	10	chr6	52159743	52171275	5847	-	9.82026
GCCATCTCC	mmu-mir-196b	10	chr6	52159743	52171275	2221	-	9.53163
GCCATATTT	mmu-mir-199a-1	10	chr9	21244900	21248875	1288	+	10.7792
GCCATCTCT	mmu-mir-199a-1	10	chr9	21244900	21248875	2406	-	10.3619
TCCATTTTT	mmu-mir-199a-2	10	chr1	164052250	164061375	6463	+	10.2548
ACCATGTTG	mmu-mir-199a-2	10	chr1	164052250	164061375	8574	+	9.77703
GCCATTATG	mmu-mir-199a-2	10	chr1	164052250	164061375	6174	+	9.69473
GCTATCTTT	mmu-mir-199a-2	10	chr1	164052250	164061375	8724	-	9.56401
GCTATGTTG	mmu-mir-199a-2	10	chr1	164052250	164061375	7940	-	9.4844
GCCATCTGG	mmu-mir-199b	15	chr2	32138775	32141975	2910	-	10.8465
GCCATCTCT	mmu-mir-199b	9	chr2	32136375	32138625	180	-	10.3619
GCCATGTGG	mmu-mir-199b	15	chr2	32138775	32141975	418	-	10.3048
GCCATGATG	mmu-mir-199b	15	chr2	32138775	32141975	3020	+	9.65762
GCCATTGTG	mmu-mir-199b	9	chr2	32136375	32138625	929	+	9.64448
GCCATTGTG	mmu-mir-199b	15	chr2	32138775	32141975	880	+	9.64448
GCCATCTTT	mmu-mir-19a	10	chr14	113921300	113927025	5562	-	12.7433
GCCATGTTC	mmu-mir-19a	10	chr14	113921300	113927025	2445	+	11.3713
GCCATTGTG	mmu-mir-19a	10	chr14	113921300	113927025	1951	-	9.64448
GCCATCTGT	mmu-mir-19b	0	chrX	48985925	48991150	936	+	10.3844
GCCATCTGT	mmu-mir-19b	0	chrX	48985925	48991150	1838	-	10.3844
GCCATCTTT	mmu-mir-200c	20	chr6	124683151	124685075	1529	+	12.7433
GCCATCTTT	mmu-mir-200c	20	chr6	124683151	124685075	383	+	12.7433
GCCATTTTG	mmu-mir-200c	20	chr6	124683151	124685075	232	+	12.7008

TCCATCTTC	mmu-mir-200c	20	chr6	124683151	124685075	441	-	9.92909
GCCATTGTG	mmu-mir-200c	20	chr6	124683151	124685075	629	+	9.64448
TCCATCTTG	mmu-mir-202	0	chr7	139821975	139823300	124	+	11.2215
GCCATTATG	mmu-mir-202	0	chr7	139821975	139823300	138	+	9.69473
GCTATGTTG	mmu-mir-203	15	chr12	112577075	112579650	2391	-	9.4844
GCCATCTTT	mmu-mir-20a	10	chr14	113921300	113927025	5562	-	12.7433
GCCATGTTC	mmu-mir-20a	10	chr14	113921300	113927025	2445	+	11.3713
GCCATTGTG	mmu-mir-20a	10	chr14	113921300	113927025	1951	-	9.64448
GCCATCTGT	mmu-mir-20b	0	chrX	48985925	48991150	936	+	10.3844
GCCATCTGT	mmu-mir-20b	0	chrX	48985925	48991150	1838	-	10.3844
TCCATTTTT	mmu-mir-214	10	chr1	164052250	164061375	6463	+	10.2548
ACCATGTTG	mmu-mir-214	10	chr1	164052250	164061375	8574	+	9.77703
GCCATTATG	mmu-mir-214	10	chr1	164052250	164061375	6174	+	9.69473
GCTATCTTT	mmu-mir-214	10	chr1	164052250	164061375	8724	-	9.56401
GCTATGTTG	mmu-mir-214	10	chr1	164052250	164061375	7940	-	9.4844
GCCATCTCT	mmu-mir-215	0	chr1	186908300	186912075	3580	+	10.3619
GCCATCTCT	mmu-mir-215	0	chr1	186908300	186912075	469	+	10.3619
GCCATCTCT	mmu-mir-215	0	chr1	186908300	186912075	89	-	10.3619
GCCATGTGG	mmu-mir-215	0	chr1	186908300	186912075	2174	+	10.3048
TCCATTTTT	mmu-mir-215	0	chr1	186908300	186912075	622	-	10.2548
GCCATGGTG	mmu-mir-215	0	chr1	186908300	186912075	3034	+	9.60737
GCCATGGTG	mmu-mir-215	0	chr1	186908300	186912075	2762	+	9.60737
GCCATGGTG	mmu-mir-218-2	23	chr11	34964780	34964980	153	-	9.60737
TCCATCTTG	mmu-mir-220	0	chr6	136427625	136429100	149	-	11.2215
GCCATCTTA	mmu-mir-221	-10	chrX	18319475	18326525	148	+	11.0695
GCCATTTTA	mmu-mir-221	-10	chrX	18319475	18326525	1	-	10.5649
GCCATCTCT	mmu-mir-221	-10	chrX	18319475	18326525	5459	+	10.3619
TCCATTTTT	mmu-mir-221	-10	chrX	18319475	18326525	2026	-	10.2548
TCCATTTTT	mmu-mir-221	-10	chrX	18319475	18326525	6557	-	10.2548
GCTATTTTG	mmu-mir-221	-10	chrX	18319475	18326525	2423	-	9.52151
GCCATCTTA	mmu-mir-222	-10	chrX	18319475	18326525	148	+	11.0695
GCCATTTTA	mmu-mir-222	-10	chrX	18319475	18326525	1	-	10.5649
GCCATCTCT	mmu-mir-222	-10	chrX	18319475	18326525	5459	+	10.3619
TCCATTTTT	mmu-mir-222	-10	chrX	18319475	18326525	6557	-	10.2548
TCCATTTTT	mmu-mir-222	-10	chrX	18319475	18326525	2026	-	10.2548
GCTATTTTG	mmu-mir-222	-10	chrX	18319475	18326525	2423	-	9.52151

GCCATCTTG	mmu-mir-223	-10	chrX	92297875	92303675	4640	+	13.2054
ACCATCTTG	mmu-mir-223	-10	chrX	92297875	92303675	4873	+	10.3187
GCCATCATG	mmu-mir-223	-10	chrX	92297875	92303675	2716	+	10.1993
GCTATTTTG	mmu-mir-223	-10	chrX	92297875	92303675	4943	-	9.52151
TCCATTTTG	mmu-mir-23a	0	chr8	87086300	87095525	2966	-	10.7169
GCCATCTCT	mmu-mir-23a	0	chr8	87086300	87095525	121	+	10.3619
GCCATCTCT	mmu-mir-23a	0	chr8	87086300	87095525	8696	+	10.3619
GCCATTTGG	mmu-mir-23a	0	chr8	87086300	87095525	7990	-	10.3419
ACCATCTTT	mmu-mir-23a	0	chr8	87086300	87095525	3940	-	9.85665
GCCATGTGT	mmu-mir-23a	0	chr8	87086300	87095525	6994	-	9.84271
GCTATTTTG	mmu-mir-23a	0	chr8	87086300	87095525	6912	-	9.52151
TCCATTTTG	mmu-mir-24-2	0	chr8	87086300	87095525	2966	-	10.7169
GCCATCTCT	mmu-mir-24-2	0	chr8	87086300	87095525	8696	+	10.3619
GCCATCTCT	mmu-mir-24-2	0	chr8	87086300	87095525	121	+	10.3619
GCCATTTGG	mmu-mir-24-2	0	chr8	87086300	87095525	7990	-	10.3419
ACCATCTTT	mmu-mir-24-2	0	chr8	87086300	87095525	3940	-	9.85665
GCCATGTGT	mmu-mir-24-2	0	chr8	87086300	87095525	6994	-	9.84271
GCTATTTTG	mmu-mir-24-2	0	chr8	87086300	87095525	6912	-	9.52151
TCCATGTTT	mmu-mir-26b	30	chr1	74324705	74324905	168	+	10.2177
TCCATTTTG	mmu-mir-27a	0	chr8	87086300	87095525	2966	-	10.7169
GCCATCTCT	mmu-mir-27a	0	chr8	87086300	87095525	121	+	10.3619
GCCATCTCT	mmu-mir-27a	0	chr8	87086300	87095525	8696	+	10.3619
GCCATTTGG	mmu-mir-27a	0	chr8	87086300	87095525	7990	-	10.3419
ACCATCTTT	mmu-mir-27a	0	chr8	87086300	87095525	3940	-	9.85665
GCCATGTGT	mmu-mir-27a	0	chr8	87086300	87095525	6994	-	9.84271
GCTATTTTG	mmu-mir-27a	0	chr8	87086300	87095525	6912	-	9.52151
GCCATTTTG	mmu-mir-296	-15	chr2	173925825	173933325	3725	+	12.7008
GCCATCTTC	mmu-mir-296	-15	chr2	173925825	173933325	3734	+	11.913
GCCATTTTC	mmu-mir-296	-15	chr2	173925825	173933325	5086	+	11.4084
GCCATGATG	mmu-mir-296	-15	chr2	173925825	173933325	473	+	9.65762
GCTATGTTG	mmu-mir-296	-15	chr2	173925825	173933325	6379	+	9.4844
GCCATCTTT	mmu-mir-297a-7	-10	chr10	42708700	42711800	2871	-	12.7433
TCCATTTTG	mmu-mir-297a-7	-10	chr10	42708700	42711800	2885	-	10.7169
TCCATTTTG	mmu-mir-297a-7	-10	chr10	42708700	42711800	2520	+	10.7169
GCCATCGTG	mmu-mir-297a-7	-10	chr10	42708700	42711800	610	-	10.1491
GCCATTTTG	mmu-mir-298	-15	chr2	173925825	173933325	3725	+	12.7008

GCCATCTTC	mmu-mir-298	-15	chr2	173925825	173933325	3734	+	11.913
GCCATTTTC	mmu-mir-298	-15	chr2	173925825	173933325	5086	+	11.4084
GCCATGATG	mmu-mir-298	-15	chr2	173925825	173933325	473	+	9.65762
GCTATGTTG	mmu-mir-298	-15	chr2	173925825	173933325	6379	+	9.4844
GCTATCTTT	mmu-mir-299	0	chr12	110043400	110045125	868	+	9.56401
TCCATTTTC	mmu-mir-299	0	chr12	110043400	110045125	9	-	9.42452
GCCATTTTG	mmu-mir-29b-2	-10	chr1	196676100	196679875	3604	+	12.7008
TCCATTTTT	mmu-mir-29b-2	-10	chr1	196676100	196679875	3008	+	10.2548
GCCATTTTG	mmu-mir-29c	-10	chr1	196676100	196679875	3604	+	12.7008
TCCATTTTT	mmu-mir-29c	-10	chr1	196676100	196679875	3008	+	10.2548
GCTATCTTT	mmu-mir-300	0	chr12	110043400	110045125	868	+	9.56401
TCCATTTTC	mmu-mir-300	0	chr12	110043400	110045125	9	-	9.42452
TCCATCTTT	mmu-mir-301b	30	chr16	17038569	17038769	114	+	10.7594
TCCATGTTT	mmu-mir-302a	10	chr3	127537000	127537941	694	+	10.2177
TCCATGTTT	mmu-mir-302a	10	chr3	127537000	127537941	568	+	10.2177
TCCATGTTT	mmu-mir-302a	10	chr3	127537000	127537941	434	+	10.2177
TCCATGTTT	mmu-mir-302a	10	chr3	127537000	127537941	304	+	10.2177
TCCATGTTT	mmu-mir-302b	10	chr3	127537000	127537941	694	+	10.2177
TCCATGTTT	mmu-mir-302b	10	chr3	127537000	127537941	568	+	10.2177
TCCATGTTT	mmu-mir-302b	10	chr3	127537000	127537941	434	+	10.2177
TCCATGTTT	mmu-mir-302b	10	chr3	127537000	127537941	304	+	10.2177
TCCATGTTT	mmu-mir-302c	10	chr3	127537000	127537941	434	+	10.2177
TCCATGTTT	mmu-mir-302c	10	chr3	127537000	127537941	568	+	10.2177
TCCATGTTT	mmu-mir-302c	10	chr3	127537000	127537941	694	+	10.2177
TCCATGTTT	mmu-mir-302c	10	chr3	127537000	127537941	304	+	10.2177
TCCATGTTT	mmu-mir-302d	10	chr3	127537000	127537941	304	+	10.2177
TCCATGTTT	mmu-mir-302d	10	chr3	127537000	127537941	434	+	10.2177
TCCATGTTT	mmu-mir-302d	10	chr3	127537000	127537941	568	+	10.2177
TCCATGTTT	mmu-mir-302d	10	chr3	127537000	127537941	694	+	10.2177
GCCATCTTG	mmu-mir-30b	-8	chr15	68190175	68194550	913	-	13.2054
GCCATATTC	mmu-mir-30b	-8	chr15	68190175	68194550	99	+	9.94887
TCCATTTTC	mmu-mir-30b	-8	chr15	68190175	68194550	559	-	9.42452
GCCATCTTG	mmu-mir-30c-1	14	chr4	120323146	120323346	163	-	13.2054
GCCATCTTG	mmu-mir-30e	14	chr4	120323146	120323346	163	-	13.2054
GCCATCTTT	mmu-mir-31	0	chr4	88399300	88401975	1053	-	12.7433
GCCATCTTA	mmu-mir-320	2	chr14	69176250	69180100	2271	+	11.0695

GCCATTTTG	mmu-mir-322	0	chrX	49298875	49304275	1728	-	12.7008
GCCATTTTC	mmu-mir-322	0	chrX	49298875	49304275	2423	-	11.4084
TCCATTTTG	mmu-mir-322	0	chrX	49298875	49304275	779	-	10.7169
GCTATCTTT	mmu-mir-323	0	chr12	110043400	110045125	868	+	9.56401
TCCATTTTC	mmu-mir-323	0	chr12	110043400	110045125	9	-	9.42452
TCCATTTTC	mmu-mir-326	10	chr7	99425150	99427125	378	+	9.42452
GCCATTTGT	mmu-mir-327	-10	chr14	43909450	43912875	498	+	9.87983
GCCATTATG	mmu-mir-327	-10	chr14	43909450	43912875	2189	+	9.69473
GCCATCTTC	mmu-mir-328	-15	chr8	108213550	108217600	1568	+	11.913
TCCATTTTG	mmu-mir-328	-15	chr8	108213550	108217600	1169	-	10.7169
TCCATGTTG	mmu-mir-328	-15	chr8	108213550	108217600	4013	-	10.6798
GCCATCTGT	mmu-mir-328	-15	chr8	108213550	108217600	3896	-	10.3844
TCCATCTTC	mmu-mir-328	-15	chr8	108213550	108217600	2461	-	9.92909
GCCATGGTG	mmu-mir-328	-15	chr8	108213550	108217600	2043	-	9.60737
GCTATCTTT	mmu-mir-329	0	chr12	110043400	110045125	868	+	9.56401
TCCATTTTC	mmu-mir-329	0	chr12	110043400	110045125	9	-	9.42452
GCCATGTTT	mmu-mir-33	17	chr15	81974358	81974558	162	-	12.2016
GCCATCTTT	mmu-mir-331	0	chr10	93443775	93445175	924	-	12.7433
GCCATGGTG	mmu-mir-331	0	chr10	93443775	93445175	1074	-	9.60737
GCCATCTCC	mmu-mir-331	0	chr10	93443775	93445175	1249	-	9.53163
GCCATCTTT	mmu-mir-337	-10	chr12	109987400	109991350	1969	+	12.7433
GCCATCTTT	mmu-mir-337	-10	chr12	109987400	109991350	3284	-	12.7433
GCCATTTTC	mmu-mir-337	-10	chr12	109987400	109991350	3240	-	11.4084
GCCATCTCT	mmu-mir-337	-10	chr12	109987400	109991350	2771	-	10.3619
GCCATCATG	mmu-mir-337	-10	chr12	109987400	109991350	2388	-	10.1993
GCCATGTCT	mmu-mir-337	-10	chr12	109987400	109991350	1896	-	9.82026
GCCATCTGC	mmu-mir-337	-10	chr12	109987400	109991350	1034	+	9.55408
GCCATGTCG	mmu-mir-338	20	chr11	119862940	119863140	67	-	10.2823
GCCATCTTG	mmu-mir-339	10	chr5	139624300	139627075	2471	+	13.2054
TCCATCTTC	mmu-mir-339	22	chr5	139714076	139714276	146	+	9.92909
GCCATTTCT	mmu-mir-339	10	chr5	139624300	139627075	480	+	9.85738
GCTATCTTT	mmu-mir-341	0	chr12	110043400	110045125	868	+	9.56401
TCCATTTTC	mmu-mir-341	0	chr12	110043400	110045125	9	-	9.42452
GCCATTTGT	mmu-mir-34a	0	chr4	148890450	148895975	4351	-	9.87983
GCCATCTTC	mmu-mir-34b	20	chr9	50855701	50858400	1478	+	11.913
GCCATCTTC	mmu-mir-34c	20	chr9	50855701	50858400	1478	+	11.913

GCCATGTTG	mmu-mir-350	0	chr1	178636600	178640175	2757	-	12.6637
GCCATCTGG	mmu-mir-350	0	chr1	178636600	178640175	1457	-	10.8465
TCCATTTTT	mmu-mir-350	0	chr1	178636600	178640175	3019	-	10.2548
TCCATTTTC	mmu-mir-350	0	chr1	178636600	178640175	800	+	9.42452
GCCATCTTC	mmu-mir-351	10	chrX	49296350	49298075	534	+	11.913
GCCATTTTC	mmu-mir-351	10	chrX	49296350	49298075	1665	+	11.4084
GCCATATTG	mmu-mir-351	10	chrX	49296350	49298075	93	-	11.2413
GCCATCTGG	mmu-mir-351	10	chrX	49296350	49298075	852	-	10.8465
GCCATTTGT	mmu-mir-351	10	chrX	49296350	49298075	1046	+	9.87983
GCCATGTCT	mmu-mir-351	10	chrX	49296350	49298075	261	+	9.82026
GCCATCTTG	mmu-mir-361	20	chrX	109302359	109302559	69	+	13.2054
GCCATCTGT	mmu-mir-363	0	chrX	48985925	48991150	1838	-	10.3844
GCCATCTGT	mmu-mir-363	0	chrX	48985925	48991150	936	+	10.3844
TCCATCTTT	mmu-mir-365-1	6	chr16	13359750	13363900	2946	-	10.7594
GCTATCTTT	mmu-mir-365-1	6	chr16	13359750	13363900	3658	-	9.56401
ACCATCTTG	mmu-mir-365-2	0	chr11	79526175	79531800	3101	+	10.3187
ACCATTTTG	mmu-mir-365-2	0	chr11	79526175	79531800	3117	+	9.81414
TCCATGTTT	mmu-mir-367	10	chr3	127537000	127537941	568	+	10.2177
TCCATGTTT	mmu-mir-367	10	chr3	127537000	127537941	694	+	10.2177
TCCATGTTT	mmu-mir-367	10	chr3	127537000	127537941	434	+	10.2177
TCCATGTTT	mmu-mir-367	10	chr3	127537000	127537941	304	+	10.2177
GCTATCTTT	mmu-mir-368	0	chr12	110043400	110045125	868	+	9.56401
TCCATTTTC	mmu-mir-368	0	chr12	110043400	110045125	9	-	9.42452
GCTATCTTT	mmu-mir-369	0	chr12	110043400	110045125	868	+	9.56401
TCCATTTTC	mmu-mir-369	0	chr12	110043400	110045125	9	-	9.42452
GCTATCTTT	mmu-mir-370	0	chr12	110043400	110045125	868	+	9.56401
TCCATTTTC	mmu-mir-370	0	chr12	110043400	110045125	9	-	9.42452
TCCATCTTC	mmu-mir-374	0	chrX	99817575	99820975	2541	+	9.92909
TCCATCTTC	mmu-mir-374	0	chrX	99817575	99820975	2396	-	9.92909
ACCATTTTG	mmu-mir-374	0	chrX	99817575	99820975	2253	-	9.81414
GCTATCTTT	mmu-mir-376a	0	chr12	110043400	110045125	868	+	9.56401
TCCATTTTC	mmu-mir-376a	0	chr12	110043400	110045125	9	-	9.42452
GCTATCTTT	mmu-mir-376b	0	chr12	110043400	110045125	868	+	9.56401
TCCATTTTC	mmu-mir-376b	0	chr12	110043400	110045125	9	-	9.42452
GCTATCTTT	mmu-mir-377	0	chr12	110043400	110045125	868	+	9.56401
TCCATTTTC	mmu-mir-377	0	chr12	110043400	110045125	9	-	9.42452

GCCATCTTC	mmu-mir-378	35	chr18	61525700	61525900	31	+	11.913
GCTATCTTT	mmu-mir-379	0	chr12	110043400	110045125	868	+	9.56401
TCCATTTTC	mmu-mir-379	0	chr12	110043400	110045125	9	-	9.42452
GCTATCTTT	mmu-mir-380	0	chr12	110043400	110045125	868	+	9.56401
TCCATTTTC	mmu-mir-380	0	chr12	110043400	110045125	9	-	9.42452
GCTATCTTT	mmu-mir-381	0	chr12	110043400	110045125	868	+	9.56401
TCCATTTTC	mmu-mir-381	0	chr12	110043400	110045125	9	-	9.42452
GCTATCTTT	mmu-mir-382	0	chr12	110043400	110045125	868	+	9.56401
TCCATTTTC	mmu-mir-382	0	chr12	110043400	110045125	9	-	9.42452
GCCATCTTC	mmu-mir-384	-10	chrX	101592850	101595950	1382	+	11.913
GCCATTTTC	mmu-mir-384	-10	chrX	101592850	101595950	1521	+	11.4084
TCCATCTTG	mmu-mir-384	-10	chrX	101592850	101595950	552	+	11.2215
GCCATTTTA	mmu-mir-384	-10	chrX	101592850	101595950	311	-	10.5649
GCCATTTGT	mmu-mir-384	-10	chrX	101592850	101595950	991	+	9.87983
ACCATCTTT	mmu-mir-384	-10	chrX	101592850	101595950	2224	+	9.85665
GCTATCTTT	mmu-mir-409	0	chr12	110043400	110045125	868	+	9.56401
TCCATTTTC	mmu-mir-409	0	chr12	110043400	110045125	9	-	9.42452
GCTATCTTT	mmu-mir-410	0	chr12	110043400	110045125	868	+	9.56401
TCCATTTTC	mmu-mir-410	0	chr12	110043400	110045125	9	-	9.42452
GCTATCTTT	mmu-mir-411	0	chr12	110043400	110045125	868	+	9.56401
TCCATTTTC	mmu-mir-411	0	chr12	110043400	110045125	9	-	9.42452
GCTATCTTT	mmu-mir-412	0	chr12	110043400	110045125	868	+	9.56401
TCCATTTTC	mmu-mir-412	0	chr12	110043400	110045125	9	-	9.42452
GCCATCTTT	mmu-mir-421	0	chrX	99822432	99826775	1073	-	12.7433
GCCATTTTA	mmu-mir-421	0	chrX	99822432	99826775	1299	+	10.5649
GCCATTTTA	mmu-mir-421	0	chrX	99822432	99826775	2579	-	10.5649
GCCATCTTG	mmu-mir-423	35	chr11	76894530	76894730	58	+	13.2054
GCCATTTTA	mmu-mir-425	20	chr9	108424326	108426775	1281	-	10.5649
GCCATCTGC	mmu-mir-425	20	chr9	108424326	108426775	1121	-	9.55408
GCCATCTTT	mmu-mir-431	-10	chr12	109987400	109991350	1969	+	12.7433
GCCATCTTT	mmu-mir-431	-10	chr12	109987400	109991350	3284	-	12.7433
GCCATTTTC	mmu-mir-431	-10	chr12	109987400	109991350	3240	-	11.4084
GCCATCTCT	mmu-mir-431	-10	chr12	109987400	109991350	2771	-	10.3619
GCCATCATG	mmu-mir-431	-10	chr12	109987400	109991350	2388	-	10.1993
GCCATGTCT	mmu-mir-431	-10	chr12	109987400	109991350	1896	-	9.82026
GCCATCTGC	mmu-mir-431	-10	chr12	109987400	109991350	1034	+	9.55408

GCCATCTTT	mmu-mir-433	-10	chr12	109987400	109991350	3284	-	12.7433
GCCATCTTT	mmu-mir-433	-10	chr12	109987400	109991350	1969	+	12.7433
GCCATTTTC	mmu-mir-433	-10	chr12	109987400	109991350	3240	-	11.4084
GCCATCTCT	mmu-mir-433	-10	chr12	109987400	109991350	2771	-	10.3619
GCCATCATG	mmu-mir-433	-10	chr12	109987400	109991350	2388	-	10.1993
GCCATGTCT	mmu-mir-433	-10	chr12	109987400	109991350	1896	-	9.82026
GCCATCTGC	mmu-mir-433	-10	chr12	109987400	109991350	1034	+	9.55408
GCCATCTTT	mmu-mir-434	-10	chr12	109987400	109991350	3284	-	12.7433
GCCATCTTT	mmu-mir-434	-10	chr12	109987400	109991350	1969	+	12.7433
GCCATTTTC	mmu-mir-434	-10	chr12	109987400	109991350	3240	-	11.4084
GCCATCTCT	mmu-mir-434	-10	chr12	109987400	109991350	2771	-	10.3619
GCCATCATG	mmu-mir-434	-10	chr12	109987400	109991350	2388	-	10.1993
GCCATGTCT	mmu-mir-434	-10	chr12	109987400	109991350	1896	-	9.82026
GCCATCTGC	mmu-mir-434	-10	chr12	109987400	109991350	1034	+	9.55408
GCCATCTGG	mmu-mir-449a	15	chr13	114154825	114157525	2161	+	10.8465
GCCATCTCT	mmu-mir-449a	15	chr13	114154825	114157525	2485	+	10.3619
ACCATCTTT	mmu-mir-449a	15	chr13	114154825	114157525	33	-	9.85665
GCCATCTGG	mmu-mir-449b	15	chr13	114154825	114157525	2161	+	10.8465
GCCATCTCT	mmu-mir-449b	15	chr13	114154825	114157525	2485	+	10.3619
ACCATCTTT	mmu-mir-449b	15	chr13	114154825	114157525	33	-	9.85665
GCCATCTTC	mmu-mir-450a-1	0	chrX	49296350	49298075	534	+	11.913
GCCATTTTC	mmu-mir-450a-1	0	chrX	49296350	49298075	1665	+	11.4084
GCCATATTG	mmu-mir-450a-1	0	chrX	49296350	49298075	93	-	11.2413
GCCATCTGG	mmu-mir-450a-1	0	chrX	49296350	49298075	852	-	10.8465
GCCATTTGT	mmu-mir-450a-1	0	chrX	49296350	49298075	1046	+	9.87983
GCCATGTCT	mmu-mir-450a-1	0	chrX	49296350	49298075	261	+	9.82026
GCCATCTTC	mmu-mir-450a-2	0	chrX	49296350	49298075	534	+	11.913
GCCATTTTC	mmu-mir-450a-2	0	chrX	49296350	49298075	1665	+	11.4084
GCCATATTG	mmu-mir-450a-2	0	chrX	49296350	49298075	93	-	11.2413
GCCATCTGG	mmu-mir-450a-2	0	chrX	49296350	49298075	852	-	10.8465
GCCATTTGT	mmu-mir-450a-2	0	chrX	49296350	49298075	1046	+	9.87983
GCCATGTCT	mmu-mir-450a-2	0	chrX	49296350	49298075	261	+	9.82026
GCCATCTTC	mmu-mir-450b	0	chrX	49296350	49298075	534	+	11.913
GCCATTTTC	mmu-mir-450b	0	chrX	49296350	49298075	1665	+	11.4084
GCCATATTG	mmu-mir-450b	0	chrX	49296350	49298075	93	-	11.2413
GCCATCTGG	mmu-mir-450b	0	chrX	49296350	49298075	852	-	10.8465

GCCATTTGT	mmu-mir-450b	0	chrX	49296350	49298075	1046	+	9.87983
GCCATGTCT	mmu-mir-450b	0	chrX	49296350	49298075	261	+	9.82026
GCTATCTTT	mmu-mir-453	0	chr12	110043400	110045125	868	+	9.56401
TCCATTTTC	mmu-mir-453	0	chr12	110043400	110045125	9	-	9.42452
GCCATGTCG	mmu-mir-484	2	chr16	14070875	14074575	2447	+	10.2823
TCCATTTTC	mmu-mir-484	2	chr16	14070875	14074575	20	+	9.42452
GCTATCTTT	mmu-mir-485	0	chr12	110043400	110045125	868	+	9.56401
TCCATTTTC	mmu-mir-485	0	chr12	110043400	110045125	9	-	9.42452
GCTATCTTT	mmu-mir-487b	0	chr12	110043400	110045125	868	+	9.56401
TCCATTTTC	mmu-mir-487b	0	chr12	110043400	110045125	9	-	9.42452
GCCATCTTT	mmu-mir-493	-10	chr12	109987400	109991350	3284	-	12.7433
GCCATCTTT	mmu-mir-493	-10	chr12	109987400	109991350	1969	+	12.7433
GCCATTTTC	mmu-mir-493	-10	chr12	109987400	109991350	3240	-	11.4084
GCCATCTCT	mmu-mir-493	-10	chr12	109987400	109991350	2771	-	10.3619
GCCATCATG	mmu-mir-493	-10	chr12	109987400	109991350	2388	-	10.1993
GCCATGTCT	mmu-mir-493	-10	chr12	109987400	109991350	1896	-	9.82026
GCCATCTGC	mmu-mir-493	-10	chr12	109987400	109991350	1034	+	9.55408
GCTATCTTT	mmu-mir-494	0	chr12	110043400	110045125	868	+	9.56401
TCCATTTTC	mmu-mir-494	0	chr12	110043400	110045125	9	-	9.42452
GCTATCTTT	mmu-mir-495	0	chr12	110043400	110045125	868	+	9.56401
TCCATTTTC	mmu-mir-495	0	chr12	110043400	110045125	9	-	9.42452
GCTATCTTT	mmu-mir-496	0	chr12	110043400	110045125	868	+	9.56401
TCCATTTTC	mmu-mir-496	0	chr12	110043400	110045125	9	-	9.42452
GCCATTTTG	mmu-mir-503	0	chrX	49298875	49304275	1728	-	12.7008
GCCATTTTC	mmu-mir-503	0	chrX	49298875	49304275	2423	-	11.4084
TCCATTTTG	mmu-mir-503	0	chrX	49298875	49304275	779	-	10.7169
GCTATCTTT	mmu-mir-539	0	chr12	110043400	110045125	868	+	9.56401
TCCATTTTC	mmu-mir-539	0	chr12	110043400	110045125	9	-	9.42452
GCCATCTTT	mmu-mir-540	-10	chr12	109987400	109991350	3284	-	12.7433
GCCATCTTT	mmu-mir-540	-10	chr12	109987400	109991350	1969	+	12.7433
GCCATTTTC	mmu-mir-540	-10	chr12	109987400	109991350	3240	-	11.4084
GCCATCTCT	mmu-mir-540	-10	chr12	109987400	109991350	2771	-	10.3619
GCCATCATG	mmu-mir-540	-10	chr12	109987400	109991350	2388	-	10.1993
GCCATGTCT	mmu-mir-540	-10	chr12	109987400	109991350	1896	-	9.82026
GCCATCTGC	mmu-mir-540	-10	chr12	109987400	109991350	1034	+	9.55408
GCTATCTTT	mmu-mir-541	0	chr12	110043400	110045125	868	+	9.56401

TCCATTTTC	mmu-mir-541	0	chr12	110043400	110045125	9	-	9.42452
GCCATCTTC	mmu-mir-542	10	chrX	49296350	49298075	534	+	11.913
GCCATTTTC	mmu-mir-542	10	chrX	49296350	49298075	1665	+	11.4084
GCCATATTG	mmu-mir-542	10	chrX	49296350	49298075	93	-	11.2413
GCCATCTGG	mmu-mir-542	10	chrX	49296350	49298075	852	-	10.8465
GCCATTTGT	mmu-mir-542	10	chrX	49296350	49298075	1046	+	9.87983
GCCATGTCT	mmu-mir-542	10	chrX	49296350	49298075	261	+	9.82026
GCTATCTTT	mmu-mir-543	0	chr12	110043400	110045125	868	+	9.56401
TCCATTTTC	mmu-mir-543	0	chr12	110043400	110045125	9	-	9.42452
GCTATCTTT	mmu-mir-544	0	chr12	110043400	110045125	868	+	9.56401
TCCATTTTC	mmu-mir-544	0	chr12	110043400	110045125	9	-	9.42452
GCCATCTTG	mmu-mir-598	0	chr14	62559175	62563900	1220	+	13.2054
GCCATCTTG	mmu-mir-598	0	chr14	62559175	62563900	1460	-	13.2054
GCCATTTTG	mmu-mir-598	0	chr14	62559175	62563900	837	+	12.7008
GCCATGTTT	mmu-mir-598	0	chr14	62559175	62563900	312	+	12.2016
GCTATCTTT	mmu-mir-654	0	chr12	110043400	110045125	868	+	9.56401
TCCATTTTC	mmu-mir-654	0	chr12	110043400	110045125	9	-	9.42452
GCCATCTTT	mmu-mir-665	-10	chr12	109987400	109991350	1969	+	12.7433
GCCATCTTT	mmu-mir-665	-10	chr12	109987400	109991350	3284	-	12.7433
GCCATTTTC	mmu-mir-665	-10	chr12	109987400	109991350	3240	-	11.4084
GCCATCTCT	mmu-mir-665	-10	chr12	109987400	109991350	2771	-	10.3619
GCCATCATG	mmu-mir-665	-10	chr12	109987400	109991350	2388	-	10.1993
GCCATGTCT	mmu-mir-665	-10	chr12	109987400	109991350	1896	-	9.82026
GCCATCTGC	mmu-mir-665	-10	chr12	109987400	109991350	1034	+	9.55408
GCTATCTTT	mmu-mir-666	0	chr12	110043400	110045125	868	+	9.56401
TCCATTTTC	mmu-mir-666	0	chr12	110043400	110045125	9	-	9.42452
GCCATCTTT	mmu-mir-673	-10	chr12	109987400	109991350	1969	+	12.7433
GCCATCTTT	mmu-mir-673	-10	chr12	109987400	109991350	3284	-	12.7433
GCCATTTTC	mmu-mir-673	-10	chr12	109987400	109991350	3240	-	11.4084
GCCATCTCT	mmu-mir-673	-10	chr12	109987400	109991350	2771	-	10.3619
GCCATCATG	mmu-mir-673	-10	chr12	109987400	109991350	2388	-	10.1993
GCCATGTCT	mmu-mir-673	-10	chr12	109987400	109991350	1896	-	9.82026
GCCATCTGC	mmu-mir-673	-10	chr12	109987400	109991350	1034	+	9.55408
GCTATCTTT	mmu-mir-758	0	chr12	110043400	110045125	868	+	9.56401
TCCATTTTC	mmu-mir-758	0	chr12	110043400	110045125	9	-	9.42452
GCCATTTTA	mmu-mir-760	22	chr3	122285704	122287675	1255	-	10.5649

ACCATTTTG	mmu-mir-760	22	chr3	122285704	122287675	937	-	9.81414
GCCATCTTT	mmu-mir-770	-10	chr12	109987400	109991350	3284	-	12.7433
GCCATCTTT	mmu-mir-770	-10	chr12	109987400	109991350	1969	+	12.7433
GCCATTTTC	mmu-mir-770	-10	chr12	109987400	109991350	3240	-	11.4084
GCCATCTCT	mmu-mir-770	-10	chr12	109987400	109991350	2771	-	10.3619
GCCATCATG	mmu-mir-770	-10	chr12	109987400	109991350	2388	-	10.1993
GCCATGTCT	mmu-mir-770	-10	chr12	109987400	109991350	1896	-	9.82026
GCCATCTGC	mmu-mir-770	-10	chr12	109987400	109991350	1034	+	9.55408
TCCATCTTT	mmu-mir-802	10	chr16	93257500	93258650	480	-	10.7594
GCCATGTTT	mmu-mir-873	0	chr4	37072975	37074125	161	-	12.2016
TCCATTTTT	mmu-mir-873	0	chr4	37072975	37074125	463	+	10.2548
GCCATCTCT	mmu-mir-874	0	chr13	58075200	58078100	2529	-	10.3619
TCCATTTTC	mmu-mir-875	0	chr15	35693875	35694875	373	-	9.42452
GCCATGTTT	mmu-mir-876	0	chr4	37072975	37074125	161	-	12.2016
TCCATTTTT	mmu-mir-876	0	chr4	37072975	37074125	463	+	10.2548
GCTATCTTT	mmu-mir-882	0	chr12	110043400	110045125	868	+	9.56401
TCCATTTTC	mmu-mir-882	0	chr12	110043400	110045125	9	-	9.42452
GCCATCTGT	mmu-mir-92a	0	chrX	48985925	48991150	1838	-	10.3844
GCCATCTGT	mmu-mir-92a	0	chrX	48985925	48991150	936	+	10.3844
GCCATGGTG	mmu-mir-92b	20	chr3	89313125	89315675	245	+	9.60737
GCCATCTTT	mmu-mir-9-3	10	chr7	79377250	79382900	505	-	12.7433
TCCATCTTG	mmu-mir-9-3	10	chr7	79377250	79382900	3213	+	11.2215
TCCATCTTT	mmu-mir-9-3	10	chr7	79377250	79382900	3327	+	10.7594
GCCATTTTA	mmu-mir-9-3	10	chr7	79377250	79382900	5330	+	10.5649
GCCATCTGG	mmu-mir-96	10	chr6	30114875	30130825	9981	+	10.8465
GCCATCTCG	mmu-mir-96	10	chr6	30114875	30130825	5	-	10.824
GCCATCTGT	mmu-mir-96	10	chr6	30114875	30130825	782	+	10.3844
GCCATCTGT	mmu-mir-96	10	chr6	30114875	30130825	4364	-	10.3844
GCCATCTGT	mmu-mir-96	10	chr6	30114875	30130825	10548	-	10.3844
TCCATTTTT	mmu-mir-96	10	chr6	30114875	30130825	12153	-	10.2548
ACCATCTTT	mmu-mir-96	10	chr6	30114875	30130825	1880	-	9.85665
GCCATCTCC	mmu-mir-96	10	chr6	30114875	30130825	654	+	9.53163
TCCATTTTC	mmu-mir-96	10	chr6	30114875	30130825	14435	+	9.42452
TCCATTTTC	mmu-mir-96	10	chr6	30114875	30130825	13039	-	9.42452
ACCATTTTG	mmu-mir-99b	20	chr17	17530726	17533550	557	-	9.81414

Suppl. Table S1: Predicted promoter regions for mouse miRNAs.
Position weight matrix,YY1_Q6_02, was used to scan for YY1 motif.

Motif Sequence	miRNA ID	Promoter Score	Promoter Chr	Promoter Start	Promoter End	Relative Motif Location	Strand	Motif Search Score
TTAGCCATTTT	mmu-let-7a-1	10	chr13	48551775	48553275	571	-	11.1306
ACATCCATCTT	mmu-let-7a-1	10	chr13	48551775	48553275	678	+	10.0343
ACATCCATATT	mmu-let-7b	0	chr15	85515125	85516200	783	-	9.92606
GTCCCCATTTT	mmu-let-7b	0	chr15	85515125	85516200	906	-	9.88382
TTCTCCATATT	mmu-let-7c-1	10	chr16	77477393	77477593	50	-	10.8664
ACATCCATATT	mmu-let-7c-2	0	chr15	85515125	85516200	783	-	9.92606
GTCCCCATTTT	mmu-let-7c-2	0	chr15	85515125	85516200	906	-	9.88382
TTAGCCATTTT	mmu-let-7d	0	chr13	48551775	48553275	571	-	11.1306
ACATCCATCTT	mmu-let-7d	0	chr13	48551775	48553275	678	+	10.0343
TCAACCATTTT	mmu-let-7e	20	chr17	17530726	17533550	558	-	9.69437
CCCGCCATCCT	mmu-let-7e	20	chr17	17530726	17533550	1094	-	9.67978
TTAGCCATTTT	mmu-let-7f-1	10	chr13	48551775	48553275	571	-	11.1306
ACATCCATCTT	mmu-let-7f-1	10	chr13	48551775	48553275	678	+	10.0343
TCCGCCATCTT	mmu-let-7g	25	chr9	106028929	106029129	118	-	12.535
GCCGCCATCTT	mmu-let-7i	10	chr10	122385250	122391850	3969	+	12.9448
GCCGCCATCTT	mmu-let-7i	10	chr10	122385250	122391850	4151	-	12.9448
TCATCCATCTT	mmu-let-7i	10	chr10	122385250	122391850	6096	+	10.2035
CCCGCCAGTTT	mmu-let-7i	10	chr10	122385250	122391850	4284	-	10.0812
TCCGCCATCTG	mmu-mir-106a	0	chrX	48985925	48991150	933	+	10.8013
CCCTCCATTCT	mmu-mir-106a	0	chrX	48985925	48991150	2719	+	9.55332
TCATCCATGTT	mmu-mir-122	0	chr18	65208050	65210300	1216	+	10.0408
CTGGCCATCTG	mmu-mir-122	0	chr18	65208050	65210300	1631	-	9.35387
TCCTCCATTTA	mmu-mir-124-1	0	chr14	63540450	63546275	5454	-	10.4772
TCAACCATTTT	mmu-mir-125a	20	chr17	17530726	17533550	558	-	9.69437
CCCGCCATCCT	mmu-mir-125a	20	chr17	17530726	17533550	1094	-	9.67978
TTCTCCATATT	mmu-mir-125b-2	9	chr16	77477393	77477593	50	-	10.8664
ATTTCCATCTT	mmu-mir-125b-2	10	chr16	77525525	77528275	1764	+	9.79412
TCAGCCATCTT	mmu-mir-127	-10	chr12	109987400	109991350	3285	-	11.0749
GCCGCCATGTC	mmu-mir-127	-10	chr12	109987400	109991350	1897	-	10.4694
CCCCCCATTTT	mmu-mir-127	-10	chr12	109987400	109991350	1932	-	10.2669
GCAGCCATCAT	mmu-mir-127	-10	chr12	109987400	109991350	2389	-	9.54513
TGAGCCATTTT	mmu-mir-127	-10	chr12	109987400	109991350	3241	-	9.43672

TCCTCCATCTT	mmu-mir-129-1	10	chr6	28970400	28972325	895	+	11.6637
TCCCCCATATT	mmu-mir-129-2	10	chr2	94041175	94045400	573	+	9.31003
GCCTCCATTTT	mmu-mir-130a	0	chr2	84542025	84546150	892	-	12.8183
CCGTCCATCTT	mmu-mir-130a	0	chr2	84542025	84546150	2555	+	10.9053
GCCACCATCTT	mmu-mir-130a	0	chr2	84542025	84546150	1463	+	10.8195
GCCCCCATTTT	mmu-mir-130a	0	chr2	84542025	84546150	3377	-	10.5729
GCTGCCATGTG	mmu-mir-130a	0	chr2	84542025	84546150	3505	+	10.0372
GCTACCATCTT	mmu-mir-130a	0	chr2	84542025	84546150	3562	-	9.80829
CTGGCCATTTC	mmu-mir-130a	0	chr2	84542025	84546150	514	-	9.51977
GGCTCCATCTT	mmu-mir-130b	30	chr16	17038569	17038769	111	+	9.69048
TTCTCCATTTT	mmu-mir-134	0	chr12	110043400	110045125	10	-	11.7194
TCAGCCATCTT	mmu-mir-136	-10	chr12	109987400	109991350	3285	-	11.0749
GCCGCCATGTC	mmu-mir-136	-10	chr12	109987400	109991350	1897	-	10.4694
CCCCCCATTTT	mmu-mir-136	-10	chr12	109987400	109991350	1932	-	10.2669
GCAGCCATCAT	mmu-mir-136	-10	chr12	109987400	109991350	2389	-	9.54513
TGAGCCATTTT	mmu-mir-136	-10	chr12	109987400	109991350	3241	-	9.43672
ACAGCCATTTG	mmu-mir-137	25	chr3	118425075	118425859	553	+	9.91675
TTCTCCATCTT	mmu-mir-138-1	-10	chr9	122416550	122422625	5494	-	10.9746
CCTGCCATTTG	mmu-mir-138-1	-10	chr9	122416550	122422625	2166	+	10.6388
CCCACCATCTT	mmu-mir-138-1	-10	chr9	122416550	122422625	1773	-	10.5135
CCTGCCATCTA	mmu-mir-138-1	-10	chr9	122416550	122422625	3619	+	9.69635
TACACCATTTT	mmu-mir-138-1	-10	chr9	122416550	122422625	5430	+	9.51667
GCCACCATTTA	mmu-mir-138-2	10	chr8	97212625	97214300	264	-	9.63303
GCAGCCATTTT	mmu-mir-141	20	chr6	124683151	124685075	229	+	12.2295
GAGGCCATCTT	mmu-mir-141	20	chr6	124683151	124685075	1526	+	10.4448
GCCTCCATCAT	mmu-mir-141	20	chr6	124683151	124685075	788	+	10.1339
GGGGCCATCTT	mmu-mir-141	20	chr6	124683151	124685075	380	+	9.69966
GCGGCCATGTT	mmu-mir-142	0	chr11	87571775	87574325	710	-	11.9199
GCCGCCATTTC	mmu-mir-142	0	chr11	87571775	87574325	716	+	11.377
GGCGCCATGTT	mmu-mir-142	0	chr11	87571775	87574325	1100	+	10.3991
GCCTCCATTTT	mmu-mir-143	20	chr18	61773267	61775675	2278	+	12.8183
GTGACCATCTT	mmu-mir-143	20	chr18	61773267	61775675	2035	+	9.26822
GCCTCCATTTT	mmu-mir-145	20	chr18	61773267	61775675	2278	+	12.8183
GTGACCATCTT	mmu-mir-145	20	chr18	61773267	61775675	2035	+	9.26822
CACTCCATATT	mmu-mir-146a	0	chr11	43227200	43230425	753	+	10.0215
GCAACCATCTT	mmu-mir-146a	0	chr11	43227200	43230425	2506	-	9.35934

GCGGCCATTTT	mmu-mir-146b	0	chr19	46390100	46393375	1993	+	12.8275
GTGGCCATTTT	mmu-mir-146b	0	chr19	46390100	46393375	1900	+	12.1384
CCTTCCATCTT	mmu-mir-148a	15	chr6	51198300	51201975	2181	+	10.7563
CCCCCCATTTT	mmu-mir-148a	15	chr6	51198300	51201975	919	-	10.2669
TTTTCCATCTT	mmu-mir-148a	15	chr6	51198300	51201975	3372	+	9.9634
GCCTCCATCTT	mmu-mir-148b	25	chr15	103100832	103101032	122	-	12.0735
TTTGCCATTTG	mmu-mir-149	19	chr1	94617259	94617459	7	+	9.84589
TTCTCCATTTT	mmu-mir-154	0	chr12	110043400	110045125	10	-	11.7194
CCTGCCATTTC	mmu-mir-155	0	chr16	84584475	84588475	2606	-	10.0598
GGCGCCATTTT	mmu-mir-15a	15	chr14	60602792	60602992	97	-	11.3067
ACCGCCATCTT	mmu-mir-15b	25	chr3	69092800	69093000	13	-	12.3657
GGCGCCATTTT	mmu-mir-15b	25	chr3	69092800	69093000	112	+	11.3067
GGCGCCATTTT	mmu-mir-16-1	15	chr14	60602792	60602992	97	-	11.3067
ACCGCCATCTT	mmu-mir-16-2	25	chr3	69092800	69093000	13	-	12.3657
GGCGCCATTTT	mmu-mir-16-2	25	chr3	69092800	69093000	112	+	11.3067
GCCGCCATGTT	mmu-mir-17	10	chr14	113921300	113927025	2442	+	12.7821
TTTGCCATCTT	mmu-mir-17	10	chr14	113921300	113927025	5563	-	10.8347
GCCACCATCTT	mmu-mir-17	10	chr14	113921300	113927025	1715	-	10.8195
GCCGCCATGTC	mmu-mir-17	10	chr14	113921300	113927025	2495	-	10.4694
TTCTCCATTTA	mmu-mir-17	10	chr14	113921300	113927025	518	-	9.78813
GCCGCCATCCT	mmu-mir-181a-2	0	chr1	139714910	139715099	18	-	9.98578
GCCGCCATCCT	mmu-mir-181b-1	0	chr1	139714910	139715099	18	-	9.98578
TCCTCCATTTT	mmu-mir-181c	10	chr8	87069525	87071900	997	-	12.4085
CCCGCCATGTG	mmu-mir-181c	10	chr8	87069525	87071900	1459	+	10.7424
CGGGCCATCTT	mmu-mir-181c	10	chr8	87069525	87071900	1039	+	9.39365
TCCTCCATTTT	mmu-mir-181d	10	chr8	87069525	87071900	997	-	12.4085
CCCGCCATGTG	mmu-mir-181d	10	chr8	87069525	87071900	1459	+	10.7424
CGGGCCATCTT	mmu-mir-181d	10	chr8	87069525	87071900	1039	+	9.39365
TCCTCCATTTT	mmu-mir-182	10	chr6	30114875	30130825	14432	+	12.4085
TCTTCCATTTT	mmu-mir-182	10	chr6	30114875	30130825	13040	-	11.3973
CCCACCATTTT	mmu-mir-182	10	chr6	30114875	30130825	1119	-	11.2583
TTTTCCATTTT	mmu-mir-182	10	chr6	30114875	30130825	12154	-	10.7083
CCCGCCATGTA	mmu-mir-182	10	chr6	30114875	30130825	13759	+	10.5448
GTGTCCATCTT	mmu-mir-182	10	chr6	30114875	30130825	5942	-	10.5222
GTCGCCATCTG	mmu-mir-182	10	chr6	30114875	30130825	4365	-	10.522
CCCTCCATCTG	mmu-mir-182	10	chr6	30114875	30130825	3959	+	10.0338

CCTTCCATTTG	mmu-mir-182	10	chr6	30114875	30130825	12707	-	9.76748
TCCTCCATCTA	mmu-mir-182	10	chr6	30114875	30130825	396	+	9.73236
GACTCCATTTG	mmu-mir-182	10	chr6	30114875	30130825	14586	+	9.44683
GTGTCCATTAT	mmu-mir-182	10	chr6	30114875	30130825	11716	+	9.32754
TCCTCCATTTT	mmu-mir-183	10	chr6	30114875	30130825	14432	+	12.4085
TCTTCCATTTT	mmu-mir-183	10	chr6	30114875	30130825	13040	-	11.3973
CCCACCATTTT	mmu-mir-183	10	chr6	30114875	30130825	1119	-	11.2583
TTTTCCATTTT	mmu-mir-183	10	chr6	30114875	30130825	12154	-	10.7083
CCCGCCATGTA	mmu-mir-183	10	chr6	30114875	30130825	13759	+	10.5448
GTGTCCATCTT	mmu-mir-183	10	chr6	30114875	30130825	5942	-	10.5222
GTCGCCATCTG	mmu-mir-183	10	chr6	30114875	30130825	4365	-	10.522
CCCTCCATCTG	mmu-mir-183	10	chr6	30114875	30130825	3959	+	10.0338
CCTTCCATTTG	mmu-mir-183	10	chr6	30114875	30130825	12707	-	9.76748
TCCTCCATCTA	mmu-mir-183	10	chr6	30114875	30130825	396	+	9.73236
GACTCCATTTG	mmu-mir-183	10	chr6	30114875	30130825	14586	+	9.44683
GTGTCCATTAT	mmu-mir-183	10	chr6	30114875	30130825	11716	+	9.32754
CCGGCCATCTT	mmu-mir-185	24	chr16	18261507	18261707	30	-	11.7767
GCCGCCATGTT	mmu-mir-18a	10	chr14	113921300	113927025	2442	+	12.7821
TTTGCCATCTT	mmu-mir-18a	10	chr14	113921300	113927025	5563	-	10.8347
GCCACCATCTT	mmu-mir-18a	10	chr14	113921300	113927025	1715	-	10.8195
GCCGCCATGTC	mmu-mir-18a	10	chr14	113921300	113927025	2495	-	10.4694
TTCTCCATTTA	mmu-mir-18a	10	chr14	113921300	113927025	518	-	9.78813
TCCGCCATCTG	mmu-mir-18b	0	chrX	48985925	48991150	933	+	10.8013
CCCTCCATTCT	mmu-mir-18b	0	chrX	48985925	48991150	2719	+	9.55332
TCCACCATGTT	mmu-mir-190b	-15	chr3	90134875	90141050	3924	+	10.2469
CTCTCCATTTA	mmu-mir-190b	-15	chr3	90134875	90141050	2357	+	9.89196
GCCTCCATGTC	mmu-mir-190b	-15	chr3	90134875	90141050	2634	+	9.59809
AGAGCCATTTT	mmu-mir-190b	-15	chr3	90134875	90141050	4533	-	9.26744
TCAGCCATTAT	mmu-mir-191	20	chr9	108424326	108426775	983	+	9.88016
GGAGCCATTTT	mmu-mir-191	20	chr9	108424326	108426775	1282	-	9.84655
GGGGCCATCTT	mmu-mir-192	-10	chr19	6247500	6249425	140	-	9.69966
CCTACCATCTT	mmu-mir-193	10	chr11	79526175	79531800	3098	+	9.50229
GTGGCCATGTG	mmu-mir-193	10	chr11	79526175	79531800	4941	+	9.49714
ATGACCATTTT	mmu-mir-193	10	chr11	79526175	79531800	3114	+	9.43397
TCTGCCATTTA	mmu-mir-193b	15	chr16	13359750	13363900	354	-	10.3374
CCAGCCATTTG	mmu-mir-193b	15	chr16	13359750	13363900	4043	+	10.1899

ACATCCATATT	mmu-mir-193b	15	chr16	13359750	13363900	2825	+	9.92607
ATTTCCATCTT	mmu-mir-193b	15	chr16	13359750	13363900	2947	-	9.79412
CTCCCCATTTT	mmu-mir-193b	15	chr16	13359750	13363900	2879	-	9.57782
GCTTCCATCTG	mmu-mir-193b	15	chr16	13359750	13363900	3829	+	9.32863
CCGGCCATGTC	mmu-mir-193b	15	chr16	13359750	13363900	1448	-	9.30126
TAGTCCATTTT	mmu-mir-194-1	0	chr1	186908300	186912075	623	-	9.90854
GGGGCCATCTT	mmu-mir-194-2	-10	chr19	6247500	6249425	140	-	9.69966
GCCTCCATTTC	mmu-mir-195	15	chr11	70048125	70050450	2295	+	10.5057
TCCTCCATTTT	mmu-mir-196a-1	-10	chr11	96075625	96077750	600	-	12.4085
GACACCATTTT	mmu-mir-196a-1	-10	chr11	96075625	96077750	1254	-	9.9265
TTGGCCATTTC	mmu-mir-196a-1	-10	chr11	96075625	96077750	1570	+	9.41594
CCCCCCATTTT	mmu-mir-196a-2	10	chr15	102799650	102802750	2756	-	10.2669
TTGTCCATGTT	mmu-mir-196a-2	10	chr15	102799650	102802750	1226	+	9.94968
CCATCCATTTG	mmu-mir-196a-2	10	chr15	102799650	102802750	278	-	9.31853
CCAGCCATTTT	mmu-mir-196b	10	chr6	52159743	52171275	5872	+	11.9235
CCTTCCATTTT	mmu-mir-196b	10	chr6	52159743	52171275	986	-	11.5012
CCTGCCATTTG	mmu-mir-196b	10	chr6	52159743	52171275	6231	-	10.6388
CTGACCATTTT	mmu-mir-196b	10	chr6	52159743	52171275	6007	-	9.70708
CTCGCCATCTC	mmu-mir-196b	10	chr6	52159743	52171275	2222	-	9.63706
CTCGCCATGTC	mmu-mir-196b	10	chr6	52159743	52171275	5848	-	9.47432
CTGGCCATATT	mmu-mir-199a-1	10	chr9	21244900	21248875	1285	+	10.9794
GCTGCCATCTC	mmu-mir-199a-1	10	chr9	21244900	21248875	2407	-	9.62098
TATTCCATTTT	mmu-mir-199a-2	10	chr1	164052250	164061375	6460	+	9.75952
GCTACCATGTT	mmu-mir-199a-2	10	chr1	164052250	164061375	8571	+	9.64556
CACACCATTTT	mmu-mir-199a-2	10	chr1	164052250	164061375	4474	+	9.62049
GCAGCCATTCT	mmu-mir-199a-2	10	chr1	164052250	164061375	7307	-	9.27053
CCAGCCATTTG	mmu-mir-199b	15	chr2	32138775	32141975	2072	-	10.1899
ACTGCCATAAT	mmu-mir-199b	9	chr2	32136375	32138625	1178	+	9.30678
GCCGCCATGTT	mmu-mir-19a	10	chr14	113921300	113927025	2442	+	12.7821
TTTGCCATCTT	mmu-mir-19a	10	chr14	113921300	113927025	5563	-	10.8347
GCCACCATCTT	mmu-mir-19a	10	chr14	113921300	113927025	1715	-	10.8195
GCCGCCATGTC	mmu-mir-19a	10	chr14	113921300	113927025	2495	-	10.4694
TTCTCCATTTA	mmu-mir-19a	10	chr14	113921300	113927025	518	-	9.78813
TCCGCCATCTG	mmu-mir-19b	0	chrX	48985925	48991150	933	+	10.8013
CCCTCCATTCT	mmu-mir-19b	0	chrX	48985925	48991150	2719	+	9.55332
GCAGCCATTTT	mmu-mir-200c	20	chr6	124683151	124685075	229	+	12.2295

GAGGCCATCTT	mmu-mir-200c	20	chr6	124683151	124685075	1526	+	10.4448
GCCTCCATCAT	mmu-mir-200c	20	chr6	124683151	124685075	788	+	10.1339
GGGGCCATCTT	mmu-mir-200c	20	chr6	124683151	124685075	380	+	9.69966
GCTGCCATTAT	mmu-mir-202	0	chr7	139821975	139823300	135	+	10.7389
CCATCCATCTT	mmu-mir-202	0	chr7	139821975	139823300	121	+	10.3074
ACTTCCATTAT	mmu-mir-205	0	chr1	195208350	195211700	1924	+	9.2885
GCCGCCATGTT	mmu-mir-20a	10	chr14	113921300	113927025	2442	+	12.7821
TTTGCCATCTT	mmu-mir-20a	10	chr14	113921300	113927025	5563	-	10.8347
GCCACCATCTT	mmu-mir-20a	10	chr14	113921300	113927025	1715	-	10.8195
GCCGCCATGTC	mmu-mir-20a	10	chr14	113921300	113927025	2495	-	10.4694
TTCTCCATTTA	mmu-mir-20a	10	chr14	113921300	113927025	518	-	9.78813
TCCGCCATCTG	mmu-mir-20b	0	chrX	48985925	48991150	933	+	10.8013
CCCTCCATTCT	mmu-mir-20b	0	chrX	48985925	48991150	2719	+	9.55332
TATTCCATTTT	mmu-mir-214	10	chr1	164052250	164061375	6460	+	9.75952
GCTACCATGTT	mmu-mir-214	10	chr1	164052250	164061375	8571	+	9.64556
CACACCATTTT	mmu-mir-214	10	chr1	164052250	164061375	4474	+	9.62049
GCAGCCATTCT	mmu-mir-214	10	chr1	164052250	164061375	7307	-	9.27053
TAGTCCATTTT	mmu-mir-215	0	chr1	186908300	186912075	623	-	9.90854
CCCTCCATCTC	mmu-mir-216a	-10	chr11	28482175	28485250	2221	+	9.45483
CCCTCCATCTC	mmu-mir-216b	-10	chr11	28482175	28485250	2221	+	9.45483
CCCTCCATCTC	mmu-mir-217	-10	chr11	28482175	28485250	2221	+	9.45483
GCCGCCATCCT	mmu-mir-219-2	15	chr2	29666575	29669250	937	-	9.98578
CCTGCCATCTA	mmu-mir-220	0	chr6	136427625	136429100	897	+	9.69635
CTTGCCATTTT	mmu-mir-221	-10	chrX	18319475	18326525	2	-	11.6834
ATCTCCATTTT	mmu-mir-221	-10	chrX	18319475	18326525	2027	-	11.5501
GCTTCCATTTG	mmu-mir-221	-10	chrX	18319475	18326525	4301	+	10.0735
TATTCCATTTT	mmu-mir-221	-10	chrX	18319475	18326525	6558	-	9.75952
ATCTCCATTTA	mmu-mir-221	-10	chrX	18319475	18326525	3299	-	9.61885
CTTGCCATTTT	mmu-mir-222	-10	chrX	18319475	18326525	2	-	11.6834
ATCTCCATTTT	mmu-mir-222	-10	chrX	18319475	18326525	2027	-	11.5501
GCTTCCATTTG	mmu-mir-222	-10	chrX	18319475	18326525	4301	+	10.0735
TATTCCATTTT	mmu-mir-222	-10	chrX	18319475	18326525	6558	-	9.75952
ATCTCCATTTA	mmu-mir-222	-10	chrX	18319475	18326525	3299	-	9.61885
GCGGCCATTTG	mmu-mir-23a	0	chr8	87086300	87095525	7991	-	11.0938
CATTCCATTTT	mmu-mir-23a	0	chr8	87086300	87095525	2967	-	9.86335
CTCCCCATTTT	mmu-mir-23a	0	chr8	87086300	87095525	2685	+	9.57782

ACTGCCATGTG	mmu-mir-23a	0	chr8	87086300	87095525	6995	-	9.4581
GCGGCCATTTG	mmu-mir-24-2	0	chr8	87086300	87095525	7991	-	11.0938
CATTCCATTTT	mmu-mir-24-2	0	chr8	87086300	87095525	2967	-	9.86335
CTCCCCATTTT	mmu-mir-24-2	0	chr8	87086300	87095525	2685	+	9.57782
ACTGCCATGTG	mmu-mir-24-2	0	chr8	87086300	87095525	6995	-	9.4581
GCGGCCATTTG	mmu-mir-27a	0	chr8	87086300	87095525	7991	-	11.0938
CATTCCATTTT	mmu-mir-27a	0	chr8	87086300	87095525	2967	-	9.86335
CTCCCCATTTT	mmu-mir-27a	0	chr8	87086300	87095525	2685	+	9.57782
ACTGCCATGTG	mmu-mir-27a	0	chr8	87086300	87095525	6995	-	9.4581
ACAGCCATTTT	mmu-mir-296	-15	chr2	173925825	173933325	3722	+	11.6504
TTGGCCATCTT	mmu-mir-296	-15	chr2	173925825	173933325	3731	+	10.9837
AAGGCCATTTT	mmu-mir-296	-15	chr2	173925825	173933325	5083	+	10.6106
CTCGCCATAAT	mmu-mir-296	-15	chr2	173925825	173933325	1824	+	9.90197
GATGCCATTTG	mmu-mir-297a-6	0	chr2	169051150	169052700	93	+	9.30699
TCTACCATATT	mmu-mir-297a-6	0	chr2	169051150	169052700	788	+	9.29027
TACTCCATTTT	mmu-mir-297a-7	-10	chr10	42708700	42711800	2886	-	10.7707
ATGTCCATTTT	mmu-mir-297a-7	-10	chr10	42708700	42711800	2517	+	10.688
CCCGCCATCTC	mmu-mir-297a-7	-10	chr10	42708700	42711800	26	+	10.3261
ACAGCCATTTT	mmu-mir-298	-15	chr2	173925825	173933325	3722	+	11.6504
TTGGCCATCTT	mmu-mir-298	-15	chr2	173925825	173933325	3731	+	10.9837
AAGGCCATTTT	mmu-mir-298	-15	chr2	173925825	173933325	5083	+	10.6106
CTCGCCATAAT	mmu-mir-298	-15	chr2	173925825	173933325	1824	+	9.90197
TTCTCCATTTT	mmu-mir-299	0	chr12	110043400	110045125	10	-	11.7194
CCCTCCATTCT	mmu-mir-29a	0	chr6	31006975	31008175	502	-	9.55332
CCCTCCATTCT	mmu-mir-29b-1	0	chr6	31006975	31008175	502	-	9.55332
ACGGCCATTTT	mmu-mir-29b-2	-10	chr1	196676100	196679875	3601	+	12.2484
CTCTCCATGTT	mmu-mir-29b-2	-10	chr1	196676100	196679875	3197	-	10.9157
TTGTCCATTTT	mmu-mir-29b-2	-10	chr1	196676100	196679875	3005	+	10.8573
GCCACCATGTT	mmu-mir-29b-2	-10	chr1	196676100	196679875	2287	+	10.6567
TCTGCCATTTG	mmu-mir-29b-2	-10	chr1	196676100	196679875	1899	-	10.535
GCCTCCATCTG	mmu-mir-29b-2	-10	chr1	196676100	196679875	2071	+	10.3398
CTTTCCATCTT	mmu-mir-29b-2	-10	chr1	196676100	196679875	206	+	10.0672
TTCTCCATTTC	mmu-mir-29b-2	-10	chr1	196676100	196679875	2534	+	9.40677
ACGGCCATTTT	mmu-mir-29c	-10	chr1	196676100	196679875	3601	+	12.2484
CTCTCCATGTT	mmu-mir-29c	-10	chr1	196676100	196679875	3197	-	10.9157
TTGTCCATTTT	mmu-mir-29c	-10	chr1	196676100	196679875	3005	+	10.8573

GCCACCATGTT	mmu-mir-29c	-10	chr1	196676100	196679875	2287	+	10.6567
TCTGCCATTTG	mmu-mir-29c	-10	chr1	196676100	196679875	1899	-	10.535
GCCTCCATCTG	mmu-mir-29c	-10	chr1	196676100	196679875	2071	+	10.3398
CTTTCCATCTT	mmu-mir-29c	-10	chr1	196676100	196679875	206	+	10.0672
TTCTCCATTTC	mmu-mir-29c	-10	chr1	196676100	196679875	2534	+	9.40677
TTCTCCATTTT	mmu-mir-300	0	chr12	110043400	110045125	10	-	11.7194
GCCTCCATATT	mmu-mir-301a	25	chr11	86925352	86925552	111	-	11.9653
GGCTCCATCTT	mmu-mir-301b	30	chr16	17038569	17038769	111	+	9.69048
GCCTCCATGTT	mmu-mir-302a	10	chr3	127537000	127537941	655	-	11.9107
GCTTCCATGTT	mmu-mir-302a	10	chr3	127537000	127537941	691	+	10.8996
GCTTCCATGTT	mmu-mir-302a	10	chr3	127537000	127537941	301	+	10.8996
GCTTCCATGTT	mmu-mir-302a	10	chr3	127537000	127537941	431	+	10.8996
GCTTCCATGTT	mmu-mir-302a	10	chr3	127537000	127537941	565	+	10.8996
GCCTCCATGTT	mmu-mir-302b	10	chr3	127537000	127537941	655	-	11.9107
GCTTCCATGTT	mmu-mir-302b	10	chr3	127537000	127537941	691	+	10.8996
GCTTCCATGTT	mmu-mir-302b	10	chr3	127537000	127537941	565	+	10.8996
GCTTCCATGTT	mmu-mir-302b	10	chr3	127537000	127537941	431	+	10.8996
GCTTCCATGTT	mmu-mir-302b	10	chr3	127537000	127537941	301	+	10.8996
GCCTCCATGTT	mmu-mir-302c	10	chr3	127537000	127537941	655	-	11.9107
GCTTCCATGTT	mmu-mir-302c	10	chr3	127537000	127537941	691	+	10.8996
GCTTCCATGTT	mmu-mir-302c	10	chr3	127537000	127537941	565	+	10.8996
GCTTCCATGTT	mmu-mir-302c	10	chr3	127537000	127537941	431	+	10.8996
GCTTCCATGTT	mmu-mir-302c	10	chr3	127537000	127537941	301	+	10.8996
GCCTCCATGTT	mmu-mir-302d	10	chr3	127537000	127537941	655	-	11.9107
GCTTCCATGTT	mmu-mir-302d	10	chr3	127537000	127537941	301	+	10.8996
GCTTCCATGTT	mmu-mir-302d	10	chr3	127537000	127537941	431	+	10.8996
GCTTCCATGTT	mmu-mir-302d	10	chr3	127537000	127537941	691	+	10.8996
GCTTCCATGTT	mmu-mir-302d	10	chr3	127537000	127537941	565	+	10.8996
GTCTCCATTTT	mmu-mir-30b	-8	chr15	68190175	68194550	560	-	12.1293
CTCTCCATATT	mmu-mir-30b	-8	chr15	68190175	68194550	2971	-	10.9702
ATAGCCATATT	mmu-mir-30b	-8	chr15	68190175	68194550	96	+	10.1083
TAGGCCATCTT	mmu-mir-30b	-8	chr15	68190175	68194550	914	-	10.035
TTGTCCATATT	mmu-mir-30b	-8	chr15	68190175	68194550	54	-	10.0042
GCCGCCATCTT	mmu-mir-30c-1	14	chr4	120323146	120323346	164	-	12.9448
GCCGCCATCTT	mmu-mir-30e	14	chr4	120323146	120323346	164	-	12.9448
ACAGCCATCTT	mmu-mir-31	0	chr4	88399300	88401975	1054	-	10.9056

GCTGCCATCTT	mmu-mir-320	2	chr14	69176250	69180100	2268	+	11.9336
GGCGCCATTTT	mmu-mir-322	0	chrX	49298875	49304275	2424	-	11.3067
GTGTCCATTTT	mmu-mir-322	0	chrX	49298875	49304275	780	-	11.2671
GGGGCCATTTT	mmu-mir-322	0	chrX	49298875	49304275	1729	-	10.4445
CCGGCCATTTC	mmu-mir-322	0	chrX	49298875	49304275	732	-	10.2089
TTCTCCATTTT	mmu-mir-323	0	chr12	110043400	110045125	10	-	11.7194
CCCGCCATCCT	mmu-mir-324	20	chr11	69816711	69816911	172	+	9.67978
TTCGCCATATG	mmu-mir-327	-10	chr14	43909450	43912875	2207	+	10.004
CTGGCCATTAT	mmu-mir-327	-10	chr14	43909450	43912875	2186	+	9.89286
GCAGCCATCTT	mmu-mir-328	-15	chr8	108213550	108217600	1565	+	11.4847
CTATCCATTTT	mmu-mir-328	-15	chr8	108213550	108217600	1170	-	10.3631
ATCACCATATT	mmu-mir-328	-15	chr8	108213550	108217600	330	+	9.44307
TTCTCCATTTT	mmu-mir-329	0	chr12	110043400	110045125	10	-	11.7194
GCCGCCATGTT	mmu-mir-33	17	chr15	81974358	81974558	163	-	12.7821
TCTGCCATCTT	mmu-mir-331	0	chr10	93443775	93445175	925	-	11.5238
TTCTCCATGTT	mmu-mir-331	0	chr10	93443775	93445175	904	-	10.8118
GCGGCCATCTC	mmu-mir-331	0	chr10	93443775	93445175	1250	-	9.77
TCAGCCATCTT	mmu-mir-337	-10	chr12	109987400	109991350	3285	-	11.0749
GCCGCCATGTC	mmu-mir-337	-10	chr12	109987400	109991350	1897	-	10.4694
CCCCCCATTTT	mmu-mir-337	-10	chr12	109987400	109991350	1932	-	10.2669
GCAGCCATCAT	mmu-mir-337	-10	chr12	109987400	109991350	2389	-	9.54513
TGAGCCATTTT	mmu-mir-337	-10	chr12	109987400	109991350	3241	-	9.43672
GCGTCCATCTT	mmu-mir-339	22	chr5	139714076	139714276	143	+	11.2113
TTGGCCATCTT	mmu-mir-339	10	chr5	139624300	139627075	2468	+	10.9837
ATCTCCATATT	mmu-mir-339	10	chr5	139624300	139627075	2160	-	10.6971
GTGTCCATGTT	mmu-mir-339	10	chr5	139624300	139627075	1266	+	10.3595
TTCTCCATTTT	mmu-mir-341	0	chr12	110043400	110045125	10	-	11.7194
GCCTCCATTTC	mmu-mir-344-1	0	chr7	61818450	61820325	977	+	10.5057
GTTGCCATCAT	mmu-mir-344-1	0	chr7	61818450	61820325	1686	-	9.30499
GCCTCCATTTC	mmu-mir-344-2	10	chr7	61818450	61820325	977	+	10.5057
GTTGCCATCAT	mmu-mir-344-2	10	chr7	61818450	61820325	1686	-	9.30499
GCTTCCATATT	mmu-mir-34a	0	chr4	148890450	148895975	1834	+	10.9541
ACAGCCATTTG	mmu-mir-34a	0	chr4	148890450	148895975	4352	-	9.91674
CCCTCCATTCT	mmu-mir-34a	0	chr4	148890450	148895975	3983	+	9.55332
TCCTCCATCTC	mmu-mir-34a	0	chr4	148890450	148895975	3637	-	9.351
TGCGCCATCTT	mmu-mir-34b	20	chr9	50855701	50858400	1475	+	10.152

TCCTCCATGAT	mmu-mir-34b	20	chr9	50855701	50858400	1279	-	9.56135
TGCGCCATCTT	mmu-mir-34c	20	chr9	50855701	50858400	1475	+	10.152
TCCTCCATGAT	mmu-mir-34c	20	chr9	50855701	50858400	1279	-	9.56135
CTAGCCATGTT	mmu-mir-350	0	chr1	178636600	178640175	2758	-	10.3269
AATTCCATTTT	mmu-mir-350	0	chr1	178636600	178640175	3020	-	9.59024
TCTGCCATATA	mmu-mir-350	0	chr1	178636600	178640175	1363	+	9.48433
TTATCCATATT	mmu-mir-350	0	chr1	178636600	178640175	1833	-	9.40626
GCAGCCATTTT	mmu-mir-351	10	chrX	49296350	49298075	1662	+	12.2295
ATTGCCATCTT	mmu-mir-351	10	chrX	49296350	49298075	531	+	10.6654
CTAGCCATATT	mmu-mir-351	10	chrX	49296350	49298075	94	-	10.3814
TTAGCCATTTG	mmu-mir-351	10	chrX	49296350	49298075	1043	+	9.39694
TCCGCCATCTT	mmu-mir-361	20	chrX	109302359	109302559	66	+	12.535
TCCGCCATCTG	mmu-mir-363	0	chrX	48985925	48991150	933	+	10.8013
CCCTCCATTCT	mmu-mir-363	0	chrX	48985925	48991150	2719	+	9.55332
TCTGCCATTTA	mmu-mir-365-1	6	chr16	13359750	13363900	354	-	10.3374
CCAGCCATTTG	mmu-mir-365-1	6	chr16	13359750	13363900	4043	+	10.1899
ACATCCATATT	mmu-mir-365-1	6	chr16	13359750	13363900	2825	+	9.92607
ATTTCCATCTT	mmu-mir-365-1	6	chr16	13359750	13363900	2947	-	9.79412
CTCCCCATTTT	mmu-mir-365-1	6	chr16	13359750	13363900	2879	-	9.57782
GCTTCCATCTG	mmu-mir-365-1	6	chr16	13359750	13363900	3829	+	9.32863
CCGGCCATGTC	mmu-mir-365-1	6	chr16	13359750	13363900	1448	-	9.30126
CCTACCATCTT	mmu-mir-365-2	0	chr11	79526175	79531800	3098	+	9.50229
GTGGCCATGTG	mmu-mir-365-2	0	chr11	79526175	79531800	4941	+	9.49714
ATGACCATTTT	mmu-mir-365-2	0	chr11	79526175	79531800	3114	+	9.43397
GCCTCCATGTT	mmu-mir-367	10	chr3	127537000	127537941	655	-	11.9107
GCTTCCATGTT	mmu-mir-367	10	chr3	127537000	127537941	301	+	10.8996
GCTTCCATGTT	mmu-mir-367	10	chr3	127537000	127537941	431	+	10.8996
GCTTCCATGTT	mmu-mir-367	10	chr3	127537000	127537941	565	+	10.8996
GCTTCCATGTT	mmu-mir-367	10	chr3	127537000	127537941	691	+	10.8996
TTCTCCATTTT	mmu-mir-368	0	chr12	110043400	110045125	10	-	11.7194
TTCTCCATTTT	mmu-mir-369	0	chr12	110043400	110045125	10	-	11.7194
TTCTCCATTTT	mmu-mir-370	0	chr12	110043400	110045125	10	-	11.7194
ACTTCCATCTT	mmu-mir-374	0	chrX	99817575	99820975	2397	-	10.4832
CTGACCATTTT	mmu-mir-374	0	chrX	99817575	99820975	489	+	9.70708
TCAACCATTTT	mmu-mir-374	0	chrX	99817575	99820975	2254	-	9.69437
TTCTCCATTTT	mmu-mir-376a	0	chr12	110043400	110045125	10	-	11.7194

TTCTCCATTTT	mmu-mir-376b	0	chr12	110043400	110045125	10	-	11.7194
TTCTCCATTTT	mmu-mir-377	0	chr12	110043400	110045125	10	-	11.7194
CCCGCCATCTT	mmu-mir-378	35	chr18	61525700	61525900	28	+	12.6388
TTCTCCATTTT	mmu-mir-379	0	chr12	110043400	110045125	10	-	11.7194
TTCTCCATTTT	mmu-mir-380	0	chr12	110043400	110045125	10	-	11.7194
TTCTCCATTTT	mmu-mir-381	0	chr12	110043400	110045125	10	-	11.7194
TTCTCCATTTT	mmu-mir-382	0	chr12	110043400	110045125	10	-	11.7194
GCGGCCATTTT	mmu-mir-384	-10	chrX	101592850	101595950	312	-	12.8275
CTGGCCATTTT	mmu-mir-384	-10	chrX	101592850	101595950	1518	+	11.8324
CCCTCCATCTT	mmu-mir-384	-10	chrX	101592850	101595950	549	+	11.7675
TCAGCCATCTT	mmu-mir-384	-10	chrX	101592850	101595950	1379	+	11.0749
GCCGCCATCCT	mmu-mir-384	-10	chrX	101592850	101595950	318	+	9.98579
ATGGCCATTTG	mmu-mir-384	-10	chrX	101592850	101595950	988	+	9.82562
TTCTCCATTTT	mmu-mir-409	0	chr12	110043400	110045125	10	-	11.7194
TTCTCCATTTT	mmu-mir-410	0	chr12	110043400	110045125	10	-	11.7194
TTCTCCATTTT	mmu-mir-411	0	chr12	110043400	110045125	10	-	11.7194
TTCTCCATTTT	mmu-mir-412	0	chr12	110043400	110045125	10	-	11.7194
ATCGCCATTTT	mmu-mir-421	0	chrX	99822432	99826775	2580	-	12.4215
TCGGCCATTTT	mmu-mir-421	0	chrX	99822432	99826775	1296	+	12.4177
CCCTCCATGTT	mmu-mir-421	0	chrX	99822432	99826775	3794	+	11.6047
TAGGCCATCTT	mmu-mir-421	0	chrX	99822432	99826775	1074	-	10.035
TCTTCCATTTG	mmu-mir-421	0	chrX	99822432	99826775	1395	+	9.66365
TTATCCATGTT	mmu-mir-421	0	chrX	99822432	99826775	310	+	9.35171
ATCCCCATTTT	mmu-mir-421	0	chrX	99822432	99826775	1164	-	9.30471
ATCGCCATCTT	mmu-mir-423	35	chr11	76894530	76894730	55	+	11.6766
TCAGCCATTAT	mmu-mir-425	20	chr9	108424326	108426775	983	+	9.88016
GGAGCCATTTT	mmu-mir-425	20	chr9	108424326	108426775	1282	-	9.84655
TCAGCCATCTT	mmu-mir-431	-10	chr12	109987400	109991350	3285	-	11.0749
GCCGCCATGTC	mmu-mir-431	-10	chr12	109987400	109991350	1897	-	10.4694
CCCCCCATTTT	mmu-mir-431	-10	chr12	109987400	109991350	1932	-	10.2669
GCAGCCATCAT	mmu-mir-431	-10	chr12	109987400	109991350	2389	-	9.54513
TGAGCCATTTT	mmu-mir-431	-10	chr12	109987400	109991350	3241	-	9.43672
TCAGCCATCTT	mmu-mir-433	-10	chr12	109987400	109991350	3285	-	11.0749
GCCGCCATGTC	mmu-mir-433	-10	chr12	109987400	109991350	1897	-	10.4694
CCCCCCATTTT	mmu-mir-433	-10	chr12	109987400	109991350	1932	-	10.2669
GCAGCCATCAT	mmu-mir-433	-10	chr12	109987400	109991350	2389	-	9.54513

TGAGCCATTTT	mmu-mir-433	-10	chr12	109987400	109991350	3241	-	9.43672
TCAGCCATCTT	mmu-mir-434	-10	chr12	109987400	109991350	3285	-	11.0749
GCCGCCATGTC	mmu-mir-434	-10	chr12	109987400	109991350	1897	-	10.4694
CCCCCCATTTT	mmu-mir-434	-10	chr12	109987400	109991350	1932	-	10.2669
GCAGCCATCAT	mmu-mir-434	-10	chr12	109987400	109991350	2389	-	9.54513
TGAGCCATTTT	mmu-mir-434	-10	chr12	109987400	109991350	3241	-	9.43672
CCTGCCATATG	mmu-mir-449a	15	chr13	114154825	114157525	435	+	9.78576
CCTGCCATATG	mmu-mir-449b	15	chr13	114154825	114157525	435	+	9.78576
GCAGCCATTTT	mmu-mir-450a-1	0	chrX	49296350	49298075	1662	+	12.2295
ATTGCCATCTT	mmu-mir-450a-1	0	chrX	49296350	49298075	531	+	10.6654
CTAGCCATATT	mmu-mir-450a-1	0	chrX	49296350	49298075	94	-	10.3814
TTAGCCATTTG	mmu-mir-450a-1	0	chrX	49296350	49298075	1043	+	9.39694
GCAGCCATTTT	mmu-mir-450a-2	0	chrX	49296350	49298075	1662	+	12.2295
ATTGCCATCTT	mmu-mir-450a-2	0	chrX	49296350	49298075	531	+	10.6654
CTAGCCATATT	mmu-mir-450a-2	0	chrX	49296350	49298075	94	-	10.3814
TTAGCCATTTG	mmu-mir-450a-2	0	chrX	49296350	49298075	1043	+	9.39694
GCAGCCATTTT	mmu-mir-450b	0	chrX	49296350	49298075	1662	+	12.2295
ATTGCCATCTT	mmu-mir-450b	0	chrX	49296350	49298075	531	+	10.6654
CTAGCCATATT	mmu-mir-450b	0	chrX	49296350	49298075	94	-	10.3814
TTAGCCATTTG	mmu-mir-450b	0	chrX	49296350	49298075	1043	+	9.39694
TTCTCCATTTT	mmu-mir-453	0	chr12	110043400	110045125	10	-	11.7194
ACTTCCATTTT	mmu-mir-484	2	chr16	14070875	14074575	17	+	11.2281
TCCTCCATAAT	mmu-mir-484	2	chr16	14070875	14074575	2066	+	9.6159
TTCTCCATTTT	mmu-mir-485	0	chr12	110043400	110045125	10	-	11.7194
TTCTCCATTTT	mmu-mir-487b	0	chr12	110043400	110045125	10	-	11.7194
TCAGCCATCTT	mmu-mir-493	-10	chr12	109987400	109991350	3285	-	11.0749
GCCGCCATGTC	mmu-mir-493	-10	chr12	109987400	109991350	1897	-	10.4694
CCCCCCATTTT	mmu-mir-493	-10	chr12	109987400	109991350	1932	-	10.2669
GCAGCCATCAT	mmu-mir-493	-10	chr12	109987400	109991350	2389	-	9.54513
TGAGCCATTTT	mmu-mir-493	-10	chr12	109987400	109991350	3241	-	9.43672
TTCTCCATTTT	mmu-mir-494	0	chr12	110043400	110045125	10	-	11.7194
TTCTCCATTTT	mmu-mir-495	0	chr12	110043400	110045125	10	-	11.7194
TTCTCCATTTT	mmu-mir-496	0	chr12	110043400	110045125	10	-	11.7194
GCCTCCATTTC	mmu-mir-497	15	chr11	70048125	70050450	2295	+	10.5057
GGCGCCATTTT	mmu-mir-503	0	chrX	49298875	49304275	2424	-	11.3067
GTGTCCATTTT	mmu-mir-503	0	chrX	49298875	49304275	780	-	11.2671

GGGGCCATTTT	mmu-mir-503	0	chrX	49298875	49304275	1729	-	10.4445
CCGGCCATTTC	mmu-mir-503	0	chrX	49298875	49304275	732	-	10.2089
TTCTCCATTTT	mmu-mir-539	0	chr12	110043400	110045125	10	-	11.7194
TCAGCCATCTT	mmu-mir-540	-10	chr12	109987400	109991350	3285	-	11.0749
GCCGCCATGTC	mmu-mir-540	-10	chr12	109987400	109991350	1897	-	10.4694
CCCCCCATTTT	mmu-mir-540	-10	chr12	109987400	109991350	1932	-	10.2669
GCAGCCATCAT	mmu-mir-540	-10	chr12	109987400	109991350	2389	-	9.54513
TGAGCCATTTT	mmu-mir-540	-10	chr12	109987400	109991350	3241	-	9.43672
TTCTCCATTTT	mmu-mir-541	0	chr12	110043400	110045125	10	-	11.7194
GCAGCCATTTT	mmu-mir-542	10	chrX	49296350	49298075	1662	+	12.2295
ATTGCCATCTT	mmu-mir-542	10	chrX	49296350	49298075	531	+	10.6654
CTAGCCATATT	mmu-mir-542	10	chrX	49296350	49298075	94	-	10.3814
TTAGCCATTTG	mmu-mir-542	10	chrX	49296350	49298075	1043	+	9.39694
TTCTCCATTTT	mmu-mir-543	0	chr12	110043400	110045125	10	-	11.7194
TTCTCCATTTT	mmu-mir-544	0	chr12	110043400	110045125	10	-	11.7194
GCTGCCATTCT	mmu-mir-568	-10	chr16	43528050	43530275	1166	-	9.71948
GCCGCCATCTT	mmu-mir-598	0	chr14	62559175	62563900	1461	-	12.9448
GCGGCCATCTT	mmu-mir-598	0	chr14	62559175	62563900	1217	+	12.0827
ACAGCCATTTT	mmu-mir-598	0	chr14	62559175	62563900	834	+	11.6504
TTCTCCATTTT	mmu-mir-654	0	chr12	110043400	110045125	10	-	11.7194
TCAGCCATCTT	mmu-mir-665	-10	chr12	109987400	109991350	3285	-	11.0749
GCCGCCATGTC	mmu-mir-665	-10	chr12	109987400	109991350	1897	-	10.4694
CCCCCCATTTT	mmu-mir-665	-10	chr12	109987400	109991350	1932	-	10.2669
GCAGCCATCAT	mmu-mir-665	-10	chr12	109987400	109991350	2389	-	9.54513
TGAGCCATTTT	mmu-mir-665	-10	chr12	109987400	109991350	3241	-	9.43672
TTCTCCATTTT	mmu-mir-666	0	chr12	110043400	110045125	10	-	11.7194
TCAGCCATCTT	mmu-mir-673	-10	chr12	109987400	109991350	3285	-	11.0749
GCCGCCATGTC	mmu-mir-673	-10	chr12	109987400	109991350	1897	-	10.4694
CCCCCCATTTT	mmu-mir-673	-10	chr12	109987400	109991350	1932	-	10.2669
GCAGCCATCAT	mmu-mir-673	-10	chr12	109987400	109991350	2389	-	9.54513
TGAGCCATTTT	mmu-mir-673	-10	chr12	109987400	109991350	3241	-	9.43672
GCCTCCATTTG	mmu-mir-674	0	chr2	116827650	116829025	484	+	11.0847
GCCGCCATTGT	mmu-mir-744	25	chr11	65604392	65604592	39	+	10.2232
TTCTCCATTTT	mmu-mir-758	0	chr12	110043400	110045125	10	-	11.7194
TTGGCCATTTT	mmu-mir-760	22	chr3	122285704	122287675	1256	-	11.7286
TCAGCCATCTT	mmu-mir-770	-10	chr12	109987400	109991350	3285	-	11.0749

GCCGCCATGTC	mmu-mir-770	-10	chr12	109987400	109991350	1897	-	10.4694
CCCCCCATTTT	mmu-mir-770	-10	chr12	109987400	109991350	1932	-	10.2669
GCAGCCATCAT	mmu-mir-770	-10	chr12	109987400	109991350	2389	-	9.54513
TGAGCCATTTT	mmu-mir-770	-10	chr12	109987400	109991350	3241	-	9.43672
CTTTCCATTTT	mmu-mir-873	0	chr4	37072975	37074125	460	+	10.8121
GTAGCCATGTT	mmu-mir-873	0	chr4	37072975	37074125	162	-	10.6329
ACTTCCATTTT	mmu-mir-875	0	chr15	35693875	35694875	374	-	11.2281
CTTTCCATTTT	mmu-mir-876	0	chr4	37072975	37074125	460	+	10.8121
GTAGCCATGTT	mmu-mir-876	0	chr4	37072975	37074125	162	-	10.6329
TTCTCCATTTT	mmu-mir-882	0	chr12	110043400	110045125	10	-	11.7194
TCCGCCATCTG	mmu-mir-92a	0	chrX	48985925	48991150	933	+	10.8013
CCCTCCATTCT	mmu-mir-92a	0	chrX	48985925	48991150	2719	+	9.55332
ATGGCCATTTA	mmu-mir-92b	20	chr3	89313125	89315675	2071	-	9.62803
CCTGCCATCTT	mmu-mir-9-3	10	chr7	79377250	79382900	506	-	11.6276
ACCTCCATCTT	mmu-mir-9-3	10	chr7	79377250	79382900	3210	+	11.4944
GGCGCCATTTT	mmu-mir-9-3	10	chr7	79377250	79382900	5327	+	11.3067
CCATCCATTTT	mmu-mir-9-3	10	chr7	79377250	79382900	4254	+	11.0522
GTTGCCATTTA	mmu-mir-9-3	10	chr7	79377250	79382900	2606	-	10.0581
TTTTCCATCTT	mmu-mir-9-3	10	chr7	79377250	79382900	3324	+	9.9634
CCGGCCATATG	mmu-mir-9-3	10	chr7	79377250	79382900	4738	+	9.93477
TTGGCCATTTA	mmu-mir-9-3	10	chr7	79377250	79382900	5443	-	9.79731
TCCTCCATTTT	mmu-mir-96	10	chr6	30114875	30130825	14432	+	12.4085
TCTTCCATTTT	mmu-mir-96	10	chr6	30114875	30130825	13040	-	11.3973
CCCACCATTTT	mmu-mir-96	10	chr6	30114875	30130825	1119	-	11.2583
TTTTCCATTTT	mmu-mir-96	10	chr6	30114875	30130825	12154	-	10.7083
CCCGCCATGTA	mmu-mir-96	10	chr6	30114875	30130825	13759	+	10.5448
GTGTCCATCTT	mmu-mir-96	10	chr6	30114875	30130825	5942	-	10.5222
GTCGCCATCTG	mmu-mir-96	10	chr6	30114875	30130825	4365	-	10.522
CCCTCCATCTG	mmu-mir-96	10	chr6	30114875	30130825	3959	+	10.0338
CCTTCCATTTG	mmu-mir-96	10	chr6	30114875	30130825	12707	-	9.76748
TCCTCCATCTA	mmu-mir-96	10	chr6	30114875	30130825	396	+	9.73236
GACTCCATTTG	mmu-mir-96	10	chr6	30114875	30130825	14586	+	9.44683
GTGTCCATTAT	mmu-mir-96	10	chr6	30114875	30130825	11716	+	9.32754
TTCTCCATATT	mmu-mir-99a	10	chr16	77477393	77477593	50	-	10.8664
TCAACCATTTT	mmu-mir-99b	20	chr17	17530726	17533550	558	-	9.69437
CCCGCCATCCT	mmu-mir-99b	20	chr17	17530726	17533550	1094	-	9.67978
