# Supplementary material for: A Novel YY1-miR-1 Regulatory Circuit in Skeletal Myogenesis Revealed by Genome-Wide Prediction of YY1-miRNA Network
Source: PLoS One. 2012 Feb 1;7(2):e27596. doi: 10.1371/journal.pone.0027596 (PMC3271076; doi:10.1371/journal.pone.0027596)
Supplement: Table S2 — Up-regulated miRNAs during myoblasts differentiation into myotubes as revealed by miRNA microarray profiling. C2C12 myoblasts were grown in growth medium (GM) or differentiated in differentiation medium (DM) for 1 or 3 days. Total RNAs were extracted and subjected to expression profiling using a microarray platform. A total of 77 miRNAs were found to be up-regulated in DM 3 d as compared to GM. (PDF) [file pone.0027596.s007.pdf]

Suppl. Table S2: up-regulated miRNAs during C2C12 differentiation

miRNA	Fold Change (1d/0d)	Fold Change (3d/0d)	P.value	No. of YY1 site
mmu-mir-133b	57.80	73.53	<9.00E-04	1
mmu-mir-133a-1	51.55	68.49	<9.00E-04	1
mmu-mir-133a-2	40.32	46.08	<9.00E-04	0
mmu-mir-206	27.17	40.16	<9.00E-04	1
mmu-miR-615	17.45	31.06	<9.00E-04	0
mmu-miR-801	7.45	26.88	<9.00E-04	0
mmu-mir-128b	17.45	27.78	<9.00E-04	0
mmu-miR-699	3.87	15.46	<9.00E-04	0
mmu-miR-685	8.52	11.93	2.00E-04	0
mmu-mir-486	9.17	11.22	3.00E-04	0
mmu-mir-1-2	2.03	11.78	4.00E-04	1
mmu-mir-128a	4.05	9.89	5.00E-04	0
mmu-mir-196a-2	5.68	9.25	6.00E-04	5
mmu-mir-192	2.57	9.44	7.00E-04	6
mmu-miR-689	8.83	7.91	9.00E-04	0
mmu-mir-1-1	4.80	7.51	0.001	0
mmu-mir-378/422b	5.37	7.22	0.0011	0
mmu-mir-322	9.04	7.26	0.0012	20
mmu-mir-210	4.45	6.63	0.0016	0
mmu-miR-700	4.15	5.67	0.0023	0
mmu-mir-24-2	3.36	5.59	0.0026	21
mmu-mir-409	3.11	7.03	0.0027	4
mmu-mir-221	1.06	7.05	0.0033	33
mmu-mir-219-1	5.64	5.69	0.0036	0
mmu-miR-763	2.78	4.79	0.0044	0
mmu-miR-677	3.04	4.36	0.0054	0
mmu-miR-718	2.87	4.25	0.0055	0
mmu-mir-9*-1	1.49	4.83	0.0066	0
mmu-mir-30e	2.49	4.31	0.0068	4
mmu-miR-503	1.50	4.36	0.0075	0
mmu-mir-212	3.33	4.12	0.0079	0
mmu-mir-26a-2	3.56	3.84	0.0091	0
mmu-mir-132	3.69	4.08	0.0097	0
mmu-miR-673	3.54	3.93	0.0098	0
mmu-miR-181a	5.49	3.71	0.0116	0
mmu-mir-320	2.35	3.65	0.0119	7
mmu-miR-532	4.31	3.57	0.0123	0
mmu-mir-9-3	4.40	3.52	0.0142	21
mmu-mir-483	2.46	3.51	0.0158	0
mmu-miR-291b	2.71	3.45	0.0164	0
mmu-mir-103-2	3.34	3.35	0.0167	0
mmu-mir-351	5.54	3.29	0.0172	14
mmu-mir-93	2.10	3.24	0.0187	0
mmu-miR-684	2.42	3.17	0.0206	0
mmu-miR-500	3.97	3.18	0.0225	0
mmu-mir-199a-1	2.98	3.11	0.0225	8
mmu-mir-380	1.30	3.04	0.0231	4

mmu-mir-452	2.23	3.08	0.0236	0
mmu-mir-425	3.29	3.07	0.0238	7
mmu-mir-130b	3.10	3.04	0.0248	3
mmu-mir-145	2.09	3.03	0.0251	12
mmu-mir-22	0.68	3.05	0.0255	0
mmu-mir-471	2.16	2.95	0.0268	0
mmu-miR-760	2.08	2.98	0.0271	0
mmu-mir-199a	2.68	2.95	0.0276	0
mmu-miR-666	1.83	2.96	0.0279	0
mmu-mir-26a-1	2.91	2.96	0.028	0
mmu-mir-10a-2	2.59	2.94	0.0282	0
mmu-mir-214	5.14	2.88	0.0297	28
mmu-mir-135b	1.28	2.88	0.0308	0
mmu-mir-138-2	2.80	2.86	0.0318	1
mmu-mir-152	3.31	2.86	0.0319	0
mmu-miR-712*	3.14	2.81	0.034	0
mmu-mir-30a	1.37	2.66	0.0347	3
mmu-miR-542	1.27	2.80	0.035	0
mmu-mir-126	1.95	2.75	0.0361	1
mmu-mir-200b	1.75	2.73	0.0369	1
mmu-mir-15a	0.92	2.69	0.0383	4
mmu-mir-153	2.04	2.71	0.039	1
mmu-miR-762	1.42	2.72	0.0391	0
mmu-mir-381	1.10	2.73	0.0394	4
mmu-mir-199a*	3.33	2.72	0.0399	0
mmu-mir-130a	3.29	2.71	0.0402	23
mmu-mir-154	1.29	2.69	0.0415	4
mmu-miR-369	1.25	2.59	0.0451	0
mmu-mir-103-1	3.87	2.62	0.0457	0
mmu-mir-451	0.70	2.34	0.0492	1
