# Supplementary material for: A Novel YY1-miR-1 Regulatory Circuit in Skeletal Myogenesis Revealed by Genome-Wide Prediction of YY1-miRNA Network
Source: PLoS One. 2012 Feb 1;7(2):e27596. doi: 10.1371/journal.pone.0027596 (PMC3271076; doi:10.1371/journal.pone.0027596)
Supplement: Table S3 — Down-regulated miRNAs during myoblasts differentiation into myotubes as revealed by miRNA microarray profiling. A total of 68 miRNAs were found to be down-regulated with a fold change more than 2. (PDF) [file pone.0027596.s008.pdf]

Suppl. Table S3: down-regulated miRNAs during C2C12 differentiation

miRNA	Fold Change(0D/1D)	Fold Change(0D/3D)	P.value	No. of YY1 site
mmu-miR-703	5.4259	24.3523	<8.00E-04	0
mmu-mir-122a	3.5843	19.3278	1.00E-04	0
mmu-mir-9-2	3.2362	14.4929	2.00E-04	0
mmu-miR-805	3.5924	13.2678	3.00E-04	0
mmu-mir-29b-1	3.7504	12.7107	3.00E-04	2
mmu-miR-449b	3.9669	12.2016	3.00E-04	0
mmu-miR-592	3.6836	11.6687	4.00E-04	0
mmu-miR-496	5.6001	11.4824	5.00E-04	0
mmu-mir-129-2	0.7649	10.479	6.00E-04	5
mmu-mir-7-1	5.2118	10.7833	7.00E-04	0
mmu-mir-300	7.258	10.5427	8.00E-04	4
mmu-miR-687	8.1471	10.4911	8.00E-04	0
mmu-mir-365-2	2.5387	9.8789	0.001	12
mmu-miR-494	2.8363	8.9441	0.001	0
mmu-mir-433	0.4775	8.3455	0.0012	18
mmu-mir-302	6.1296	9.4508	0.0012	0
mmu-mir-32	5.4758	8.787	0.0013	1
mmu-miR-669a	3.1891	9.0165	0.0014	0
mmu-mir-325	3.8686	8.6743	0.0018	0
mmu-miR-698	6.6033	7.9124	0.0025	0
mmu-mir-31	0.8171	7.847	0.0026	6
mmu-mir-16-2	4.3828	6.7332	0.0027	8
mmu-miR-297b	5.8556	7.3065	0.0034	0
mmu-miR-680	0.5979	7.0996	0.0038	0
mmu-miR-376c	2.7226	6.9666	0.004	0
mmu-mir-29b-2	3.1347	6.6638	0.0042	21
mmu-miR-719	3.6066	6.0672	0.006	0
mmu-miR-302d	3.0507	5.8315	0.0073	0
mmu-mir-411	3.1451	5.7427	0.0074	4
mmu-miR-670	1.2051	5.8513	0.0077	0
mmu-mir-9*-3	0.7128	5.6343	0.0077	0
mmu-miR-717	4.7883	5.1024	0.0082	0
mmu-mir-342	1.7848	5.6335	0.0086	0
mmu-mir-361	2.6492	5.4034	0.0096	3
mmu-miR-672	3.1383	5.1708	0.0107	0
mmu-mir-7b	1.3742	5.2301	0.0109	3
mmu-mir-468	1.7745	5.0408	0.0118	0
mmu-miR-546	3.6489	5.1118	0.012	0
mmu-mir-16-1	2.0373	4.8582	0.0135	4
mmu-mir-190	1.1882	4.5834	0.0153	1
mmu-mir-96	2.3065	4.3196	0.0192	46
mmu-mir-466	1.3987	4.0936	0.0206	0
mmu-let-7c-1	1.1119	4.2009	0.0208	1
mmu-mir-196b	5.0618	4.1595	0.0212	22
mmu-mir-131	2.6259	3.9254	0.0235	0
mmu-mir-329	1.6735	3.9368	0.0246	4
mmu-miR-692	0.8617	3.1854	0.025	0
mmu-mir-382	3.1061	3.8382	0.0265	4

mmu-mir-449	1.592	3.8686	0.028	0
mmu-miR-678	1.8018	3.7911	0.0292	0
mmu-mir-19a	1.354	3.598	0.0302	17
mmu-mir-410	1.6863	3.8454	0.0305	4
mmu-miR-668	1.1462	3.7027	0.0308	0
mmu-mir-376a	2.4196	3.6813	0.0322	4
mmu-miR-181a*	1.0931	3.531	0.0326	0
mmu-miR-683	1.1713	3.6367	0.0331	0
mmu-mir-297-2	1.9146	3.7039	0.0332	0
mmu-mir-181b-1	1.1018	3.656	0.0341	1
mmu-mir-181b-2	2.0068	3.6969	0.0348	0
mmu-mir-148b	1.0225	3.4025	0.0376	3
mmu-miR-489	3.0282	3.4938	0.038	0
mmu-miR-499	1.1139	3.2691	0.0415	0
mmu-miR-539	1.9933	3.2786	0.0415	0
mmu-miR-691	2.7037	3.1482	0.0429	0
mmu-mir-338	1.8134	3.1257	0.0449	1
mmu-mir-200c	1.3136	3.1204	0.0453	13
mmu-mir-19b-1	4.1926	3.2924	0.0456	0
mmu-mir-138-1	1.2777	3.2328	0.0498	16
