# Supplementary material for: A Novel YY1-miR-1 Regulatory Circuit in Skeletal Myogenesis Revealed by Genome-Wide Prediction of YY1-miRNA Network
Source: PLoS One. 2012 Feb 1;7(2):e27596. doi: 10.1371/journal.pone.0027596 (PMC3271076; doi:10.1371/journal.pone.0027596)
Supplement: Table S4 — Computational prediction reveals the prevalence of YY1 binding sites on promoters of down-regulated miRNAs. The 68 down-regulated miRNAs were run through the YY1 binding site search pipeline. A total of 27 miRNAs were found to contain at least one YY1 binding site on their promoters. (PDF) [file pone.0027596.s009.pdf]

Suppl. Table S4: down-regulated miRNAs with YY1 predicted YY1 binding sites

miRNA	Fold Change (GM/DM)	P Value	No. of YY1 site
mmu-mir-29b-1	12.71	3.00E-04	2
mmu-mir-129-2	10.48	6.00E-04	5
mmu-mir-300	10.54	8.00E-04	4
mmu-mir-365-2	9.88	<8.00E-04	12
mmu-mir-433	8.35	<8.00E-04	18
mmu-mir-32	8.79	<8.00E-04	1
mmu-mir-31	7.85	<8.00E-04	6
mmu-mir-16-2	6.73	<8.00E-04	8
mmu-mir-29b-2	6.66	<8.00E-04	21
mmu-mir-411	5.74	0.01	4
mmu-mir-361	5.4	0.01	3
mmu-mir-7b	5.23	0.01	3
mmu-mir-16-1	4.86	0.01	4
mmu-mir-190	4.58	0.02	1
mmu-mir-96	4.32	0.02	46
mmu-let-7c-1	4.2	0.02	1
mmu-mir-196b	4.16	0.02	22
mmu-mir-329	3.94	0.02	4
mmu-mir-382	3.84	0.03	4
mmu-mir-19a	3.6	0.03	17
mmu-mir-410	3.85	0.03	4
mmu-mir-376a	3.68	0.03	4
mmu-mir-181b-1	3.66	0.03	1
mmu-mir-148b	3.4	0.04	3
mmu-mir-338	3.13	0.04	1
mmu-mir-200c	3.12	0.05	13
mmu-mir-138-1	3.23	0.05	16
