# Supplementary material for: A Novel YY1-miR-1 Regulatory Circuit in Skeletal Myogenesis Revealed by Genome-Wide Prediction of YY1-miRNA Network
Source: PLoS One. 2012 Feb 1;7(2):e27596. doi: 10.1371/journal.pone.0027596 (PMC3271076; doi:10.1371/journal.pone.0027596)
Supplement: Table S5 — Oligonucleotides used for RT-PCR, ChIP-PCR and cloning. (PDF) [file pone.0027596.s010.pdf]

Suppl. Table S5: Oligonucleotides used for RT-PCR, cloning, and ChIP-PCR.

Name	Sequence
F_mTnni2	CCTGAAGAGTGTGATGCTCCA
R_mTnni2	CCCGTTCCTTCTCAGTGTCTT
F_mMyh2	TCGTTTGCCAGTAAGGGTCT
R_mMyh2	TGCATCTTTGCTCTGAATGG
F_mActa1	CACCAGGGTGTCATGGTAGG
R_mActa1	TGGTACGGCCGGAAGCATAG
YY1-RT-F	ATGTGGTCCTCGGATGAAAA
YY1-RT-R	CTTTGTGCAGCCTTTATGAG
F_mGAPDH	CACGGCAAATTCAACGGCACAGTCAAGG
R_mGAPDH	GTTCACACCCATCACAAACATGG
F_mMyog	ATGGTGCCCAGTGAATGCAA
R_mMyog	ACCCAGCCTGACAGACAATC
primary miR1-2/133a-1-mib-F	AGACGTGGCTAAAGTGGAAGA
primary miR1-2/133a-1-mib-F	TATTACGAGCATTCAGGTCAGC
miRNA-1-2/133a-1-E1-F	GCATGGTACCGGAGGAGCAGGAGTTTGAGTTT
miRNA-1-2/133a-1-E1-R	GCATGCTAGCCCGCGTGAGGTTTTCCTAGT
miRNA-1-2/133a-1-E2-F	AAAAGGTACCGCTACATTAGTTATTCCACAGG
miRNA-1-2/133a-1-E2-R	AAAAGCTAGCCAACACAATCTCAGACAGTATGC
miRNA-1-1/133a-2-E3-F	AAAAGGTACCACACATCAGTCCCTCAGAAC
miRNA-1-1/133a-2-E3-R	AAAACTCGAGGATCCGTTTCCCAACATG
miR-206/133b-E4-F	AAAAGGTACCGGACTGATCCAAGGCTGTTCT
miR-206/133b-E4-R	AAAAGCTAGCTTCCTATTTGGGTTGCTTGG
muta-1-2/133a-1-E1-A-F	AAAATAGCAGGACAAGGCCCAAAGCAGTGTTGGG
muta-1-2/133a-1-E1-A-R	CCCAACACTGCTTTGGGCCTTGTCCTGCTATTTT
muta-1-2/133a-1-E1-B-F	GCTATGGCTGACTAAATGTCCTCAGGGAAGAGAGAG
muta-1-2/133a-1-E1-B-R	CTCTCTCTTCCCTGAGGACATTTAGTCAGCCATAGC
F_mMyHC_YY1 ChIP	CACATACCTACCTTTAAGGG
R_mMyHC_YY1 ChIP	CTCCATTCCCTATCAGATGC
F_mTnni2_YY1 ChIP	GTGAGGCCCAGCCCATCTTC
R_mTnni2_YY1 ChIP	CATTTTCCTGCTTGTGTGTC
YY1 ChIP_miRNA1-2/133a-1-E1-A-F	GGGAGAATCTGGGAAATGTA
YY1 ChIP_miRNA1-2/133a-1-E1-A-R	AAAGCTGAGGAGGATTCTAT
YY1 ChIP_miRNA1-2/133a-1-E1-B-F	AGCAAGATAGAATCCTCCTCA
YY1 ChIP_miRNA1-2/133a-1-E1-B-R	AGGCAGCTAAGCATTTGAAACA

YY1 ChIP_miRNA1-2/133a-1-E2-C-F	GACACTATCAGATGCTGGACT
YY1 ChIP_miRNA1-2/133a-1-E2-C-R	GCACTTGCCTGGTTCTATTT
YY1 ChIP_miRNA-206/133b-E4-E-F	AGTTCGGGACCCTTCTTCT
YY1 ChIP_miRNA-206/133b-E4-E-R	CAATCACCTGCGTCACAATC
ChIP-PCR-NC-F	GAAATGCTGTTAACCTGGCAG
ChIP-PCR-NC-R	GAGGCTGACCTTGGAGTCAC
mPax7-RT-F	CTCAGTGAGTTCGATTAGCC
mPax7-RT-R	CCAGACGGTTCCCTTTGT
Pax7-miR-1-siteA-top	CTAGTCATGTCTCCTGCCCTCAGCCAGCAAGACATTCCTAGAGAGAGGAACGAGCT
Pax7-miR-1-siteA-bottom	CGTTCCTCTCTCTAGGAATGTCTTGCTGGCTGAGGGCAGGAGACATGA
Pax7-miR-1-siteB-top	CTAGTCAGGGCCCAGTCCCAGCCAAGGGAGCAACATTCCAACGCTTGGACCAGAGCT
Pax7-miR-1-siteB-bottom	CTGGTCCAAGCGTTGGAATGTTGCTCCCTTGGCTGGGACTGGGCCCTGA
F_mMyoD	CTGCTCTGATGGCATGATGG
R_mMyoD	GTTCCCTGTTCTGTGTCGCT
YY1-3UTR-miR1-mut-top	CTAGTGCTAAAATGGGACTTCTTTTCGGGCCTTATAAATATGAAGCGAGCT
YY1-3UTR-miR1-mut-bottom	CGCTTCATATTTATAAGGCCCGAAAAGAAGTCCCATTTTTAGCA
